# Syntheses of dinor-*cis*/*iso*-12-oxo-phytodienoic acid (dn-*cis*/*iso*-OPDAs), ancestral jasmonate phytohormones of the bryophyte *Marchantia polymorpha* L., and their catabolites

**DOI:** 10.1038/s41598-021-81575-z

**Published:** 2021-01-21

**Authors:** Jianxin Wang, Haruka Sakurai, Nobuki Kato, Takuya Kaji, Minoru Ueda

**Affiliations:** 1 Department of Chemistry, Graduate School of Science, Tohoku University, Sendai, 980-8578 Japan; 2 Department of Molecular and Chemical Life Sciences, Graduate School of Life Sciences, Tohoku University, Sendai, 980-8578 Japan

**Keywords:** Natural product synthesis, Jasmonic acid, Natural product synthesis

## Abstract

In recent years, the biology of the evolutionary origin of phytohormone signaling has made significant progress. Among them, the ligand-receptor co-evolution found in jasmonate signaling has attracted the attention of plant scientists. Dinor-*cis-*12-oxo-phytodienoic acid (dn-*cis*-OPDA, **4**) and dn-*iso-*OPDA (**5**) are ancestral plant hormones of the bryophyte *Marchantia polymorpha* L. We succeeded in the first practical synthetic supply of these hormones as well as their possible catabolites. These compounds are expected to be useful in the study of ancestral jasmonate signaling in bryophytes.

## Introduction

**Figure 1:**
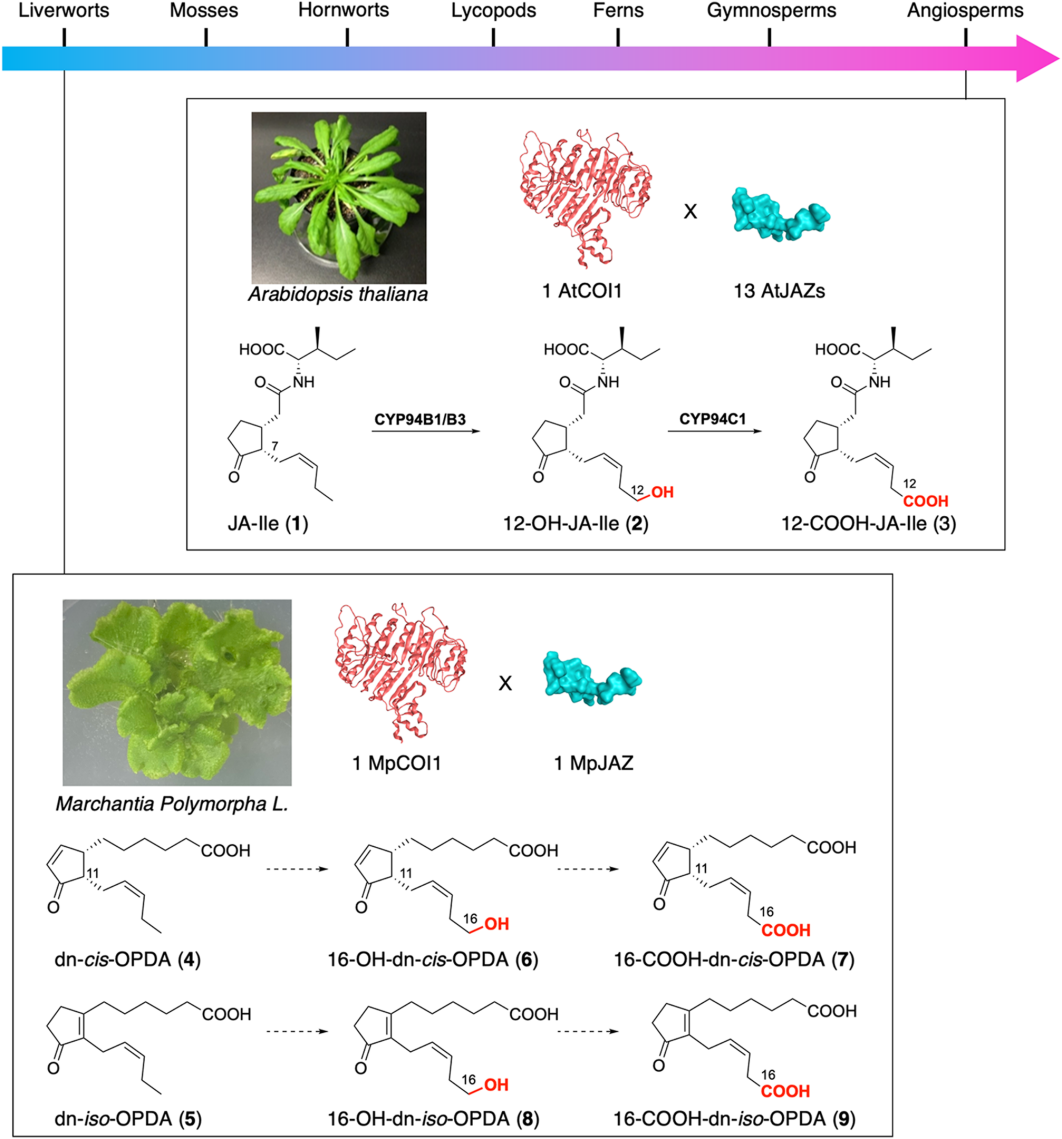
Ligand-receptor co-evolution of jasmonates, JA-Ile (**1**) in *Arabidopsis thaliana*, dn-*cis*-OPDA (**4**) and dn-*iso*-OPDA (**5**) in *Marchantia polymorpha* L., and their potent catabolites.

(+)-Jasmonoyl-l-isoleucine (JA-Ile, **1**) is a lipid-derived plant phytohormone, implicated in the regulation of plant growth, fertility, and defense against pathogens and insects^1–3^. JA-Ile-mediated signal transduction depends on the COI1-JAZ co-receptor system, composed of an F-box protein CORONATINE INSENSITIVE 1 (COI1) and JASMONATE ZIM-DOMAIN (JAZ) repressor protein^4–6^. After signal transduction, **1** is catabolized into 12-hydroxy-JA-Ile (12-OH-JA-Ile, **2**) by CYP94B1/B3, then 12-carboxy-JA-Ile (12-COOH-JA-Ile, **3**) by CYP94C1, and then deactivated within a few hours (Fig. 1)^7–9^. Biological studies of the evolution of phytohormone signaling is an important topic in plant science^10–13^, and recent achievements in the genomic sequencing of a myriad of plant species have enabled research into the evolutionary origins of phytohormones. *Marchantia polymorpha* L., a type of bryophyte, has attracted a great deal of attention due to its ancestral signaling module^14^, and its jasmonate signaling constitutes an intriguing example of ligand-receptor co-evolution, depending on the *Mp*COI1-*Mp*JAZ co-receptor system (Fig. 1)^15^. However, JA-Ile, the usual ligand for the COI1-JAZ co-receptor system of vascular plants, cannot be perceived by *Mp*COI1-*Mp*JAZ–dinor-*cis-*12-oxo-phytodienoic acid (dn-*cis*-OPDA, **4**) and dn-*iso-*OPDA (**5**) are the ligands of *Mp*COI1-*Mp*JAZ co-receptor instead (Fig. 1). Genetic studies have revealed that the ligand-receptor pair of dn-*cis*/*iso*-OPDA and *Mp*COI1-*Mp*JAZ participates in all the jasmonate responses of *M. polymorpha,* including defense responses^16–18^. However, biological studies require samples of **4** and **5**—in the past, **4** has been synthesized enzymatically^19^ but is now out of stock; and the only known synthesis of **5** entails an electroorganic reaction^20^ that cannot be accomplished using normal laboratory equipment (Scheme S1). Accordingly, we developed and report herein the first chemical synthesis of **4** and the first non-electroorganic synthesis of **5**. In addition, we synthesized their potent catabolites 16-hydroxy-dinor-*cis-*OPDA (16-OH-dn-*cis*-OPDA, **6**), 16-carboxy-dinor-*cis*-OPDA (16-COOH-dn-*cis*-OPDA, **7**), 16-hydroxy-dinor-*iso-*OPDA (16-OH-dn-*iso*-OPDA, **8**), 16-carboxy-dinor-*iso*-OPDA (16-COOH-dn-*iso*-OPDA, **9**) (Fig. 1). These potent catabolites will enable further studies on the catabolism of ancestral plant hormones.

## Results and discussion

### Synthesis of dn-*cis*-OPDA (4) and its potent catabolites (6 and 7)

**Scheme 1:**
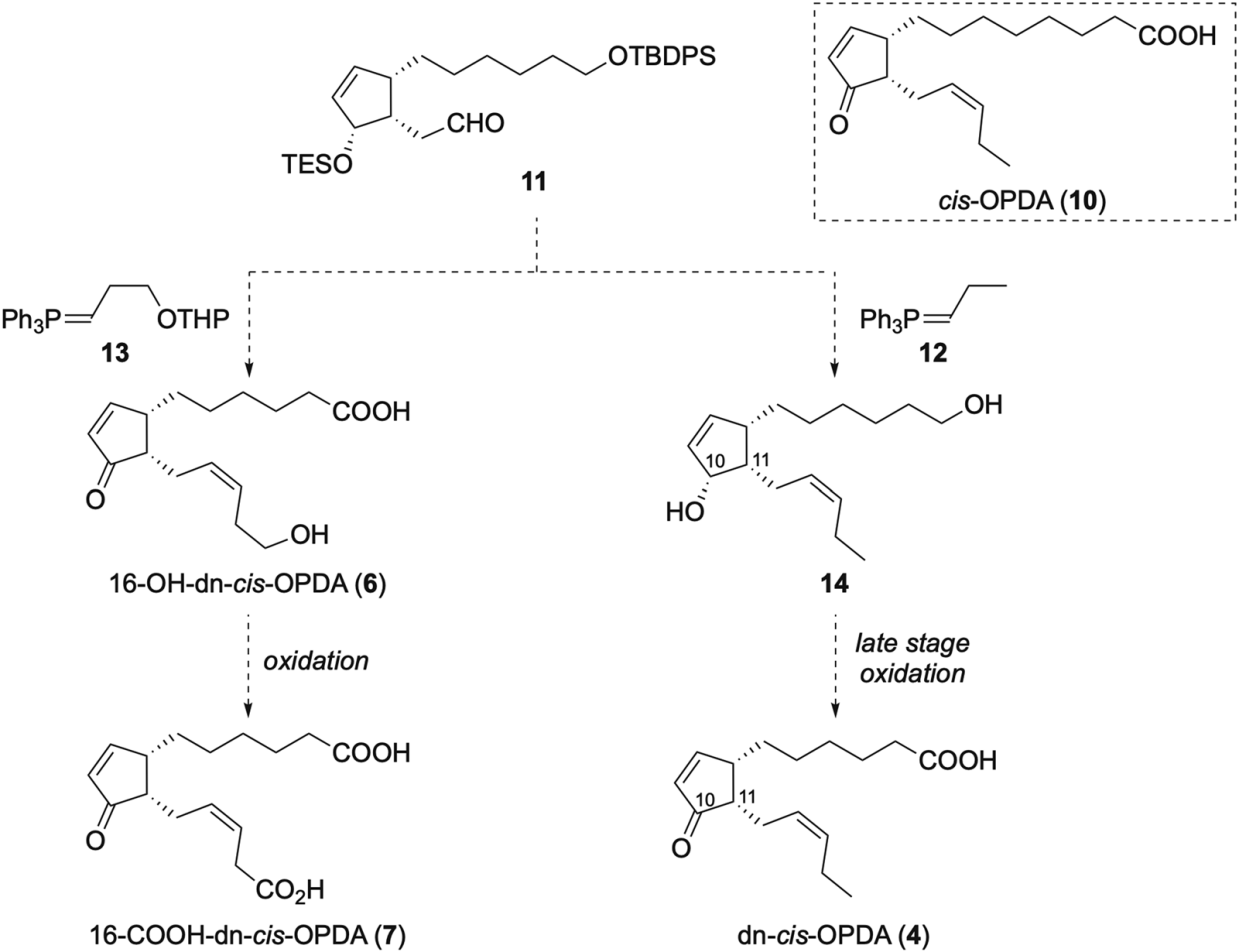
Synthetic plan for dn-*cis*-OPDA (**4**) and its potent catabolites (**6** and **7**).

Our plan for the synthesis of dn-*cis*-OPDA (**4**) and its catabolites (**6** and **7**) is outlined in Scheme 1. A major initial concern in the syntheses of **4**, **6** and **7** was the avoidance of epimerization at C11 which was anticipated to be facile in the presence of either acid or base based on the ready epimerization of the structurally similar **1** to give the more thermodynamically stable *trans*-**1** in a ratio of *trans*:*cis* = 95:5^21^. We therefore planned to synthesize **4** according to a procedure similar to that used to synthesize OPDA (**10**), a congener of **4**, developed by Kobayashi et al.^22^. They avoided epimerization by introducing the ketone at the late stage of synthesis^23^. Compounds **4** and **6** would be obtained from a common intermediate **11** by Wittig reaction using a different phosphonium salt (Scheme 1), and **7** would be obtained by oxidation of **6**.

**Scheme 2:**
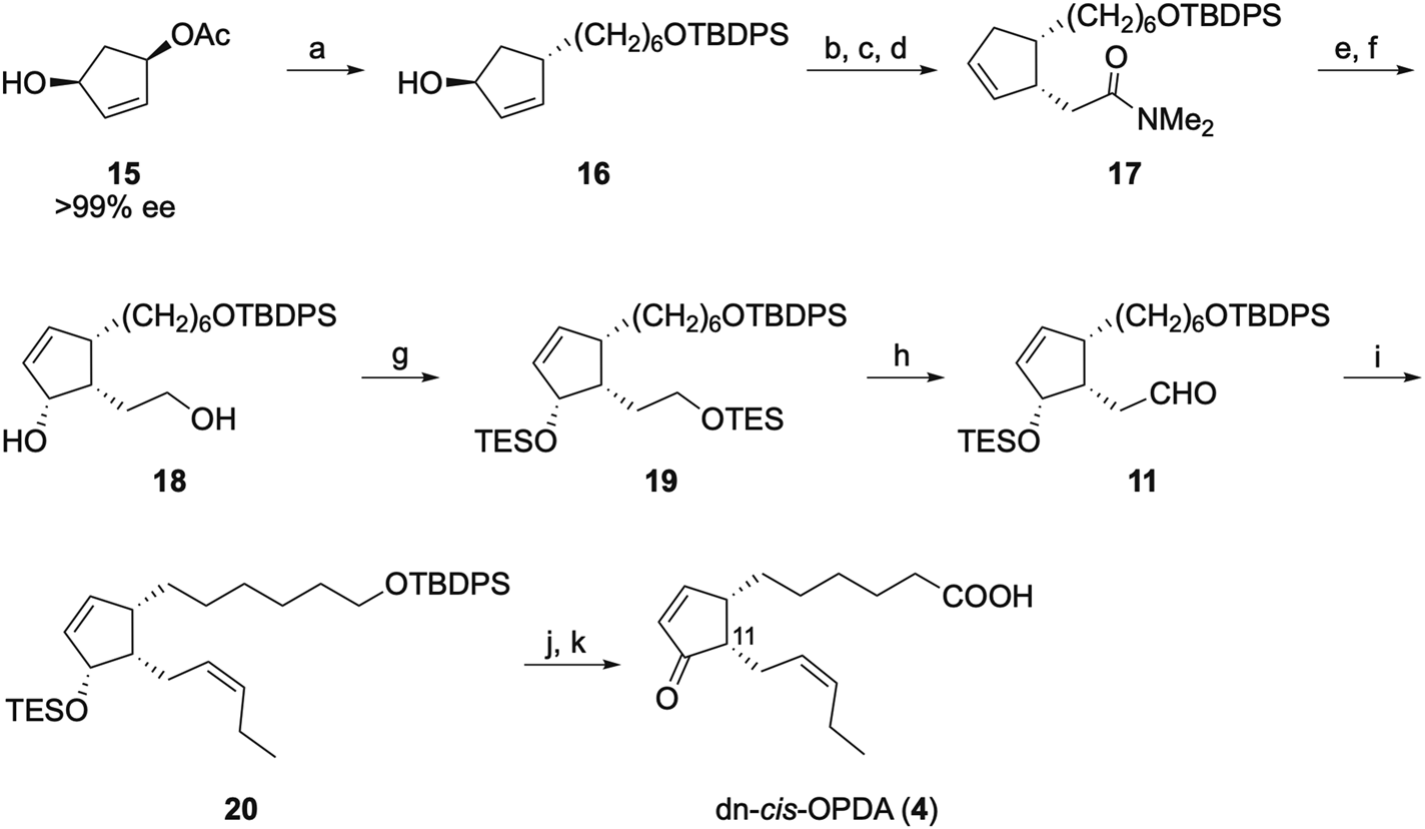
Synthesis of dn-*cis*-OPDA (**4**). Reagents and conditions: (**a**) TBDPSO(CH_2_)_6_MgCl,CuCN, THF, − 20 °C; (**b**) PPh_3_, AcOH, DIAD, toluene, − 20 °C; (**c**) LiOH, THF, MeOH, H_2_O; (**d**) MeC(OMe)_2_NMe_2_, xylene, reflux, 50% (4 steps); (**e**) I_2_, buffer (pH 5.0), THF; (**f**) DBU, THF, reflux; LiAlH_4_, 78% (2 steps); (**g**) TESCl, imadizole, DMF, 92%; (**h**) (COCl)_2_, DMSO; NEt_3_, CH_2_Cl_2_; (**i**) [Ph_3_PPr]^+^Br^−^, NaHMDS, THF, 71% (2 steps); (**j**) TBAF, THF, reflux, 91%; (**k**) Jones reagent, acetone, − 20 °C, 97%.

Our synthesis of dn-*cis*-OPDA (**4**) is summarized in Scheme 2. Allylic substitution of monoacetate **15**, prepared by enzymatic reaction^22^, with TBDPS(CH_2_)_6_MgCl, was performed in the presence of CuCN to afford **16**. The Mitsunobu reaction of **16**, hydrolysis of resulting acetate and Eschenmoser–Claisen rearrangement gave dimethylamide **17**. Iodolactonization, elimination with DBU and subsequent reduction with LiAlH_4_ afforded diol **18**. In the iodolactonization step, use of water in place of buffer resulted in the removal of TBDPS group. TES protection of diol **18** and regioselective Swern oxidation of the primary TESOCH_2_ group^24^ afforded the common intermediate **11**. In pursuit of **4**, **11** was treated with [Ph_3_PPr]^+^Br^−^ and NaHMDS to give diene **20**. Deprotection of the silyl groups of **20** with TBAF and subsequent Jones oxidation at − 20 °C afforded dn-*cis*-OPDA (**4**, 15 mg in 12 steps and 22% overall yield from **15**). In the work up of final Jones oxidation, removal of chromium compounds and sulfuric acid using silica gel caused epimerization of C11 (probably due to exothermic adsorption of sulfuric acid on silica gel)^22^, but this could be easily avoided by removal of the inorganic substances with water instead. The obtained **4** was quite stable at room temperature under neutral condition and no epimerization at C11 was observed even after 2 weeks^25–27^.

**Scheme 3:**
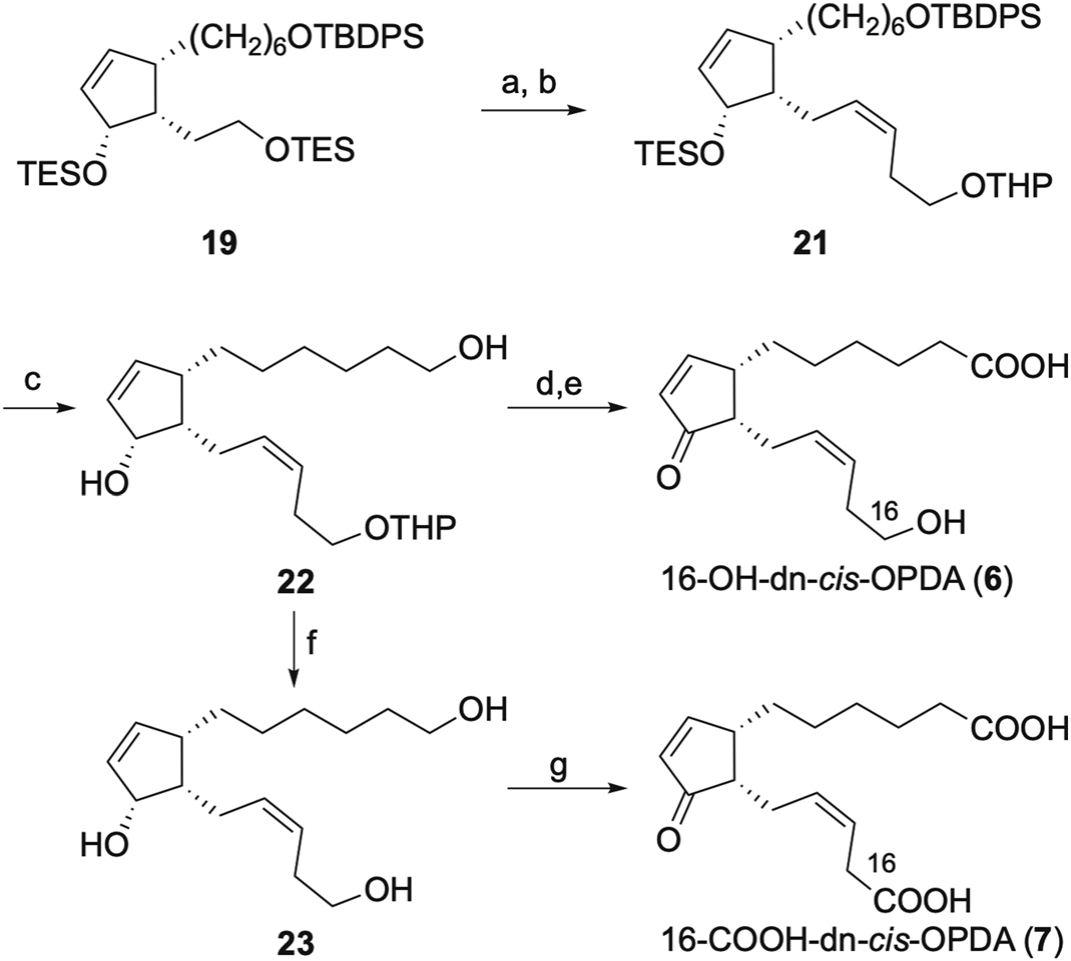
Synthesis of 16-OH-dn-*cis*-OPDA (**6**) and 16-COOH-dn-*cis*-OPDA (**7**). Reagents and conditions: (**a**) (COCl)_2_, DMSO, NEt_3_, CH_2_Cl_2_; (**b**) [THPO(CH_2_)_3_PPh_3_]^+^Br^-^, NaHMDS, THF, 76% (2 steps); (**c**) TBAF, THF 72%; (**d**) Jones reagent, acetone, − 20 °C, (**e**) MgBr_2_, Et_2_O, 72% (2 steps); (**f**) PPTS, MeOH, 35 °C, 54%; (**g**) Jones reagent, acetone, − 20 °C, 73%.

For the syntheses of 16-OH-dn-*cis*-OPDA (**6**) and 16-COOH-dn-*cis-*OPDA (**7**), [THPO(CH_2_)_3_PPh_3_]^+^Br^−^ was used in the Wittig reaction with common intermediate **11**, leading to diene **21**. Deprotection of the silyl groups of **21** with TBAF afforded diol **22**, from which the target products **6** and **7** could be obtained by oxidation and deprotection, or vice versa (Scheme 3). Jones oxidation of **22** and subsequent THP deprotection using MgBr_2_ afforded 16-OH-dn-*cis*-OPDA (**6**, 41 mg in 13 steps and 14% overall yield from **15**). In the THP deprotection step, some epimerization of C11 took place (*cis*:*trans* = ca. 94:6 by NMR). This epimerization and observed diastereomeric ratio were in good accordance with previous literature (*cis*:*trans* = ca. 92:8 in NMR)^22^. *cis*- and *trans*-**6** were easily separated by chiral HPLC using a CHIRALPAK IA column to obtain pure *cis*-**6** (22.3 mg). Conversely, THP deprotection of **22** using PPTS afforded triol **23**, Jones oxidation of which afforded 16-COOH-dn-*cis*-OPDA (**7**, 8.3 mg in 13 steps and 7.5% overall yield from **15**).

### Synthesis of dn-*iso*-OPDA (5) and its potent catabolites (8 and 9)

**Scheme 4:**
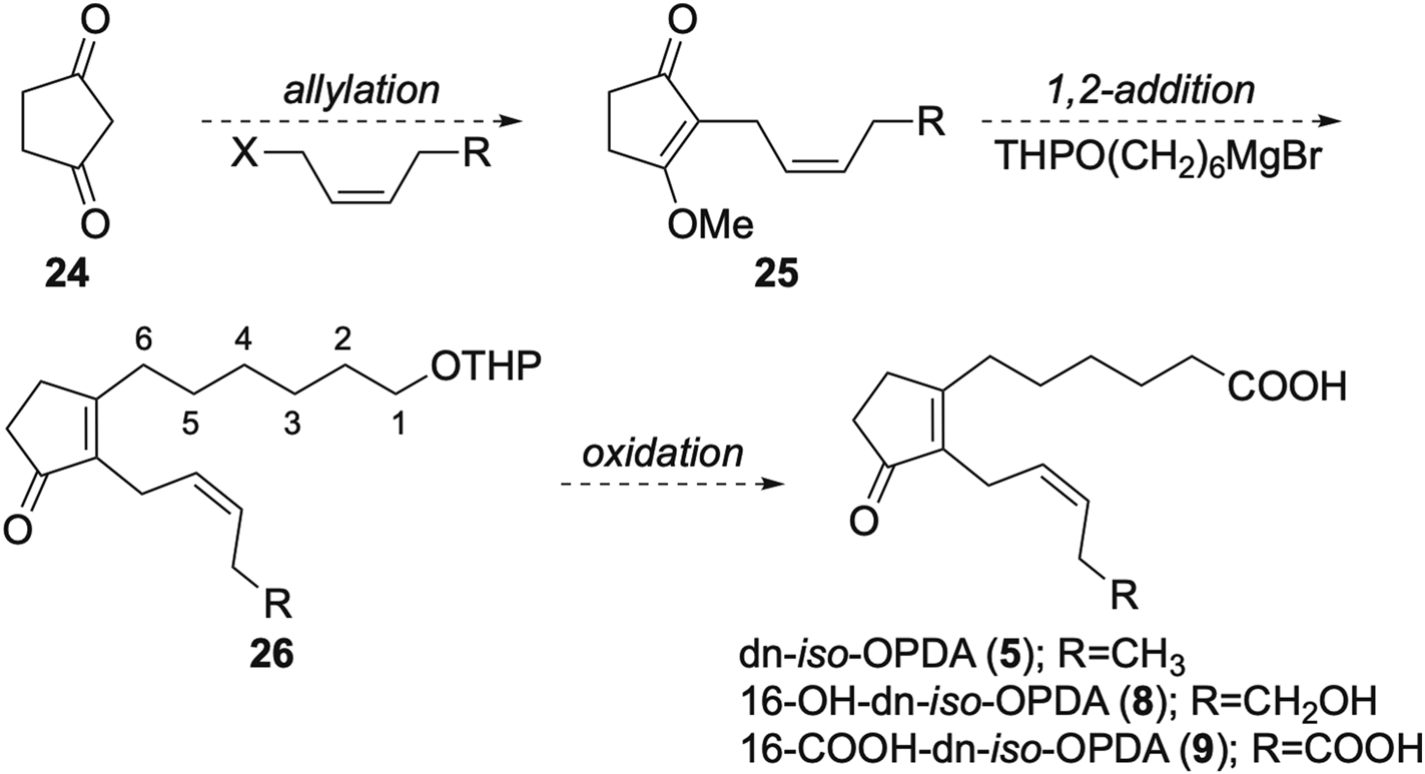
Synthetic plan for dn-*iso*-OPDA (**5**) and its potent catabolites (**8** and **9**).

Our plan for the synthesis of dn-*iso*-OPDA (**5**) and its potent catabolites (**8** and **9**) is shown in Scheme 4. In the synthesis of [^2^H_2_]-tetrahydrodicanenone (*iso*-OPDA), Lauchli and Boland Introduced the C1–8 side chain by the 1,4-addition using an organozinc reagent and CuCN^28^. However, organozinc reagent are difficult to prepare and CuCN is highly toxic. In contrast, Grignard reagents used for the 1,2-addition are easy to prepare and less toxic than CuCN. And potent catabolites 16-OH-dn-*iso*-OPDA (**8**) and 16-COOH-dn-*iso*-OPDA (**9**) could be obtained from the same starting material **24** by using a different allyl bromide.

**Scheme 5:**
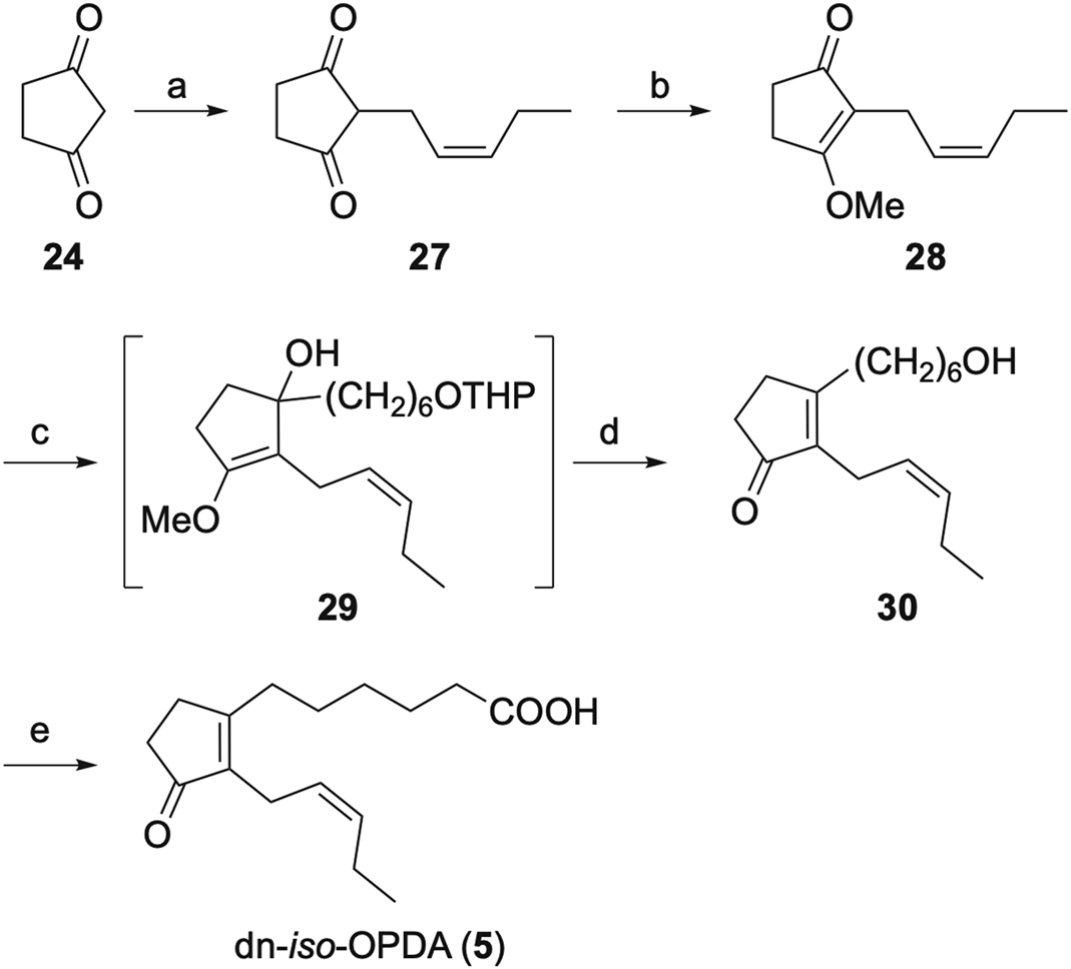
Synthesis of dn-*cis*-OPDA (**5**). *Reagents and conditions*: (**a**) *cis*-1-bromopent-2-ene, K_2_CO_3_, H_2_O, 60 °C, 25%; (**b**) Me_2_SO_4_, K_2_CO_3_, acetone, reflux, quant.; (**c**) THPO(CH_2_)_6_MgBr, THF, reflux (**d**) 2 M HCl aq., 24%; (**e**) Jones reagent, acetone, 0 °C, 89%.

Our synthesis of dn-*iso*-OPDA (**5**) is summarized in Scheme 5. Allylation of 1,3-cyclopentanedione **24** followed by methylation of the resulting **27** gave cyclopentenone **28**. After the Grignard reaction of **28**, dilution of the reaction mixture with hydrochloric acid promoted hydrolysis of the enol ether and the deprotection of the THP group, to give alcohol **30**. Finally, Jones oxidation of **30** gave 61 mg of dn-*iso*-OPDA (**5**) in only 4 steps from **24**.

**Scheme 6:**
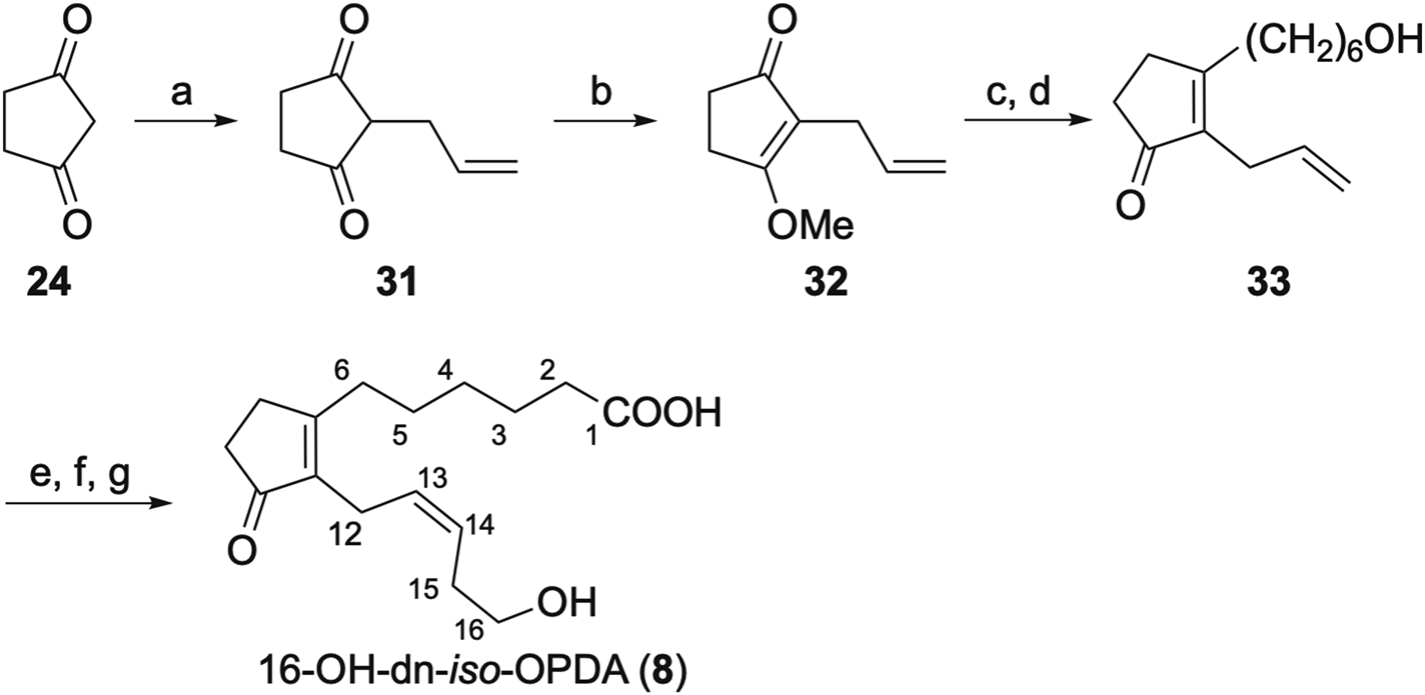
Synthesis of 16-OH dn-*iso*-OPDA (**8**). Reagents and conditions: (**a**) allyl acetate, [Pd(C_3_H_5_)Cl]_2_, dppe, BSA, NaOAc, THF, reflux, 81%; (**b**) Me_2_SO_4_, K_2_CO_3_, acetone, reflux, 98%; (**c**) THPO(CH_2_)_6_MgBr, THF, reflux; (**d**) 1 M HCl aq., reflux, 36% (2 steps); (**e**) Hoveyda–Grubbs catalyst M2001 Umicore, CH_2_ = CHCH_2_CH_2_OAc; (**f**) Jones reagent, acetone, − 20 °C; (**g**) LiOH, H_2_O, THF, 40% (3 steps).

**Scheme 7:**
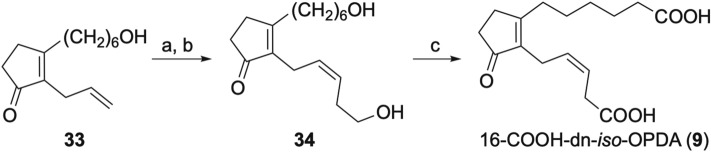
Synthesis of 16-COOH-dn-*iso*-OPDA (**9**). *Reagents and conditions*: (**a**) Hoveyda-Grubbs Catalyst M2001 Umicore, CH_2_=CHCH_2_CH_2_OAc. (**b**) NaOH, H_2_O, MeOH, 50 °C 32% (2 steps). (**c**) Jones reagent, acetone, − 20 °C, 36%.

Next, we synthesized 16-OH-dn-*iso*-OPDA (**8**) and 16-COOH-dn-*iso*-OPDA (**9**) (Schemes 6, 7). Introduction of the C12–C16 side chain of **24** was first attempted by allylation, but the side chain could not be directly introduced using allylation in order to O-alkylation. Accordingly, we abandoned this approach and sought to construct the C12–C16 side chain by *Z*-selective cross metathesis^29–31^. Pd-mediated allylation^32^ of **24** and subsequent methylation gave allylcyclopentenone **32**. Introduction of the C1–C6 side chain using THPO(CH_2_)_6_MgBr followed by hydrolysis and deprotection of THP group gave alcohol **33**. Finally, Jones oxidation of **33** followed by cross metathesis with CH_2_=CHCH_2_CH_2_OAc and deprotection of the acetyl group gave 16-OH-dn-*iso*-OPDA (**8**, 15.6 mg in 7 steps and 11% overall yield from **24**) as a *Z*/*E* mixture (10:1 *Z*/*E*). *Z*/*E* isomers were easily separated by chiral HPLC using a CHIRALPAK IA column to obtain pure *Z-8* (2.4 mg). 16-COOH-dn-*iso*-OPDA (**9**, 12:1 *Z*/*E*, 6.3 mg) was also obtained from allylcyclopentenone **33** by cross metathesis with CH_2_=CHCH_2_CH_2_OAc, hydrolysis of acetyl group and Jones oxidation (total 7 steps and 3.4% overall yield from **24**). *Z*/*E* isomers were easily separated by HPLC to obtain pure **Z-9** (2.6 mg).

## Conclusions

A synthetic supply of jasmonates is indispensable if their study is to be advanced^20,30,33,34^, and this work finally enables dn-*cis*/*iso*-OPDAs (**4** and **5**) and their potent catabolites (**6**–**9**) to be readily obtained. Our work is expected to accelerate biological studies of the signaling mechanisms of bryophyte hormones, which should lead to a better understanding of the evolutional origins of phytohormone signaling. In particular, study of the catabolism of **4** and **5** should provide insights into the deactivation mechanism of ancestral plant hormones. Biological studies using synthetic **4**–**9** are now in progress.

## Method

### Synthesis of dn-*cis*-OPDA (4)

To a solution of **20** (57.2 mg, 94.5 µmol) in THF (20 mL) was added 1 M TBAF in THF (1.6 mL, 1.60 mmol). After being stirred at reflux temperature for 1.5 h, the solvent was removed under reduced pressure. The residue was purified by medium-pressure chromatography (Isolera, eluent: 93:7 *n*-hexane/EtOAc to 40:60 *n*-hexane/EtOAc) to give a diol intermediate (22.3 mg, 94%) as a colorless oil. [α]_D_^22^ + 63.6 (*c* 1.09, CHCl_3_). ^1^H NMR (400 MHz, CDCl_3_) δ_H_: 6.22 (dd, *J* = 5.8, 2.6 Hz, 1H), 5.96 (brd, *J* = 5.8 Hz, 1H), 5.49–5.38 (m, 2H), 4.51 (dd, *J* = 5.8, 2.6 Hz, 1H), 3.63 (t, *J* = 6.6 Hz, 2H), 2.52–2.43 (m, 1H), 2.37–2.27 (m, 1H), 2.24–2.04 (m, 4H), 1.66–1.52 (m, 3H), 1.47–1.20 (m, 7H), 0.99 (t, *J* = 7.5 Hz, 3H); ^13^C NMR (100 MHz, CDCl_3_) δ_C_: 141.64, 132.46, 132.01, 127.88, 76.55, 62.99, 46.13, 46.00, 33.52, 32.73, 29.70, 28.05, 25.69, 23.08, 20.78, 14.26; IR (neat) cm^–1^: 3356, 2931, 2861, 1458, 1053; HRMS (ESI, positive) *m/z* [M + Na]^+^ Calcd. for C_16_H_28_NaO_2_: 275.1987, Found: 275.1976.

To a solution of diol intermediate (15.3 mg, 60.6 µmol) in acetone (5.2 mL) was added Jones reagent (4.0 M solution) at − 20 °C until the orange color of the reagent persisted (30 drops). After 10 min of stirring at − 20 °C, *i*-PrOH was added to quench the remaining reagent. Then, EtOAc/*n*-hexane (1/1, 20 mL) and H_2_O (20 mL) were added and the water layer was extracted with EtOAc. The combined organic layers were washed with saturated aqueous NaCl, dried over Na_2_SO_4_ and concentrated under reduced pressure. The residue was purified by medium-pressure chromatography (Isolera, eluent: 0.1:88:12 AcOH/*n*-hexane/EtOAc to 0.1:99.9 AcOH/EtOAc) to give **4** (116 mg, 97%) as a colorless oil. Diastereomeric purity of **4** was > 99% by ^1^H NMR spectroscopy (δ_H_ = 7.72 (dd, *J* = 5.8, 2.7 Hz, 1H) for **4**; 7.59 (dd, *J* = 5.8, 2.6 Hz, 1 H) for the *trans* isomer). [α]_D_^22^ + 135.5 (*c* 0.75, CHCl_3_). ^1^H NMR (400 MHz, CDCl_3_) δ_H_: 7.72 (dd, *J* = 5.8, 2.7 Hz, 1H), 6.18 (dd, *J* = 5.8, 1.6 Hz, 1H), 5.48–5.31 (m, 2H), 3.04–2.94 (m, 1H), 2.56–2.41 (m, 2H), 2.36 (brt, *J* = 7.1 Hz, 2H), 2.13 (dt, *J* = 15.8, 7.8 Hz, 1H), 2.05 (quintet, *J* = 7.5 Hz, 2H), 1.80–1.69 (m, 1H), 1.63 (brt, *J* = 7.1 Hz, 2H), 1.52–1.29 (m, 4H), 1.23–1.11 (m, 1H), 0.97 (t, *J* = 7.5 Hz, 3H); ^13^C NMR (100 MHz, CDCl_3_) δ_C_: 210.86, 179.32, 166.96, 133.02, 132.50, 126.82, 49.79, 44.20, 33.84, 30.58, 29.18, 27.32, 24.49, 23.76, 20.78, 13.99; IR (neat) cm^–1^: 3487, 3178, 2935, 1709; HRMS (ESI, positive) *m/z* [M + Na]^+^ Calcd. for C_16_H_24_NaO_3_: 287.1623, Found: 287.1618.

### Synthesis of 16-OH-dn-*cis*-OPDA (6)

To a solution of **22** (71.0 mg, 201 µmol) in acetone (20 mL) was added Jones reagent (4.0 M solution, 400 µL, 1.60 mmol) at − 20 °C. After 20 min of stirring at − 20 °C, *i*-PrOH was added to quench the remaining reagent. Then, EtOAc/*n*-hexane (1/1, 10 mL) and H_2_O (20 mL) were added and the water layer was extracted with EtOAc. The combined organic layers were washed with saturated aqueous NaCl, dried over Na_2_SO_4_ and filtered. The reaction mixture was concentrated under reduced pressure to give a carboxylic acid intermediate (86.3 mg, mixture) as a colorless oil. The crude product was used for the next reaction without further purification. To a solution of the carboxylic acid intermediate (86.3 mg, mixture) in Et_2_O (10 mL) was added MgBr_2_ (90.5 mg, 510 µmol). The solution was stirred at room temperature for 20 min and diluted with Et_2_O and MeOH. Then, H_2_O was added and the water layer was extracted with AcOH/EtOAc (1/999). The combined organic layers were washed with saturated aqueous NaCl, dried over Na_2_SO_4_ and filtered. The residue was purified by medium-pressure chromatography (Isolera, eluent: 0.1:84:16 AcOH/*n*-hexane/EtOAc to 0.1:99.9 AcOH/EtOAc) to give **6** (40.8 mg, 72% *cis*/*trans* = 92/8 mixture) as a colorless oil. *cis*- and *trans*-**6** were separated by chiral HPLC using a CHIRALPAK IA column (Daicel Co., Ltd., Japan, *Φ*20 × 250 mm, eluent: 0.1:20:80 AcOH/EtOH/*n*-hexane, flow rate: 8.0 mL/min, *cis*-**6**: Rt = 33.3 min, *trans*-**6**: Rt = 23.3 min) to afford pure *cis*-**6** (22.3 mg) as a colorless oil. [α]_D_^20^ + 34.8 (*c* 1.95, CHCl_3_). ^1^H NMR (400 MHz, CDCl_3_) δ_H_: 7.72 (dd, *J* = 5.8, 2.7 Hz, 1H), 6.18 (dd, *J* = 5.8, 1.6 Hz, 1H), 5.48–5.31 (m, 2H), 3.04–2.94 (m, 1H), 2.56–2.41 (m, 2H), 2.36 (brt, *J* = 7.1 Hz, 2H), 2.13 (dt, *J* = 15.8, 7.8 Hz, 1H), 2.05 (quintet, *J* = 7.5 Hz, 2H), 1.80–1.69 (m, 1H), 1.63 (brt, *J* = 7.1 Hz, 2H), 1.52–1.29 (m, 4H), 1.23–1.11 (m, 1H), 0.97 (t, *J* = 7.5 Hz, 3H); ^13^C NMR (100 MHz, CDCl_3_) δ_C_: 210.86, 179.32, 166.96, 133.02, 132.50, 126.82, 49.79, 44.20, 33.84, 30.58, 29.18, 27.32, 24.49, 23.76, 20.78, 13.99; IR (neat) cm^–1^: 2935, 2873, 1107; HRMS (ESI, positive) *m/z* [M + Na]^+^ Calcd. for C_16_H_24_NaO_4_: 303.1572, Found: 303.1565.

### Synthesis of 16-COOH-dn-*cis*-OPDA (7)

To a solution of **23** (10.3 mg, 38.4 µmol) in acetone (6.0 mL) was added Jones reagent (4.0 M solution) at − 20 °C until the orange color of the reagent persisted (37 drops). After 20 min of stirring at − 20 °C, *i*-PrOH was added to quench the remaining reagent. Then, EtOAc (10 mL) and H_2_O (60 mL) were added and the water layer was extracted with EtOAc. The combined organic layers were washed with saturated aqueous NaCl, dried over Na_2_SO_4_ and concentrated under reduced pressure. The residue was purified by medium-pressure chromatography (Isolera, eluent: 0.1:98:2 AcOH/CHCl_3_/MeOH to 0.1:80:20 AcOH/CHCl_3_/MeOH) to give **7** (8.3 mg, 73%) as a colorless oil. Diastereomeric purity of **7** was > 98% by ^1^H NMR spectroscopy (δ_H_ = 7.74 (dd, *J* = 5.9, 2.7 Hz, 1H) for **7**; 7.62 (dd, *J* = 5.9, 2.4 Hz, 1 H) for the *trans* isomer). [α]_D_^21^ + 117.6 (*c* 0.24, CHCl_3_). ^1^H NMR (400 MHz, CDCl_3_) δ_H_: 7.74 (dd, *J* = 5.9, 2.7 Hz, 1H), 6.18 (dd, *J* = 5.9, 1.6 Hz, 1H), 5.68–5.56 (m, 2H), 3.21 (dd, *J* = 17.2, 5.9 Hz, 1H), 3.11 (dd, *J* = 17.2, 3.9 Hz, 1H), 3.04–2.93 (m, 1H), 2.56–2.46 (m, 2H), 2.36 (brt, *J* = 7.1 Hz, 2H), 2.20 (ddd *J* = 16.7, 10.7, 6.1 Hz, 1H), 1.78–1.54 (m, 3H), 1.52–1.22 (m, 4H), 1.22–1.07 (m, 1H); ^13^C NMR (100 MHz, CDCl_3_) δ_C_: 210.45, 179.82, 177.53, 167.19, 132.47, 131.97, 121.59, 49.21, 44.26, 33.82, 32.94, 30.48, 29.03, 27.30, 24.24, 24.15; IR (neat) cm^–1^: 3448, 3205, 3031, 2935, 1708 (br); HRMS (ESI, positive) *m/z* [M + Na]^+^ Calcd. for C_16_H_22_NaO_5_: 317.1365, Found: 317.1355.

### Synthesis of dn-*iso*-OPDA (5)

To a solution of THPO(CH_2_)_6_MgBr (0.85 M in THF, 3.3 mL, 2.80 mmol) was added a solution of **28** (198 mg, 1.10 mmol) in THF (6.5 mL) at reflux temperature under argon atmosphere. After being stirred at 60 °C for 3 h, the reaction mixture was allowed to cool to rt and 2 M HCl aq. (7 mL) was added. After 1.5 h of stirring, H_2_O was added and the water layer was extracted with EtOAc. The combined organic layers were washed with saturated aqueous NaCl, dried over Na_2_SO_4_ and concentrated under reduced pressure. After evaporation, the residue was purified by medium-pressure chromatography (Isolera, eluent: 40:60 *n*-hexane/EtOAc to EtOAc) to give the oxidized compound. The compound was carried on to the next step.

To a solution of the mixture (79.6 mg) in acetone (6 mL) was added Jones reagent (4.0 M solution, 200 µL, 800 µmol) at 0 °C. After 3.5 h of stirring at 0 °C, *i*-PrOH was added to quench the remaining reagent. Then, H_2_O (20 mL) was added and the water layer was extracted with EtOAc. The combined organic layers were washed with saturated aqueous NaCl, dried over Na_2_SO_4_ and concentrated under reduced pressure. The residue was purified by medium-pressure chromatography (Isolera, eluent: 0.5:50:50 AcOH/*n*-hexane/EtOAc to 0.5:99.5 AcOH/EtOAc) to give **5** (61.4 mg, 89%) as a colorless oil. ^1^H NMR (400 MHz, CDCl_3_); δ_H_ 5.37 (ddt, *J* = 18.0, 7.2, 1.2 Hz, 1H), 5.21 (ddt, *J* = 17.6 7.2, 1.2 Hz, 1H), 2.93 (d, *J* = 7.2 Hz, 2H), 2.50 (t, *J* = 4.4 Hz, 2H), 2.44 (t, J = 7.6 Hz, 2H), 2.39–2.35 (m, 4H), 2.15 (quin, *J* = 7.6 Hz, 2H), 1.68 (quin, *J* = 6.4 Hz, 2H), 1.58 (quin, *J* = 8.0 Hz, 2H), 1.44–1.36 (m. 2H), 0.99 (t, *J* = 7.6 Hz, 3H); ^13^C NMR (100 MHz, CDCl_3_); δ_C_ 209.56, 178.73, 174.01, 139.39, 132.34, 125.32, 34.19, 33.71, 31.10, 29.15, 29.12, 27.08, 24.44, 21.24, 20.61, 14.14; IR (neat) cm^–1^: 3502, 2935, 2866, 1697, 1631; HRMS (ESI, positive) *m/z* [M + Na]^+^ Calcd. for C_16_H_24_O_3_: 287.1623, found: 287.1601.

### Synthesis of 16-OH-dn-*iso*-OPDA (8)

A 5 mL pear-shaped flask was charged with **33** (31.2 mg, 140 µmol), CH_2_ = CHCH_2_CH_2_OAc (165 mg, 144 mmol) and Hoveyda–Grubbs Catalyst M2001 Umicore (4.6 mg, 7.27 µmol). After 24 h of stirring, the residue was roughly purified by medium-pressure chromatography (Isolera, eluent: 0.1:99:1 AcOH/CHCl_3_/MeOH to 0.1:90:10 AcOH/CHCl_3_/MeOH) to give a mixture (58.2 mg). The crude mixture was used for the next reaction without further purification. To a solution of the mixture (58.2 mg) in acetone (10 mL) was added Jones reagent (4.0 M solution, 95 µL, 380 µmol) at − 20 °C. After 30 min of stirring at − 20 °C, *i*-PrOH was added to quench the remaining reagent. Then, EtOAc and H_2_O were added and the water layer was extracted with EtOAc. The combined organic layers were washed with saturated aqueous NaCl, dried over Na_2_SO_4_ and concentrated under reduced pressure. The crude mixture (33.0 mg) was used for the next reaction without further purification. To a solution of the mixture (33.0 mg) in THF (4.5 mL) was added 1 M-LiOH solution (510 µL, 510 µmol) and the mixture was stirred for 1.5 h. The reaction mixture was quenched with 1 M HCl aq. and the aqueous layer was extracted with EtOAc. The organic layer was washed with saturated aqueous NaCl, dried over Na_2_SO_4_, and filtered. After evaporation, the residue was purified medium-pressure chromatography (Isolera, eluent: 0.1:85:15 AcOH/*n*-hexane/EtOAc to 0.1:99.9 AcOH/ EtOAc) to give 16-OH-dn-iso-OPDA (**8**) (15.6 mg, 40% in 3 steps) as a *Z*/*E* mixture (*Z/E* = 10/1). *Z/E* isomers were separated by chiral HPLC using a CHIRALPAK IA column (Daicel Co., Ltd., Japan, *Φ*20 × 250 mm, eluent: 0.1:20:80 AcOH/EtOH/*n*-hexane, flow rate: 8.0 mL/min, *E*-**8**: Rt = 18.8 min, *Z*-**8**: Rt = 25.5 min) to afford pure *Z*-**8** (2.4 mg) as a colorless oil. ^1^H NMR (400 MHz, CDCl_3_) δ_H_: 5.43 (dt, *J* = 11.0, 7.2 Hz, 1H), 5.37 (dt, *J* = 11.0, 6.6 Hz, 1H), 3.71 (t, *J* = 6.2 Hz, 2H), 2.98 (d, *J* = 6.6 Hz, 2H), 2.52–2.50 (m, 2H), 2.49–2.44 (m, 4H) 2.38–2.36 (m, 4H), 1.69 (quintet, *J* = 7.6 Hz, 2H), 1.57 (quintet, *J* = 7.6 Hz, 2H), 1.40 (quintet, *J* = 7.6 Hz, 2H); ^13^C NMR (100 MHz, CDCl_3_) δ_C_:210.23, 178.34, 174.92, 138.75, 128.63, 126.82, 61.97, 34.18, 33.69, 31.03, 30.71, 29.27, 28.94, 27.07, 24.35, 21.71; IR (neat) cm^-1^: 3433, 2931, 2866, 1701, 1631; HRMS (ESI, positive) *m/z* [M + Na]^+^ Calcd. for C_16_H_24_NaO_4_: 303.1572, found: 303.1565.

### Synthesis of 16-COOH-dn-*iso*-OPDA (9)

To a solution of **35** (16.1 mg, 60.4 µmol) in acetone (1.5 mL) was added Jones reagent (4.0 M solution, 150 µL, 600 µmol) at − 20 °C. After 1.5 h of stirring at − 20 °C, *i*-PrOH was added to quench the remaining reagent. Then, EtOAc and H_2_O were added and the water layer was extracted with EtOAc. The combined organic layers were washed with saturated aqueous NaCl, dried over Na_2_SO_4_ and concentrated under reduced pressure. The residue was purified by medium-pressure chromatography (Isolera, eluent: 0.1:98:2 AcOH/CHCl_3_/MeOH to 0.1:80:20 AcOH/CHCl_3_/MeOH) to give 16-COOH-dn-*iso*-OPDA (**9**) (6.3 mg, 36%) as a *Z*/*E* mixture (*Z/E* = 12/1). *Z*/*E* isomers were separated by HPLC using a Cholester column (Nacalai Tesque, Inc., Japan, *Φ*20 × 250 mm, eluent: 0.1:30:70 AcOH/MeCN/H_2_O, flow rate: 8.0 mL/min, *E*-**9**: Rt = 25.0 min, *Z*-**9**: Rt = 25.9 min) to afford pure *Z*-**9** (2.6 mg) as a colorless oil. ^1^H NMR (400 MHz, CDCl_3_) δ_H_: 5.57 (dtt, *J* = 10.6, 7.3, 1.5 Hz, 1H), 5.48 (dtt, *J* = 10.6, 7.4, 1.3 Hz, 1H), 3.27 (d, *J* = 7.3 Hz, 2H), 2.96 (d, *J* = 7.4 Hz, 2H), 2.53–2.51 (m, 2H), 2.44 (t, *J* = 7.7 Hz, 2H), 2.40–2.35 (m, 4H), 1.66 (quintet, *J* = 7.7 Hz, 2H), 1.55 (quintet, *J* = 7.7 Hz, 2H), 1.38 (quintet, *J* = 7.7 Hz, 2H); ^13^C NMR (100 MHz, CDCl_3_) δ_C_: 209.72, 179.46, 177.13, 175.07, 138.16, 129.97, 121.01, 34.13, 33.71, 32.95, 31.08, 29.27, 28.94, 27.05, 24.23, 21.58; IR (neat) cm^−1^: 3518, 3190, 2935, 1705, 1628; HRMS (ESI, positive) *m/z* [M + Na]^+^ Calcd. for C_16_H_22_NaO_5_: 317.1365, found: 317.1340.

## Supplementary Information

Supplementary Information.
