# Supplementary material for: Syntheses of dinor-cis/iso-12-oxo-phytodienoic acid (dn-cis/iso-OPDAs), ancestral jasmonate phytohormones of the bryophyte Marchantia polymorpha L., and their catabolites
Source: Sci Rep. 2021 Jan 21;11:2033. doi: 10.1038/s41598-021-81575-z (PMC7820489; doi:10.1038/s41598-021-81575-z)
Supplement: Supplementary file 1 — Supplementary Information. [file 41598_2021_81575_MOESM1_ESM.pdf]

Syntheses of dinor-*cis*/*iso*-12-oxo-phytodienoic acid (dn-*cis*/*iso*-OPDAs), ancestral jasmonate phytohormones of the bryophyte, *Marchantia polymorpha* L., and their catabolites

Jianxin Wang [1,3], Haruka Sakurai [1,3], Nobuki Kato* [1], Takuya Kaji [1], and Minoru Ueda,*[1,2]

[1] *Department of ChemistryTohoku University6-3 Aramaki-Aza Aoba, Aoba-ku, Sendai 980-8578, Japan*

[2] *Department of Molecular and Chemical Life Sciences, Graduate School of Life Sciences, Tohoku University, Sendai 980-8578, Japan*

[3]*These authors contributed equally to this work.*

Supporting Information

49 pages

Contents:

S2: Supplementary Text and Figures

S3-S9: Experimental Section

S9: Supplementary References

S10-S29: 400 MHz ^{1}H NMR spectra

S30-S49: 100 MHz ^{13}C NMR spectra

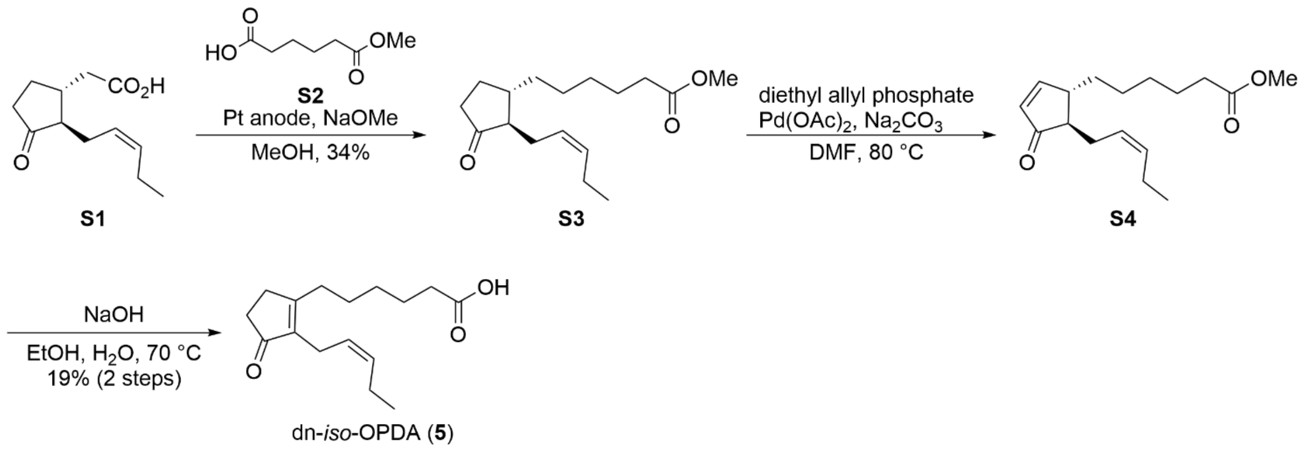

Scheme S1. Synthesis of dn-*iso*-OPDA (**5**) by Solano *et al*.

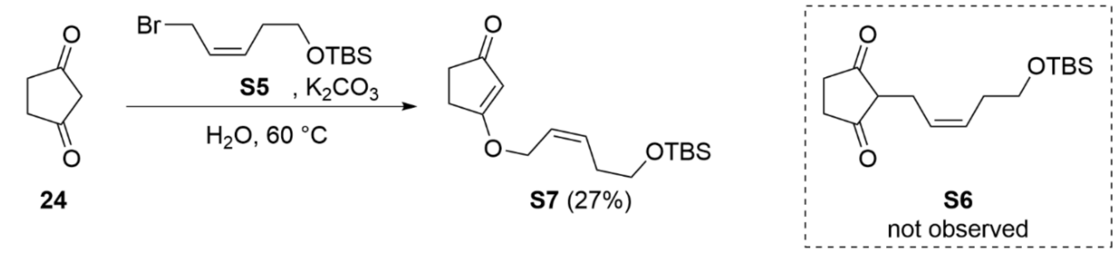

Scheme S2. Unsuccessful side chain introduction by allylation.

Experimental Section

General

All chemical reagents and solvents were obtained from commercial suppliers (Kanto Chemical Co. Ltd., Wako Pure Chemical Industries Co. Ltd., Nacalai Tesque Co. Ltd., Tokyo Chemical Industry Co. Ltd., Sigma-Aldrich Co. LLC., GE Healthcare) and used without further purification. All anhydrous solvents were either dried by standard techniques and freshly distilled before use, or purchased in anhydrous form and used as supplied. Reversed-phase high–performance liquid chromatography (HPLC) was carried out on a PU–4180 plus pump equipped with UV–4075 and MD–4010 detectors (JASCO, Tokyo, Japan). ^{1}H and ^{13}C NMR spectra were recorded on a JNM–ECS–400 spectrometer (JEOL, Tokyo, Japan) in deuterated chloroform using TMS as an internal standard. Fourier transform infrared (FT/IR) spectra were recorded on an FT/IR–4100 (JASCO, Tokyo, Japan). High–resolution (HR) electrospray ionization (ESI)–mass spectrometry (MS) analyses were conducted using a microTOF II (Bruker Daltonics Inc., Billerica, MA). Optical rotations were measured using a JASCO P–2200 polarimeter (JASCO, Tokyo, Japan). Flash chromatography was performed on an Isolera system (Biotage Ltd., North Carolina, US). TLC analyses were performed on Silica gel F254 (0.25 mm or 0.5 mm, MERCK, Germany) or RP–18F254S (0.25 mm, MERCK). All reactions were carried out under air unless stated otherwise.

Synthesis of dimethylamide (17). To a suspension of CuCN (65.5 mg, 731 μmol) in THF (14 mL) was added $TBDPSO(CH_2)_6MgCl$ (27 mL, 0.52 M in THF, 14.1 mmol) at -30 °C under an argon atmosphere. The reaction mixture was stirred at -30 °C for 20 min, and a solution of **15** (670 mg, 4.72 mmol) in THF (7 mL) was added. After being stirred at -18 °C for 11 h, the reaction mixture was quenched with saturated aqueous NH_4Cl, and then extracted with EtOAc/*n*-hexane (2/8). The resulting organic layer was washed with saturated aqueous NaCl, dried over Na_2SO_4, and filtered. After evaporation, the residue was purified by medium-pressure chromatography (Isolera, eluent: 95:5 *n*-hexane/EtOAc to 60:40 *n*-hexane/EtOAc) to give a colorless oil (2.67 g, **16** and by-products). To a solution of the mixture (2.67 g), PPh_3 (2.48 g, 9.44 mmol) and AcOH (550 μL, 96.1 mmol) in THF (16 mL) was added DIAD (1.9 M in toluene, 5.3 mL, 10.1 mmol) at -78 °C under an argon atmosphere. The mixture was stirred at 0 °C for 1 h and the reaction was quenched with saturated aqueous NH_4Cl. The mixture was extracted with *n*-hexane. The combined organic layers were dried over Na_2SO_4 and concentrated under reduced pressure. The residue was roughly purified by medium-pressure chromatography (Isolera, eluent: *n*-hexane to 94:6 *n*-hexane/EtOAc) to give a colorless oil (1.66 g, a desired acetate and by-products). To a solution of the mixture (1.66 g) in THF/MeOH (7/3, 50 mL) was added 1M-LiOH solution (14 mL 14.0 mmol) and the mixture was stirred for 3 h. MeOH and THF were removed under reduced pressure and the mixture was extracted with Et_2O. The combined organic layers were dried over Na_2SO_4 and concentrated under reduced pressure. The crude product (1.48 g) was used for the next reaction without further purification. The mixture (1.48 g) and $MeC(OMe)_2NMe_2$ (3.8 mL) were dissolved in xylene (55 mL). The mixture was stirred at reflux temperature, and MeOH was removed in a Dean Stark apparatus with MS4A. After 5 h, the solvent was removed under reduced pressure. The crude product was purified by medium-pressure chromatography (Isolera, eluent: 95:5 *n*-hexane/EtOAc to 60:40 *n*-hexane/EtOAc) to afford **17** (1.64 g, 50% in 2 steps) as a red oil: $[\alpha]_D^{21}$ -55.8 (*c* 1.40, $CHCl_3$). ^{1}H

NMR (400 MHz, $CDCl_3$) δ_H: 7.69–7.64 (m, 4H), 7.34–7.44 (m, 6H), 5.82 (brs, 1H), 5.73 (brs, 1H), 5.66–5.57 (m, 1H), 3.66 (t, J =6.5 Hz, 2H), 3.12–3.04 (m, 1H), 2.98 (s, 3H), 2.95 (s, 3H), 2.45–2.20 (m, 4H), 2.12 (dd, J =14.7, 10.1 Hz, 1H), 1.96 (ddq, J =15.7, 8.5, 2.2 Hz, 1H), 1.59–1.50 (m, 2H), 1.44–1.20 (m, 8H), 1.04 (s, 9H); ^{13}C NMR (100 MHz, $CDCl_3$) δ_C: 172.68, 135.61, 135.53, 134.12, 130.25, 129.44, 127.53, 63.95, 43.51, 41.32, 37.44, 37.22, 35.42, 33.14, 32.57, 30.51, 29.69, 28.72, 26.87, 25.78, 19.22; IR (neat) cm^{-1}: 3047, 2927, 1651, 1103; HRMS (ESI, positive) *m/z* $[M+Na]^+$ Calcd. for $C_{31}H_{45}NNaO_2Si$: 514.3117, Found: 514.3092.

Synthesis of diol 18. To a solution of **17** (1.58 g 3.22 mmol), KH_2PO_4 (580 mg, 4.26 mmol) and $Na_2HPO_4{\cdot}H_2O$ (136 mg, 853 µmol) in THF/H_2O (1/1, 46 mL) was added I_2 (1.63 g, 6.44 mmol) and the mixture was stirred for 14 h. The reaction was quenched with saturated aqueous $Na_2S_2O_3$ and the mixture was extracted with Et_2O. The combined organic layers were dried over Na_2SO_4 and concentrated under reduced pressure. The residue was purified by medium-pressure chromatography (Isolera, eluent: 95:5 *n*-hexane/EtOAc to 60:40 *n*-hexane/EtOAc) to give an iodolactone intermediate (1.58 g, 83%) as an orange oil. $[\alpha]_D^{20}$ +2.1 (*c* 1.00, $CHCl_3$). ^{1}H NMR (400 MHz, $CDCl_3$) δ_H: 7.68-7.63 (m, 4H), 7.44-7.34 (m, 6H), 5.26 (d, J = 6.4 Hz, 1H), 4.45 (d, J = 5.1 Hz, 1H), 3.65 (t, J = 6.6 Hz, 2H), 3.15-3.05 (m, 1H), 2.72-2.60 (m, 1H), 2.59 (dd, J = 18.8, 10.5 Hz, 1H), 2.49 (dd, J = 18.8, 3.9 Hz, 1H), 2.09 (dd, J = 14.7, 5.8 Hz, 1H), 1.64 (ddd, J = 14.7, 12.4, 5.1 Hz, 1H), 1.55 (quintet, J = 6.6 Hz, 1H), 1.42-1.18 (m, 8H), 1.05 (s, 9H); ^{13}C NMR (100 MHz, $CDCl_3$) δ_C:176.47, 135.53, 134.06, 129.48, 127.55, 92.72, 63.82, 40.38, 40.15, 38.83, 32.42, 29.96, 29.39, 28.70, 28.37, 28.19, 26.88, 25.63, 19.23; IR (neat) cm^{-1}: 3066, 2927, 1786, 1466, 1165, 1107; HRMS (ESI, positive) *m/z* $[M+Na]^+$ Calcd. for $C_{29}H_{39}INaO_3Si$: 613.1611, Found: 613.1587.

To a solution of the iodolactone intermediate (1.54 g 2.61 mmol) in THF (8 mL) was added DBU (480 µL, 3.21 mmol) and the mixture was stirred at reflux temperature for 8 h. The reaction mixture was cooled to -30 °C and $LiAlH_4$ (292 mg, 7.7 mmol) was added. After being stirred at 0 °C for 40 min, the reaction mixture was quenched with EtOAc and then a homogeneous mixture of $SiO_2/K_2CO_3/H_2O$ (10/1/3, 12.4 g) was added. The mixture was stirred for 40 min and the solids were removed by filtration and washed thoroughly with EtOAc. The filtrate was evaporated and the residue was purified by medium-pressure chromatography (Isolera, eluent: 88:12 *n*-hexane/EtOAc to EtOAc) to give **18** (1.11 g, 91%) as a pale yellow oil. $[\alpha]_D^{19}$ +34.5 (*c* 1.03, $CHCl_3$). ^{1}H NMR (400 MHz, $CDCl_3$) δ_H: 7.68-7.63 (m, 4H), 7.44-7.33 (m, 6H), 6.19 (dd, J = 5.8, 2.7 Hz, 1H), 5.96 (brd, J = 5.8 Hz, 1H), 4.60 (brd, J = 5.4 Hz, 1H), 3.89 (dt, J = 9.5, 5.0 Hz, 1H), 3.76 (td, J = 9.5, 4.1 Hz, 1H), 3.64 (t, J = 6.4 Hz, 2H), 2.50-2.42 (m, 1H), 2.10 (dq, J = 11.1, 7.4 Hz, 1H), 2.03-1.84 (m, 3H), 1.69 (dq, J = 14.1, 4.7 Hz, 1H), 1.55 (quintet, J = 7.4 Hz, 2H), 1.42-1.15 (m, 6H), 1.04 (s, 9H); ^{13}C NMR (100 MHz, $CDCl_3$) δ_C: 141.11, 135.53, 134.12, 131.77, 129.45, 127.53, 76.38, 63.94, 62.84, 46.79, 44.58, 33.53, 32.55, 29.63, 28.04, 28.01, 26.87, 25.74, 19.23; IR (neat) cm^{-1}: 3336, 2931, 2858, 1107, 702; HRMS (ESI, positive) *m/z* $[M+Na]^+$ Calcd. for $C_{29}H_{42}NaO_3Si$: 489.2801, Found: 489.2789.

Synthesis of bis-TES ether 19. To a solution of **18** (773 mg, 1.66 mmol) and imidazole (451 mg, 247 mmol) in DMF (10 mL) was added TESCl (832 µL, 4.97 mmol). The mixture was stirred for 13 h and the

reaction was quenched with saturated aqueous $NaHCO_3$. The mixture was extracted with *n*-hexane. The combined organic layers were dried over Na_2SO_4 and concentrated under reduced pressure. The residue was purified by medium-pressure chromatography (Isolera, eluent: *n*-hexane to 98:2 *n*-hexane/EtOAc) to give **19** (1.06 g, 92%) as a colorless oil. $[\alpha]_D^{20}$ -1.3 (*c* 1.18, $CHCl_3$). ^1H NMR (400 MHz, $CDCl_3$) δ_H: 7.66 (dd, *J* = 7.7, 1.5 Hz, 4H), 7.44-7.33 (m, 6H), 6.10 (dd, *J* = 5.8, 2.7 Hz, 1H), 5.82 (brd, *J* = 5.8 Hz, 1H), 4.47 (dd, *J* = 5.9, 2.3 Hz, 1H), 3.74-3.60 (m, 4H), 2.35 (brs, 1H), 2.07 (quintet, *J* = 7.0 Hz, 1H), 1.79 (dq, *J* = 13.8, 7.0 Hz, 1H), 1.64 (dq, *J* = 13.8, 7.0 Hz, 1H), 1.59-1.41 (m, 3H), 1.40-1.12 (m, 7H), 1.04 (s, 9H), 096 (t, *J* = 7.8 Hz, 9H), 0.93 (t, *J* = 7.8 Hz, 9H), 060 (q, *J* = 7.8 Hz, 6H), 0.56 (q, *J* = 7.8 Hz, 6H); ^{13}C NMR (100 MHz, $CDCl_3$) δ_C: 140.21, 135.54, 134.17, 132.58, 129.43, 127.52, 76.18, 64.00, 62.04, 46.09, 42.79, 32.61, 32.52, 29.79, 28.79, 28.01, 26.87, 25.81, 19.22, 6.95, 6.84, 5.24, 4.45; IR (neat) cm^{-1}: 2951, 2877, 1103, 737; HRMS (ESI, positive) *m/z* $[M+Na]^+$ Calcd. for $C_{41}H_{70}NaO_3Si_3$: 717.4530, Found: 717.4511.

Synthesis of diene 20. To a solution of DMSO (210 μL, 2.96 mmol) in CH_2Cl_2 (2.2 mL) was added oxalyl chloride (120 μL, 1.40 mmol) at -78 °C under an argon atmosphere. After the reaction mixture was stirred at -78 °C for 15 min, a solution of **19** (187 mg, 269 μmol) in CH_2Cl_2 (2.5 mL) was slowly added. After the reaction mixture was stirred at -65 °C for 1 h, Et_3N (420 μL, 3.01 mmol) was slowly added. The mixture was gradually warmed to room temperature for 30 min with stirring. The reaction mixture was quenched with saturated aqueous NH_4Cl. The mixture was extracted with *n*-hexane. The organic layer was washed with saturated aqueous NaCl, dried over Na_2SO_4, and filtered. The reaction mixture was concentrated under reduced pressure to afford **11** (192 mg, mixture). The crude product was used for the next reaction without further purification. To a suspension of $[Ph_3P(CH_2)_2Me]^+Br^-$ (356 mg, 924 μmol) in THF (3.6 mL) was added NaHMDS (1.0 M in THF, 450 μL, 450 μmol). The mixture was stirred for 40 min and cooled to -78 °C. To this solution were added DMF (540 μL) and a solution of **11** (192 mg, mixture) in THF (3.3 mL). The reaction mixture was gradually warmed to room temperature for 2 h. Then, the reaction was quenched with saturated aqueous NH_4Cl and extracted with *n*-hexane. The combined organic layers were washed with saturated aqueous NaCl, dried over Na_2SO_4 and concentrated under reduced pressure. The residue was purified by medium-pressure chromatography (Isolera, eluent: *n*-hexane to 4:96 *n*-hexane/EtOAc) to give **20** (116 mg, 71% in 2 steps) as a colorless oil. $[\alpha]_D^{23}$ +1.6 (*c* 1.13, $CHCl_3$). ^1H NMR (400 MHz, $CDCl_3$) δ_H: 7.69-7.63 (m, 4H), 7.44-7.31 (m, 6H), 6.10 (dd, *J* = 5.9, 2.7 Hz, 1H), 5.83 (brd, *J* = 5.9 Hz, 1H), 5.48-5.30 (m, 2H), 4.49 (dd, *J* = 5.8, 2.3 Hz, 1H), 3.64 (t, *J* = 6.5 Hz, 2H), 2.38 (brs, 1H), 2.19 (t, *J* = 7.1 Hz, 1H), 2.08 (quintet, *J* = 7.1 Hz, 1H), 1.96 (quintet, *J* = 7.1 Hz, 1H), 1.60-1.45 (m, 5H), 1.40-1.14 (m, 7H), 1.04 (s, 9H), 0.97 (t, *J* = 7.5 Hz, 3H), 0.94 (t, *J* = 7.9 Hz, 9H), 0.57 (q, *J* = 7.9 Hz, 6H); ^{13}C NMR (100 MHz, $CDCl_3$) δ_C: 140.13, 135.54, 134.15, 132.67, 131.61, 129.43, 128.71, 127.53, 76.27, 63.99, 47.22, 46.03, 32.61, 32.36, 29.79, 28.04, 26.87, 25.82, 23.24, 20.79, 19.22, 14.29, 6.96, 5.22; IR (neat) cm^{-1}: 2931, 2877, 1107, 1011, 737, 706; HRMS (ESI, positive) *m/z* $[M+Na]^+$ Calcd. for $C_{38}H_{60}NaO_2Si_2$: 627.4030, Found: 627.4004.

Synthesis of diene 21. To a solution of DMSO (514 μL, 7.25 mmol) in CH_2Cl_2 (8.0 mL) was added oxalyl chloride (310 μL, 3.62 mmol) at -78 °C under an argon atmosphere. After the reaction mixture was stirred at -78 °C for 15 min, a solution of **19** (504 mg, 725 μmol) in CH_2Cl_2 (8.0 mL) was slowly added. After the

reaction mixture was stirred at -65 °C for 40 min, Et_3N (1.11 mL, 7.97 mmol) was slowly added. The mixture was gradually warmed to room temperature for 1 h with stirring. The reaction mixture was quenched with saturated aqueous NH_4Cl. The mixture was extracted with *n*-hexane. The organic layer was washed with saturated aqueous NaCl, dried over Na_2SO_4, and filtered. The reaction mixture was concentrated under reduced pressure to afford **11** (446 mg, mixture). The crude product was used for the next reaction without further purification. To a suspension of $[Ph_3P(CH_2)_3OTHP]^+Br^-$ (1.06 g, 2.18 mmol) in THF (12 mL) was added NaHMDS (1.0 M in THF, 1.14 mL, 1.14 mmol). The mixture was stirred for 40 min and cooled to -78 °C. To this solution were added DMF (1.32 mL) and a solution of **11** (446 mg, mixture) in THF (12 mL). The reaction mixture was gradually warmed to room temperature for 2 h. Then, the reaction was quenched with saturated aqueous NH_4Cl and extracted with *n*-hexane. The combined organic layers were washed with saturated aqueous NaCl, dried over Na_2SO_4 and concentrated under reduced pressure. The residue was purified by medium-pressure chromatography (Isolera, eluent: 99:1 *n*-hexane/EtOAc to 92:8 *n*-hexane/EtOAc) to give **21** (391 mg, 76% in 2 steps) as a paleyellow oil. $[\alpha]_D^{20}$ +34.8 (*c* 1.95, $CHCl_3$). ^1H NMR (400 MHz, $CDCl_3$) δ_H: 7.71-7.61 (m, 4H), 7.44-7.33 (m, 6H), 6.10 (dd, *J* = 5.8, 2.9 Hz, 1H), 5.83 (dq, *J* = 5.8, 1.4 Hz, 1H), 5.58 (brdt, *J* = 10.8, 7.1 Hz, 1H), 5.40 (brdt, *J* = 10.8, 7.1 Hz, 1H), 4.60 (dd, *J* = 4.2, 2.9 Hz, 1H), 4.49 (dd, *J* = 5.8, 2.4 Hz, 1H), 3.87 (ddd, *J* = 10.8, 7.3, 2.9 Hz, 1H), 3.75 (dt, *J* = 9.4, 7.3 Hz, 1H), 3.64 (t, *J* = 6.6 Hz, 2H), 3.54-3.45 (m, 1H), 3.42 (dt, *J* = 9.4, 7.1 Hz, 1H), 2.45-2.34 (m, 3H), 2.29-2.15 (m, 2H), 1.98 (quintet, *J* = 7.1 Hz, 1H), 1.89-1.76 (m, 1H), 1.75-1.66 (m, 1H), 1.66-1.43 (m, 6H), 1.42-1.14 (m, 8H), 1.04 (s, 9H), 0.94 (t, *J* = 7.9 Hz, 9H), 0.57 (q, *J* = 7.9 Hz, 6H); ^{13}C NMR (100 MHz, $CDCl_3$) δ_C: 140.10, 135.53, 134.14, 132.65, 131.34, 129.42, 127.52, 125.62, 98.70, 76.16, 67.07, 63.99, 62.24, 47.11, 46.02, 32.60, 32.43, 30.72, 29.79, 28.18, 28.04, 26.86, 25.83, 25.50, 23.44, 19.59, 19.21, 6.96, 5.20; IR (neat) cm^{-1}: 2935, 2873, 1107; HRMS (ESI, positive) *m/z* $[M+Na]^+$ Calcd. for $C_{43}H_{68}NaO_4Si_2$: 727.4544, Found: 727.4560.

Synthesis of diol 22. To a solution of **21** (201 mg, 285 μmol) in THF (10 mL) was added 1 M TBAF in THF (1.42 mL, 1.42 mmol). After being stirred at room temperature for 14 h, the solvent was removed under reduced pressure. The reaction mixture was quenched with saturated aqueous NH_4Cl. The mixture was extracted with EtOAc. The organic layer was washed with saturated aqueous NaCl, dried over Na_2SO_4, and filtered.The residue was purified by medium-pressure chromatography (Isolera, eluent: 88:12 n-hexane/EtOAc to EtOAc) to give **22** (97.9 mg, 72%) as a colorless oil. . $[\alpha]_D^{20}$ +127.1 (*c* 0.52, $CHCl_3$). ^1H NMR (400 MHz, $CDCl_3$) δ_H: 7.71 (dd, *J* = 5.8, 2.7 Hz, 1H), 6.18 (dd, *J* = 5.8, 1.7 Hz, 1H), 5.59 (dtt, *J* = 9.7, 7.2, 1.4 Hz, 1H), 5.45 (dtt, *J* = 9.7, 7.3, 1.7 Hz, 1H), 3.68 (t, *J* = 6.5 Hz, 2H), 3.04-2.95 (m, 1H), 2.56-2.44 (m, 2H), 2.43-2.27 (m, 4H), 2.27-2.14 (m, 1H), 1.79-1.56 (m, 3H), 1.51-1.29 (m, 4H), 1.28-1.16 (m, 1H); ^{13}C NMR (100 MHz, $CDCl_3$) δ_C: 211.01, 178.48, 167.04, 132.54, 130.66, 126.75, 62.01, 49.44, 44.30, 33.77, 30.94, 30.49, 29.10, 27.22, 24.39, 24.18; IR (film) cm^{-1}: 3433, 2935, 1705; ^{13}C NMR (100 MHz, $CDCl_3$) δ_C: 141.15, 141.13, 132.06, 132.00, 130.91, 130.75, 127.16, 127.09, 99.17, 98.76, 75.49, 75.45, 67.26, 66.88, 62.93, 62.21, 62.10, 46.46, 46.41, 45.90, 45.88, 33.47, 33.42, 32.73, 30.38, 30.27, 29.68, 28.04, 25.68, 25.32, 25.29, 23.57, 23.49, 19.35 (a diastereomeric mixture derived from the THP group); IR (neat) cm^{-1}: 3409, 2931, 1030; HRMS (ESI, positive) *m/z* $[M+Na]^+$ Calcd. for $C_{21}H_{36}NaO_4$: 375.2511, Found: 375.2503.

Synthesis of triol 23. To a solution of **22** (75.0 mg, 213 μmol) in MeOH (20 mL) was added PPTS (18.0 mg, 717 μmol). After being stirred at 35 °C for 2 h, the solvent was removed under reduced pressure. The residue was purified by medium-pressure chromatography (Isolera, eluent: 99:1 $CHCl_3$/MeOH to 90:10 $CHCl_3$/MeOH) to give **23** (30.3 mg, 54%) as a colorless oil. $[\alpha]_D^{21}$ +72.0 (*c* 0.50, $CHCl_3$). ^1H NMR (400 MHz, $CDCl_3$) δ_H: 6.21 (dd, *J* = 5.8, 2.7 Hz, 1H), 5.93 (brd, *J* = 5.8 Hz, 1H), 5.60 (td, *J* = 10.5, 4.3 Hz, 1H), 5.42 (td, *J* = 10.5, 5.2 Hz, 1H), 4.47 (dd, *J* = 5.4, 2.7 Hz, 1H), 3.75 (dt, *J* = 10.1, 4.6 Hz, 1H), 3.65-3.55 (m, 3H), 2.88-2.37 (m, 4H), 2.24-2.13 (m, 1H), 2.12-1.98 (m, 2H), 1.63-1.49 (m, 3H), 1.47-1.19 (m, 6H), 1.19-1.05 (m, 1H); ^{13}C NMR (100 MHz, $CDCl_3$) δ_C: 141.50, 132.00, 131.82, 126.89, 75.59, 62.89, 61.71, 46.53, 45.91, 33.45, 32.69, 30.59, 29.65, 28.01, 25.63, 23.53; IR (neat) cm^{-1}: 3336, 2927, 2858, 1053; HRMS (ESI, positive) *m/z* $[M+Na]^+$ Calcd. for $C_{16}H_{28}NaO_3$: 291.1936, Found: 291.1926.

Synthesis of dione 27. To a solution of K_2CO_3 (955 mg, 6.91 mmol) in water (8 mL) was slowly added 1,3-cyclopentanedione (678 mg, 6.91 mmol). The solution was heated to 60 °C and stirred as *cis*-1-bromopent-2-ene (1.03 g, 6.91 mmol) was added dropwise. After stirring for 23 h at 60 °C, the reaction mixture was allowed to cool to rt and 1M NaOH aq. was added until a pH of 12 was reached. The mixture was extracted with Et_2O. The aqueous layer was chilled, and 2M HCl aq. was slowly added until pH 1 was reached. The product was extracted with of $CHCl_3$, dried over Na_2SO_4, and filtered. After evaporation, the residue was purified by medium-pressure chromatography (Isolera, eluent: 98:2 $CHCl_3$/MeOH to 80:20 $CHCl_3$/MeOH) to give **27** (286 mg, 25%) as a white solid. ^1H NMR (400 MHz, $CDCl_3$) δ_H: 5.51 (dtt, *J* = 10.8, 6.9, 1.7 Hz, 1H), 5.44 (dtt, *J* = 10.8, 7.5, 1.4 Hz, 1H), 2.94 (d, *J* = 6.9 Hz, 2H), 2.54 (s, 4H), 2.17 (quintet, *J* = 7.5 Hz, 2H), 0.99 (t, *J* = 7.5 Hz, 3H); ^{13}C NMR (100 MHz, $CDCl_3$) δ_C: 197.41, 134.12, 125.19, 116.63, 30.46, 20.65, 19.69, 14.23; IR (neat) cm^{-1}: 2962.12, 2869.56, 1685.48, 1623.77, 1357.64, 1261.22; HRMS (ESI, positive) *m/z* $[M + Na]^+$ Calcd. for $C_{10}H_{14}NaO_2$: 189.0891, found: 189.0887.

Synthesis of cyclopentenone 28. To a solution of **27** (279 mg, 1.68 mmol) in acetone (5 mL) were added K_2CO_3 (255 mg, 1.85 mmol) and Me_2SO_4 (193 μL, 2.02 mmol). After being stirred at reflux temperature for 4 h, the reaction mixture was quenched with saturated aqueous $NaHCO_3$. Then the water layer was extracted with CH_2Cl_2. The resulting organic layer was washed with saturated aqueous NaCl, dried over Na_2SO_4, and filtered. After evaporation, the residue was purified by medium-pressure chromatography (Isolera, eluent: 99:1 $CHCl_3$/MeOH to 90:10 $CHCl_3$/MeOH) to give **28** (304 mg, quant.) as a yellow oil. ^1H NMR (400 MHz, $CDCl_3$) δ_H: 5.38 (ddd, J = 10.7, 6.4, 1.6 Hz, 1H), 5.32 (ddd, *J* = 10.7, 6.9, 1.0 Hz, 1H), 3.95 (s, 3H), 2.88 (d, *J* = 6.4 Hz, 2H), 2.67-2.64 (m, 2H), 2.46-2.43 (m, 2H), 2.15 (quintetd, *J* = 6.9, 1.0 Hz, 2H), 0.97 (t, *J* = 6.9 Hz, 3H); ^{13}C NMR (100 MHz, $CDCl_3$) δ_C: 204.52, 184.67, 132.27, 125.55, 119.80, 56.56, 33.59, 24.71, 20.57, 19.56, 14.32; IR (neat) cm^{-1}: 2962.13, 2931.27, 1550.49, 1353.78, 1257.36; HRMS (ESI, positive) *m/z* $[M + Na]^+$ Calcd. for $C_{11}H_{16}NaO_2$: 203.1048, found: 203.1037.

Synthesis of dione 31. A solution of allylpalladium (II) chloride dimer (138 mg, 377 μmol) and dppe (599 mg, 1.50 mmol) in degassed THF (37 mL) was treated successively with allyl acetate (1.6 mL, 15.0 mmol), 1,3-cyclopentadione (2.20 g, 22.5 mmol), BSA (5.5 mL, 22.5 mmol) and sodium acetate (51.5 mg, 628

μmol) at room temperature. After complete addition the reaction mixture was heated to reflux for 20 h. The solvent was removed under reduced pressure and the residue was taken up in dichloromethane (30 mL). The organic phase was washed with 1M HCl aq., the phases were separated and the aqueous phase was extracted with dichloromethane. The combined organic phases were dried with Na_2SO_4, filtered and the solvent was removed under reduced pressure. The residue was purified by medium-pressure chromatography (Isolera, eluent: 98:2 $CHCl_3$/MeOH to 80:20 $CHCl_3$/MeOH) to give **31** (1.66 g, 81%) as a pale yellow solid. All the analytical data are in the agreement with the reported data.[1-3]

Synthesis of cyclopentenone 32. To a solution of **31** (397 mg, 2.87 mmol) in acetone (8.5 mL) were added K_2CO_3 (437 mg, 3.16 mmol) and Me_2SO_4 (328 μL, 3.44 mmol). After being stirred at reflux temperature for 3 h, the reaction mixture was quenched with saturated aqueous $NaHCO_3$. Then the mixture was extracted with CH_2Cl_2. The resulting organic layer was washed with saturated aqueous NaCl, dried over Na_2SO_4, and filtered. After evaporation, the residue was purified by medium-pressure chromatography (Isolera, eluent: 99:1 $CHCl_3$/MeOH to 90:10 $CHCl_3$/MeOH) to give **32** (429 mg, 98%) as a yellow oil. All the analytical data are in the agreement with the reported data.[4]

Synthesis of alcohol 33. To a solution of THPO$(CH_2)_6$MgBr (1.0 M in THF, 7.0 mL, 7.00 mmol) was added to a solution of **32** (429 mg, 2.82 mmol) in THF (2.0 mL) at reflux temperature under an argon atmosphere. After being stirred at reflux temperature for 2 h, the reaction mixture was allowed to cool to rt and 10% HCl aq. (7 mL) was added. After 18 h of stirring at reflux temperature, H_2O was added and the water layer was extracted with EtOAc. The combined organic layers were washed with saturated aqueous NaCl, dried over Na_2SO_4 and concentrated under reduced pressure. After evaporation, the residue was purified by medium-pressure chromatography (Isolera, eluent: 88:12 *n*-hexane/EtOAc to EtOAc) to to give **33** (226 mg, 36%) as a yellow oil. ^{1}H NMR (400 MHz, $CDCl_3$) δ_H: 5.77 (ddt, *J* = 18.0, 9.4, 6.4 Hz, 1H), 4.96 (dq, *J* = 18.0, 2.0 Hz, 1H), 4.95 (dq, *J* = 9.4, 2.0 Hz, 1H), 3.65 (t, *J* = 6.4 Hz, 2H), 2.95 (d, *J* = 6.4 Hz, 2H), 2.54-2.52 (m, 2H), 2.43 (t, *J* = 7.8 Hz, 2H), 2.40-2.38 (m, 2H), 1.61-1.51 (m, 4H), 1.44-1.32 (m, 4H); ^{13}C NMR (100 MHz, $CDCl_3$) δ_C: 209.53, 175.43, 137.99, 135.06, 115.27, 62.91, 34.28, 32.68, 31.28, 29.63, 29.33, 27.45, 27.39, 25.68; IR (neat) cm^{-1}:3429, 2931, 2858, 1693, 1639; HRMS (ESI, positive) *m/z* $[M + Na]^+$ Calcd. for $C_{13}H_{20}NaO_2$: 245.1517, found: 245.1512.

Synthesis of diol 34. A 5 mL pear-shaped flask was charged with **33** (43.0 mg, 193 μmol), CH_2=$CHCH_2CH_2OAc$ (145 mg, 1.27 mmol) and Hoveyda-Grubbs Catalyst M2001 Umicore (8.5 mg, 13.4 μmol). After 6 h of stirring, the residue was roughly purified by medium-pressure chromatography (Isolera, eluent: 99:1 $CHCl_3$/MeOH to 90:10 $CHCl_3$/MeOH) to give a mixture (26.7 mg). The crude mixture was used for the next reaction without further purification. To a solution of the mixture (26.7 mg) in MeOH (420 μL) was added 0.3M-NaOH solution (1.3 mL, 390 μmol) and the mixture was stirred at 50 ° for 1.5 h. The reaction mixture was quenched with 2M HCl aq. and the aqueous layer was extracted with EtOAc. The organic layer was washed with saturated aqueous NaCl, dried over Na_2SO_4, and filtered. After evaporation, the residue was purified medium-pressure chromatography (Isolera, eluent: 0.1:99:1 AcOH/$CHCl_3$/MeOH to 0.1:86:14 AcOH/$CHCl_3$/MeOH) to give **34** (16.1 mg, 32% in 2 steps) as a colorless oil. ^{1}H NMR (400

MHz, $CDCl_3$) δ_H: 5.43 (dtt, *J* = 10.8, 7.2, 1.5 Hz, 1H), 5.37 (dtt, *J* = 10.8, 6.8, 1.3 Hz, 1H), 3.69 (t, *J* = 6.2 Hz, 2H), 3.66 (t, *J* = 6.4 Hz, 2H), 2.98 (d, *J* = 6.8 Hz, 2H), 2.53-2.51 (m, 2H), 2.49-2.43 (m, 4H), 2.38-2.36 (m, 2H), 1.61-1.52 (m, 4H), 1.46-1.34 (m, 4H); ^{13}C NMR (100 MHz, $CDCl_3$) δ_C: 210.25, 175.16, 138.82, 128.96, 127.10, 62.89, 62.20, 34.36, 32.65, 31.30, 31.01, 29.54, 29.42, 27.58, 25.65, 21.89; IR (neat) cm^{-1}: 3410, 2931, 2862, 1686, 1635; HRMS (ESI, positive) *m/z* $[M + Na]^+$ Calcd. for $C_{16}H_{26}NaO_3$: 289.1780, found: 289.1775.

Supplementary References

1. P. K. Ruprah, J.-P. Cros, J. E. Pease, W. G. Whittingham and J. M. J. Williams, *Eur. J. Org. Chem.* 2002, 3145-3152.
2. E. Lacoste, E. Vaique, M. Berlande, I. Pianet, J.-M. Vincent and Y. Landais, *Eur. J. Org. Chem.* 2007, 167-177.
3. X.-M. Zhang, M. Wang, Y.-Q. Tu, C.-A. Fan, Y.-J. Jiang, S.-Y. Zhang and F.-M. Zhang, *Synlett* 2008, 2831-2835.
4. P. K. Ruprah, J.-P. Cros, J. E. Pease, W. G. Whittingham, J. M. J. Williams, *Eur. J. Org. Chem.* 2002, 3145-3152.

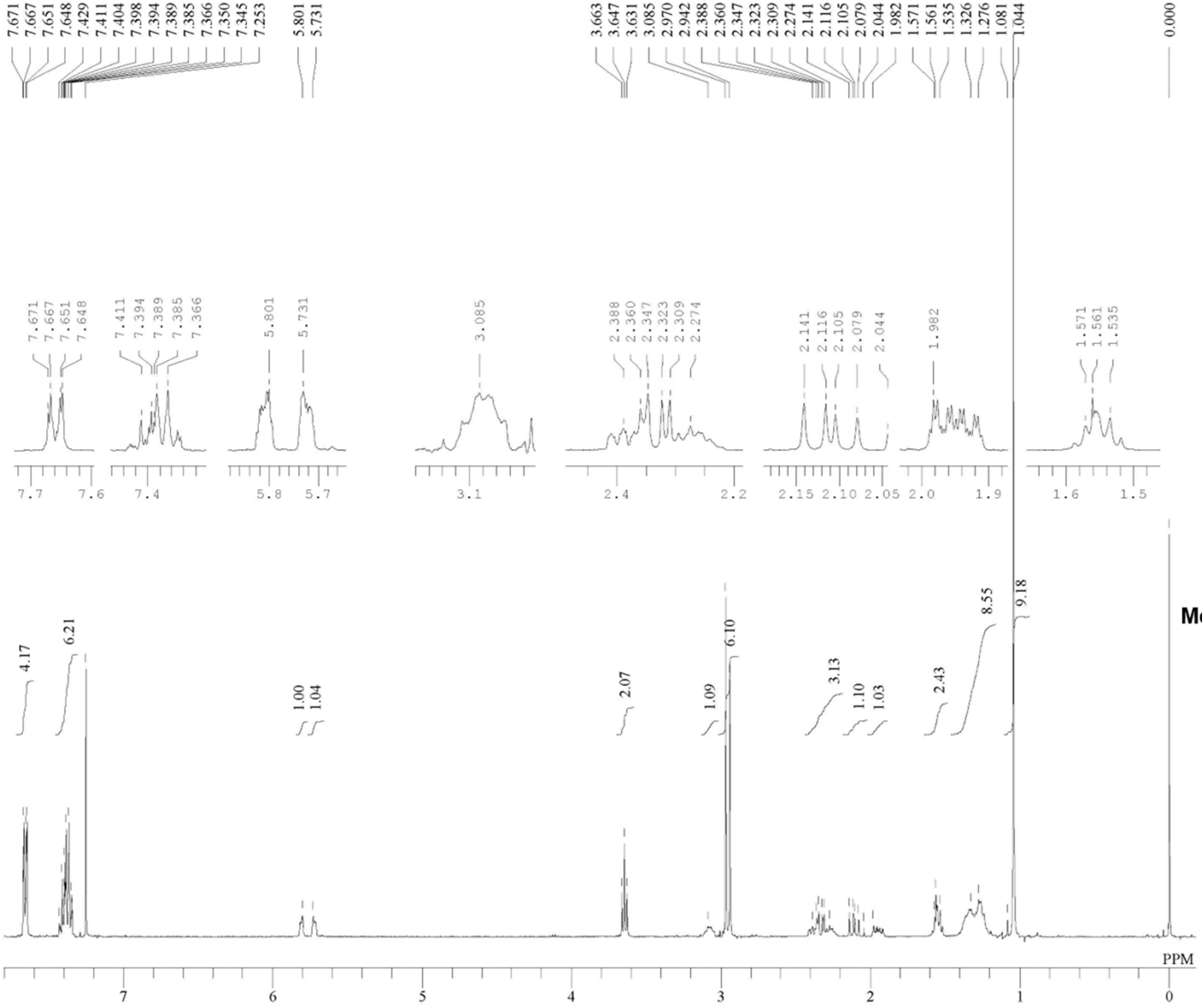

DFILE 1H_17.jdf
COMNT single_pulse
DATIM 02-04-2020 17:27:52
OBNUC 1H
EXMOD proton.jxp
OBFRQ 399.78 MHz
OBSET 4.19 KHz
OBFIN 7.29 Hz
POINT 16400
FREQU 7503.00 Hz
SCANS 8
ACQTM 2.1837 sec
PD 5.0000 sec
PW1 2.95 usec
IRNUC 1H
CTEMP 23.0 c
SLVNT CDCL3
EXREF 0.00 ppm
BF 0.25 Hz
RGAIN 54

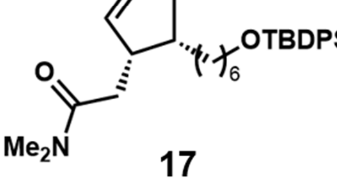

17

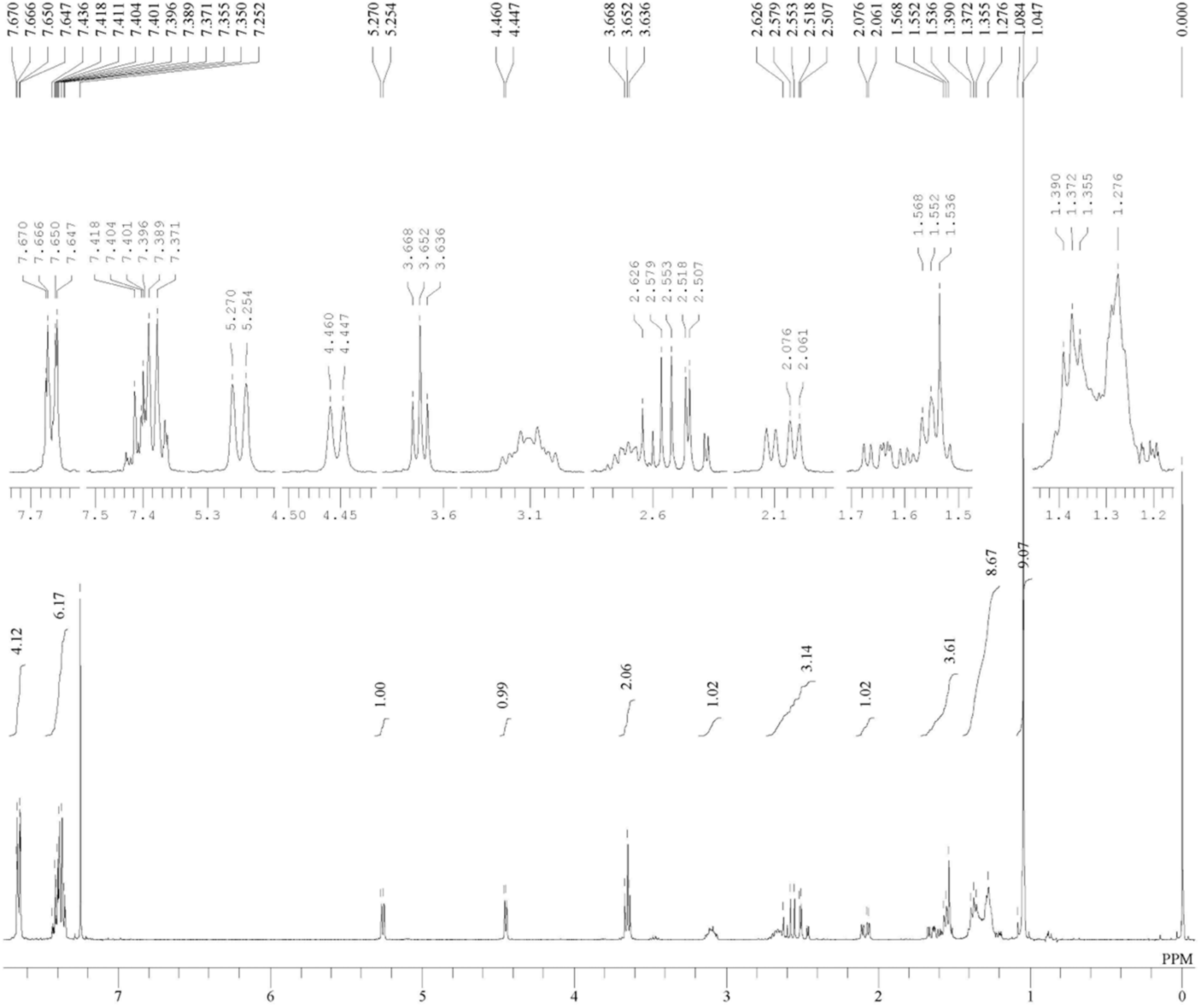

DFILE 1H_iodolactone.jdf
COMNT single_pulse
DATIM 03-04-2020 16:22:22
OBNUC 1H
EXMOD proton.jxp
OBFRQ 399.78 MHz
OBSET 4.19 KHz
OBFIN 7.29 Hz
POINT 16400
FREQU 7503.00 Hz
SCANS 8
ACQTM 2.1837 sec
PD 5.0000 sec
PW1 2.95 usec
IRNUC 1H
CTEMP 23.3 c
SLVNT CDCL3
EXREF 0.00 ppm
BF 0.25 Hz
RGAIN 56

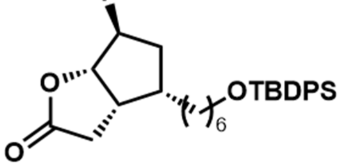

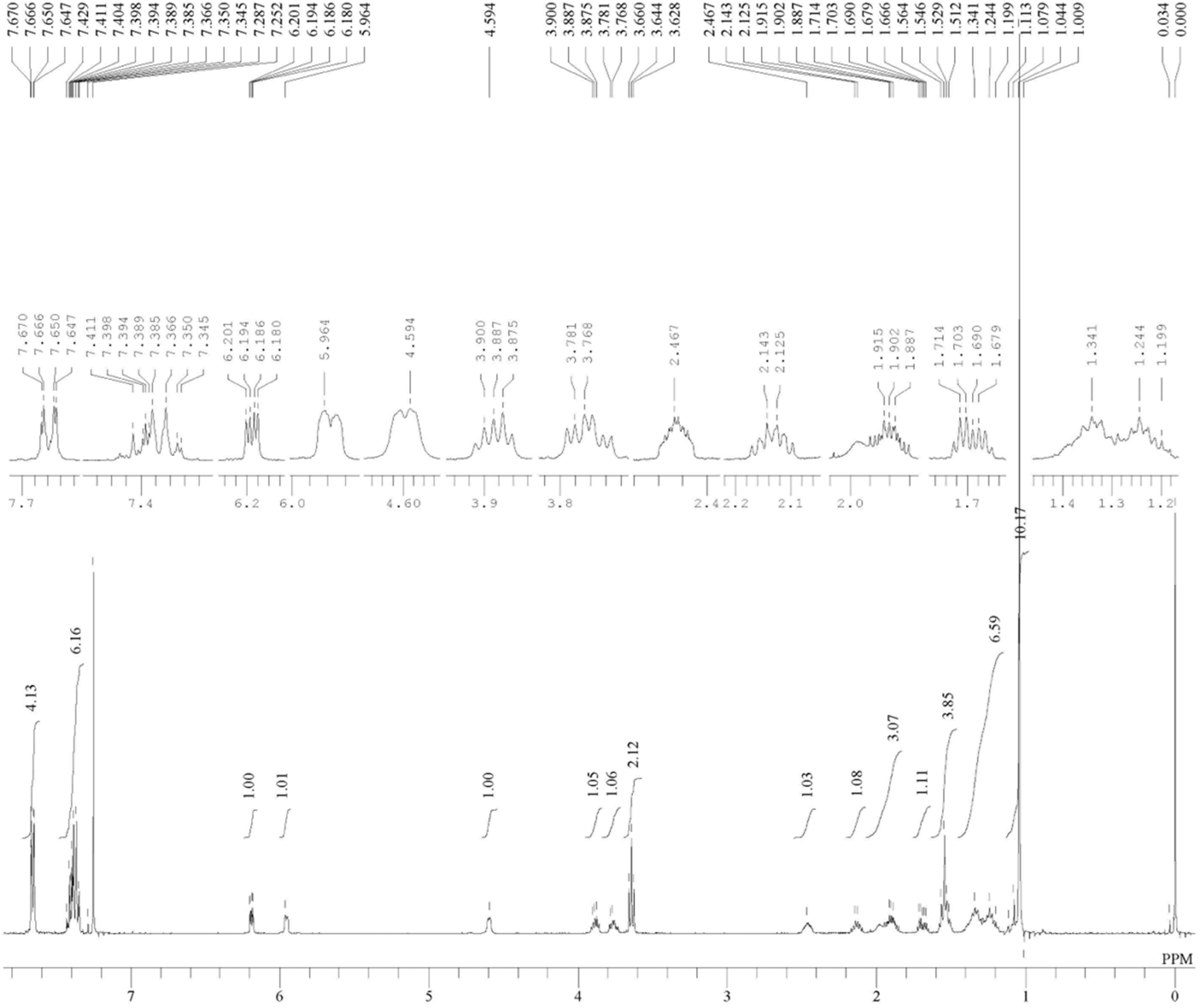
7.670 7.666 7.650 7.647 7.429 7.411 7.404 7.398 7.394 7.389 7.385 7.366 7.350 7.345 7.287 7.252 6.201 6.194 6.186 6.180 5.964
4.594
3.900 3.887 3.875 3.781 3.768 3.660 3.644 3.628
2.467 2.143 2.125 1.915 1.902 1.887 1.714 1.703 1.690 1.679 1.666 1.564 1.546 1.529 1.512 1.341 1.244 1.199 1.113 1.079 1.044 1.009
0.034 0.000
DFILE 1H_18.jdf
COMNT single_pulse
DATIM 05-04-2020 17:54:38
OBNUC 1H
EXMOD proton.jxp
OBFRQ 399.78 MHz
OBSET 4.19 KHz
OBFIN 7.29 Hz
POINT 16400
FREQU 7503.00 Hz
SCANS 8
ACQTM 2.1837 sec
PD 5.0000 sec
PW1 2.95 usec
IRNUC 1H
CTEMP 23.7 c
SLVNT CDCL3
EXREF 0.00 ppm
BF 0.25 Hz
RGAIN 56
4.13 6.16 1.00 1.01 1.00 1.05 1.06 2.12 1.03 1.08 3.07 1.11 3.85 6.59 10.17
PPM

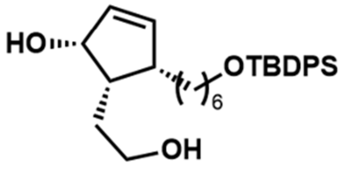
HO
OTBDPS
6
OH

18

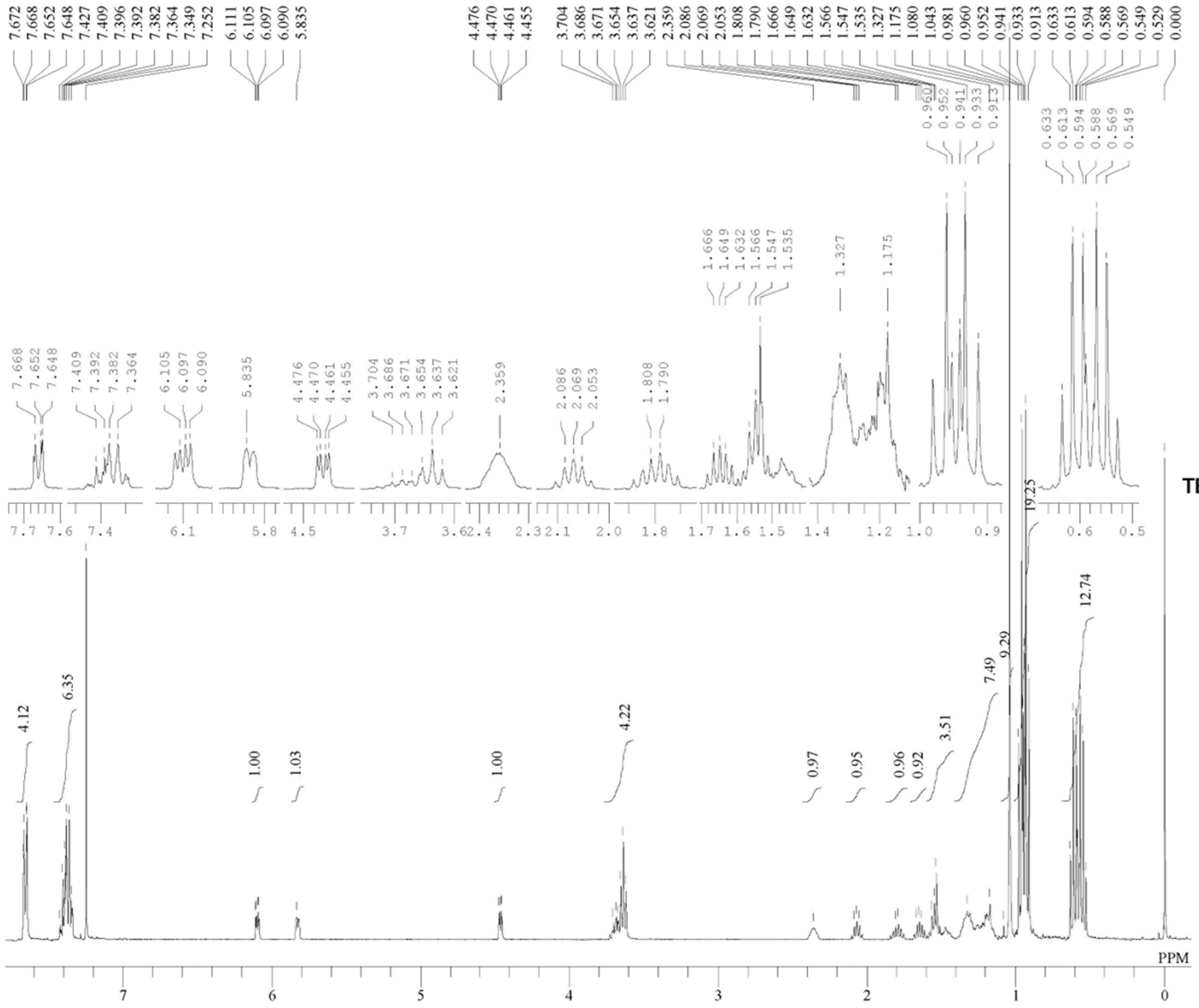
DFILE 1H_19.jdf
COMNT single_pulse
DATIM 07-04-2020 13:06:21
OBNUC 1H
EXMOD proton.jxp
OBFRQ 399.78 MHz
OBSET 4.19 KHz
OBFIN 7.29 Hz
POINT 16400
FREQU 7503.00 Hz
SCANS 8
ACQTM 2.1837 sec
PD 5.0000 sec
PW1 2.95 usec
IRNUC 1H
CTEMP 23.2 c
SLVNT CDCL3
EXREF 0.00 ppm
BF 0.25 Hz
RGAIN 52
PPM

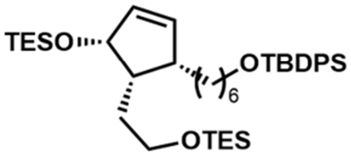
TESO
OTBDPS
6
OTES

19

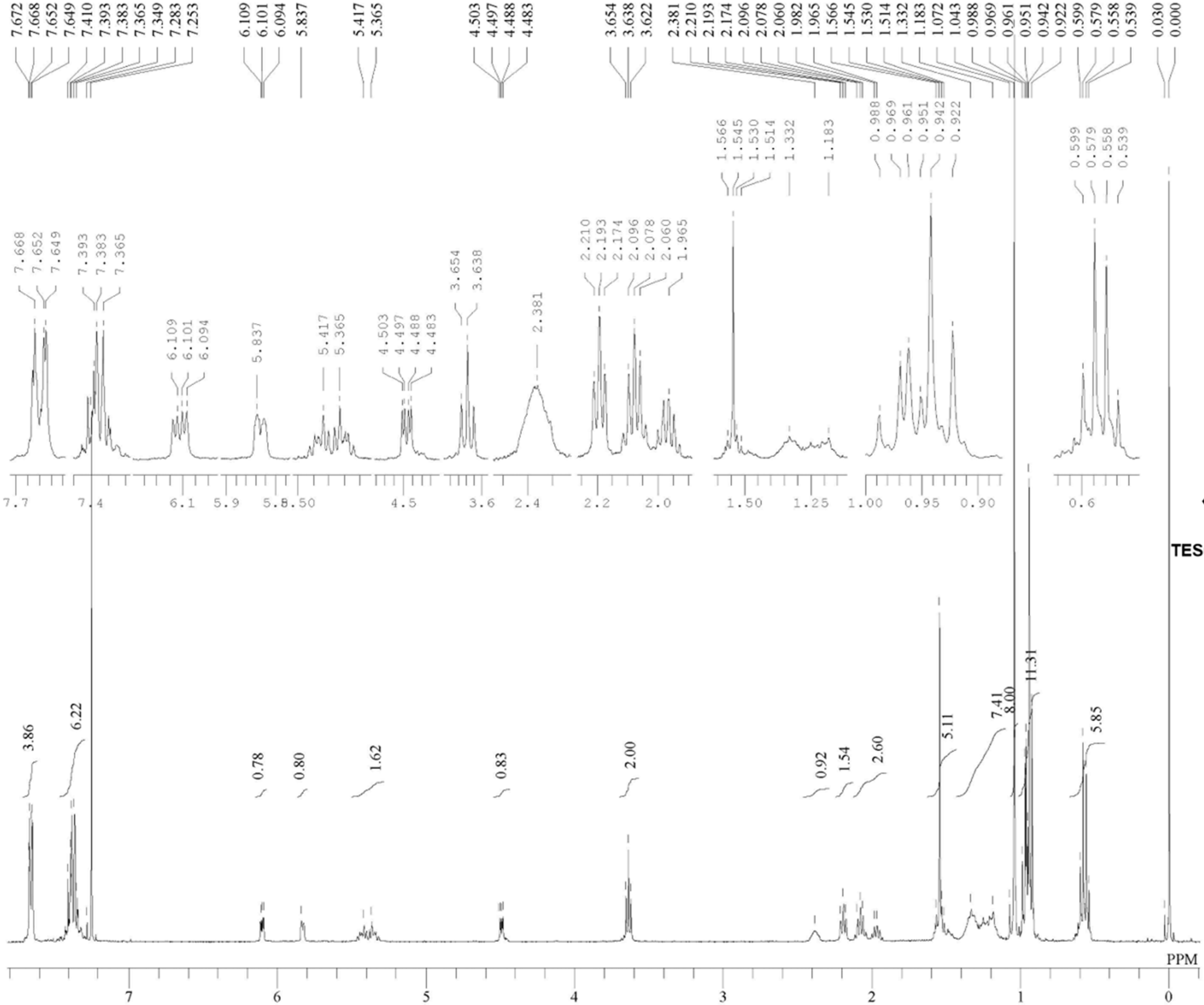
DFILE 1H_20.jdf
COMNT single_pulse
DATIM 21-08-2020 19:54:07
OBNUC 1H
EXMOD proton.jxp
OBFRQ 399.78 MHz
OBSET 4.19 KHz
OBFIN 7.29 Hz
POINT 16400
FREQU 7503.00 Hz
SCANS 8
ACQTM 2.1837 sec
PD 5.0000 sec
PW1 2.95 usec
IRNUC 1H
CTEMP 21.5 c
SLVNT CDCL3
EXREF 0.00 ppm
BF 0.25 Hz
RGAIN 56
PPM

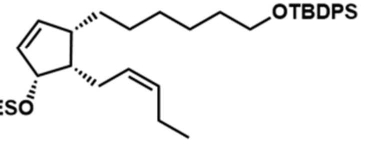
OTBDPS
TESO

20

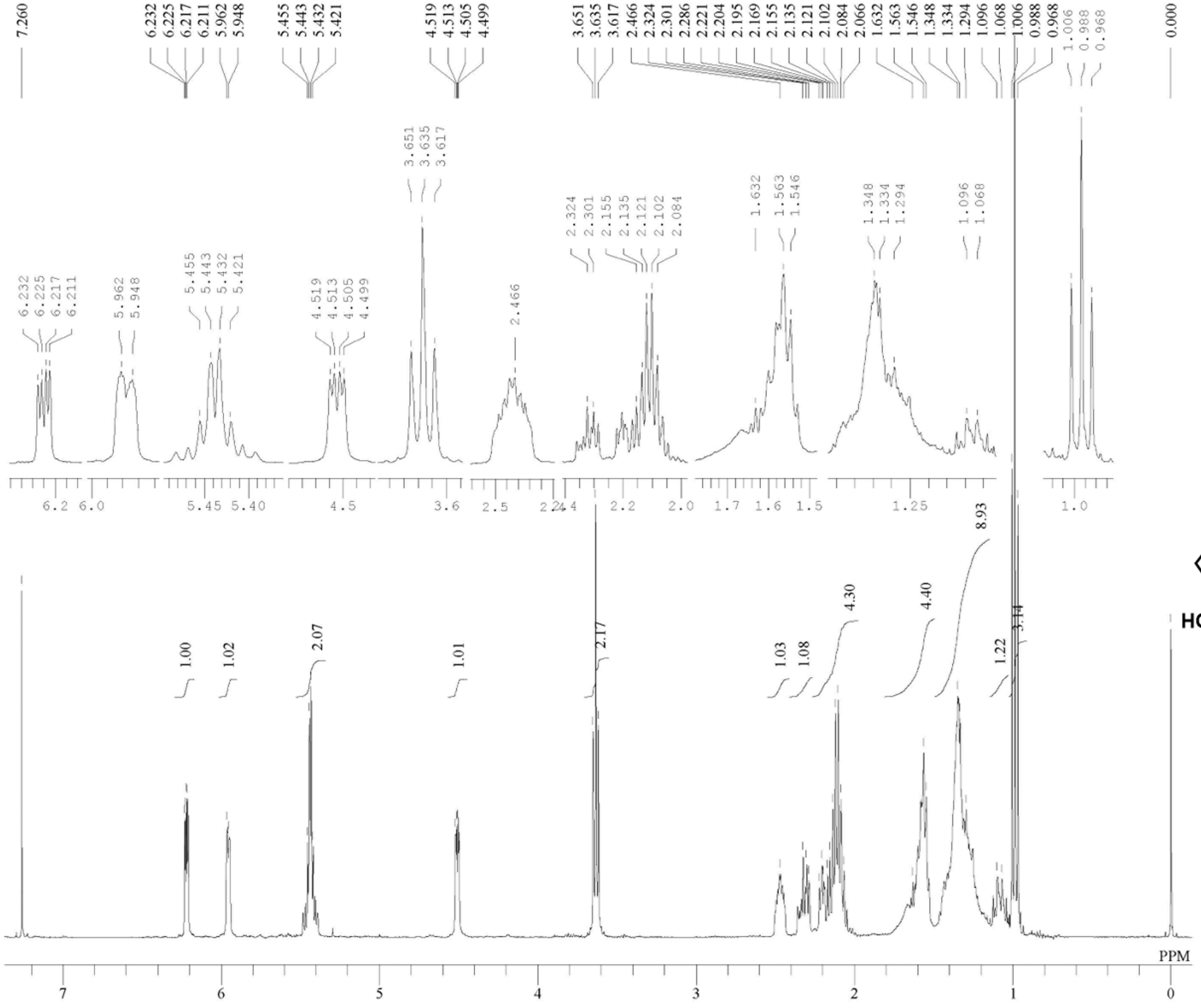

DFILE	1H_diol.jdf
COMNT	single_pulse
DATIM	23-08-2020 20:27:39
OBNUC	1H
EXMOD	proton.jxp
OBFRQ	399.78 MHz
OBSET	4.19 KHz
OBFIN	7.29 Hz
POINT	16400
FREQU	7503.00 Hz
SCANS	8
ACQTM	2.1837 sec
PD	5.0000 sec
PW1	2.95 usec
IRNUC	1H
CTEMP	21.6 c
SLVNT	CDCL3
EXREF	0.00 ppm
BF	0.25 Hz
RGAIN	46

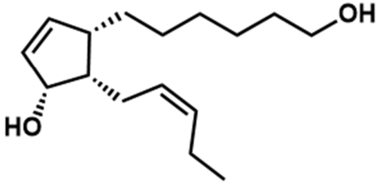

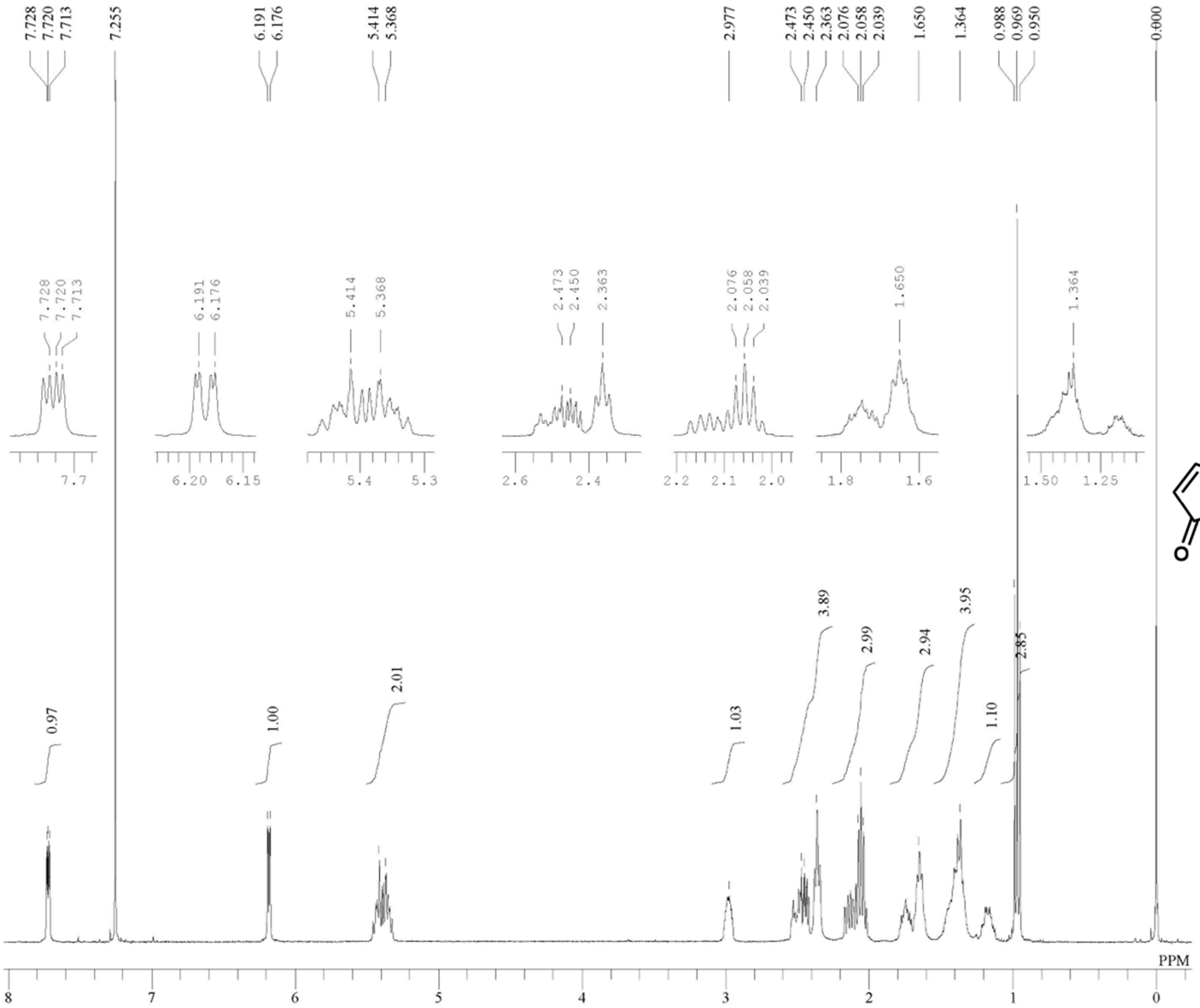
DFILE 1H_04.jdf
COMNT single_pulse
DATIM 13-04-2020 17:42:25
OBNUC 1H
EXMOD proton.jxp
OBFRQ 399.78 MHz
OBSET 4.19 KHz
OBFIN 7.29 Hz
POINT 16400
FREQU 7503.00 Hz
SCANS 8
ACQTM 2.1837 sec
PD 5.0000 sec
PW1 2.95 usec
IRNUC 1H
CTEMP 22.8 c
SLVNT CDCL3
EXREF 0.00 ppm
BF 0.25 Hz
RGAIN 56
PPM

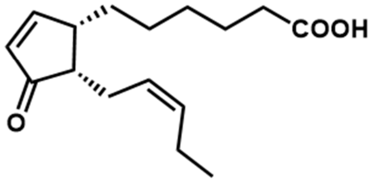
COOH
O

dn-*cis*-OPDA (**4**)

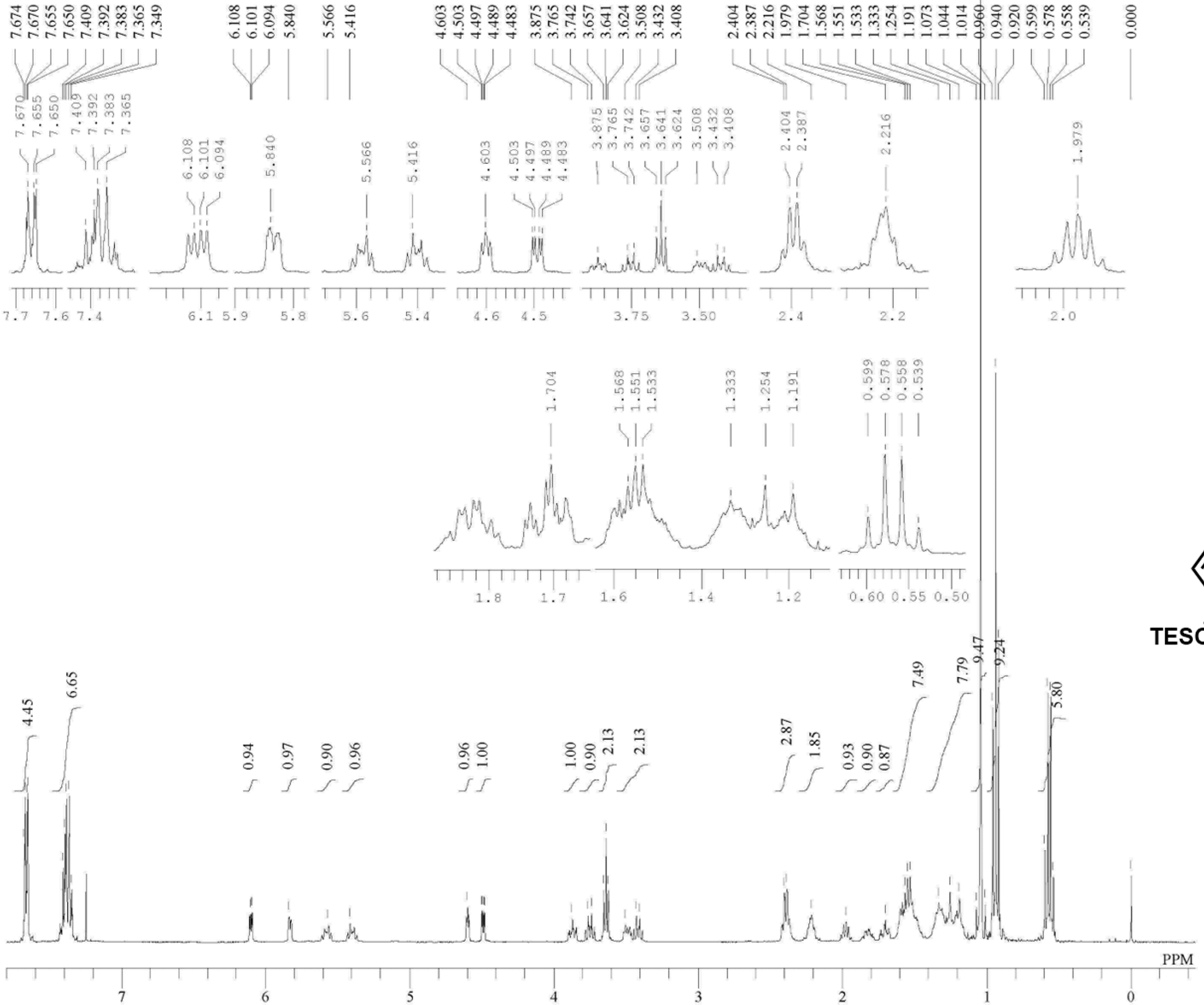

DFILE	Wittig_Proton-1-1.jdf
COMNT	single_pulse
DATIM	17-08-2020 11:09:42
OBNUC	1H
EXMOD	proton.jxp
OBFRQ	399.78 MHz
OBSET	4.19 KHz
OBFIN	7.29 Hz
POINT	16400
FREQU	7503.00 Hz
SCANS	8
ACQTM	2.1837 sec
PD	5.0000 sec
PW1	2.95 usec
IRNUC	1H
CTEMP	21.5 c
SLVNT	CDCL3
EXREF	0.00 ppm
BF	0.25 Hz
RGAIN	38

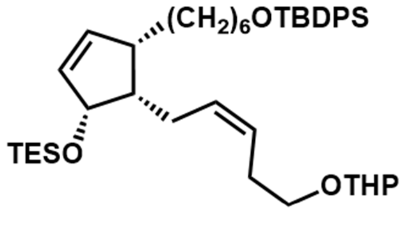

21

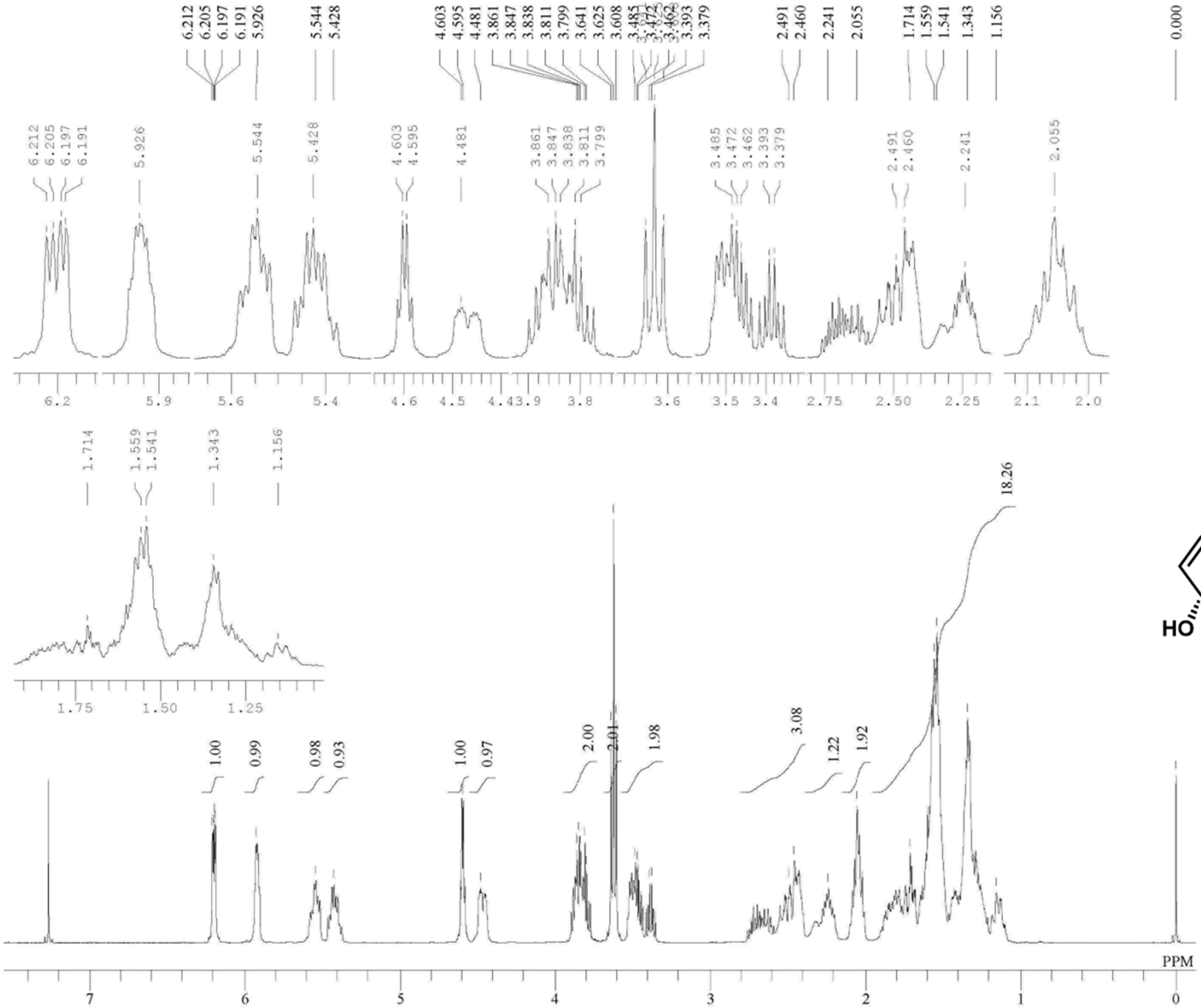
DFILE TBAF_1H-1.jdf
COMNT single_pulse
DATIM 01-09-2020 11:28:10
OBNUC 1H
EXMOD proton.jxp
OBFRQ 399.78 MHz
OBSET 4.19 KHz
OBFIN 7.29 Hz
POINT 16400
FREQU 7503.00 Hz
SCANS 8
ACQTM 2.1837 sec
PD 5.0000 sec
PW1 2.95 usec
IRNUC 1H
CTEMP 22.5 c
SLVNT CDCL3
EXREF 0.00 ppm
BF 0.25 Hz
RGAIN 40
PPM

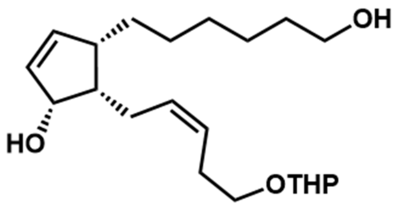
OH
HO
OTHP

22

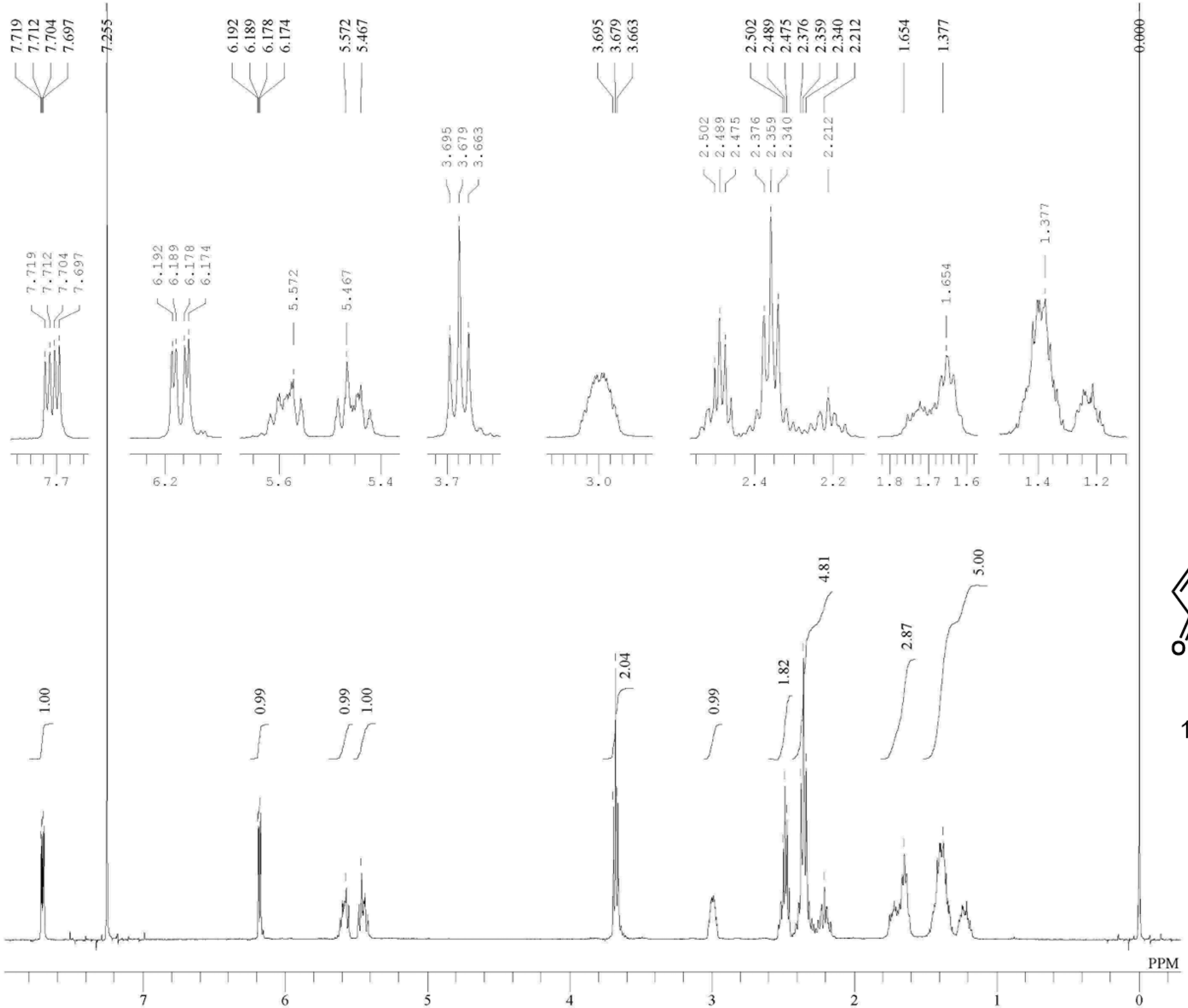

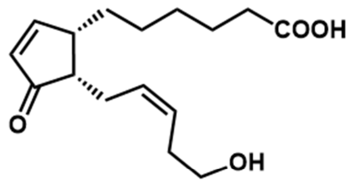

16OH-dn-*cis*-OPDA (**6**)

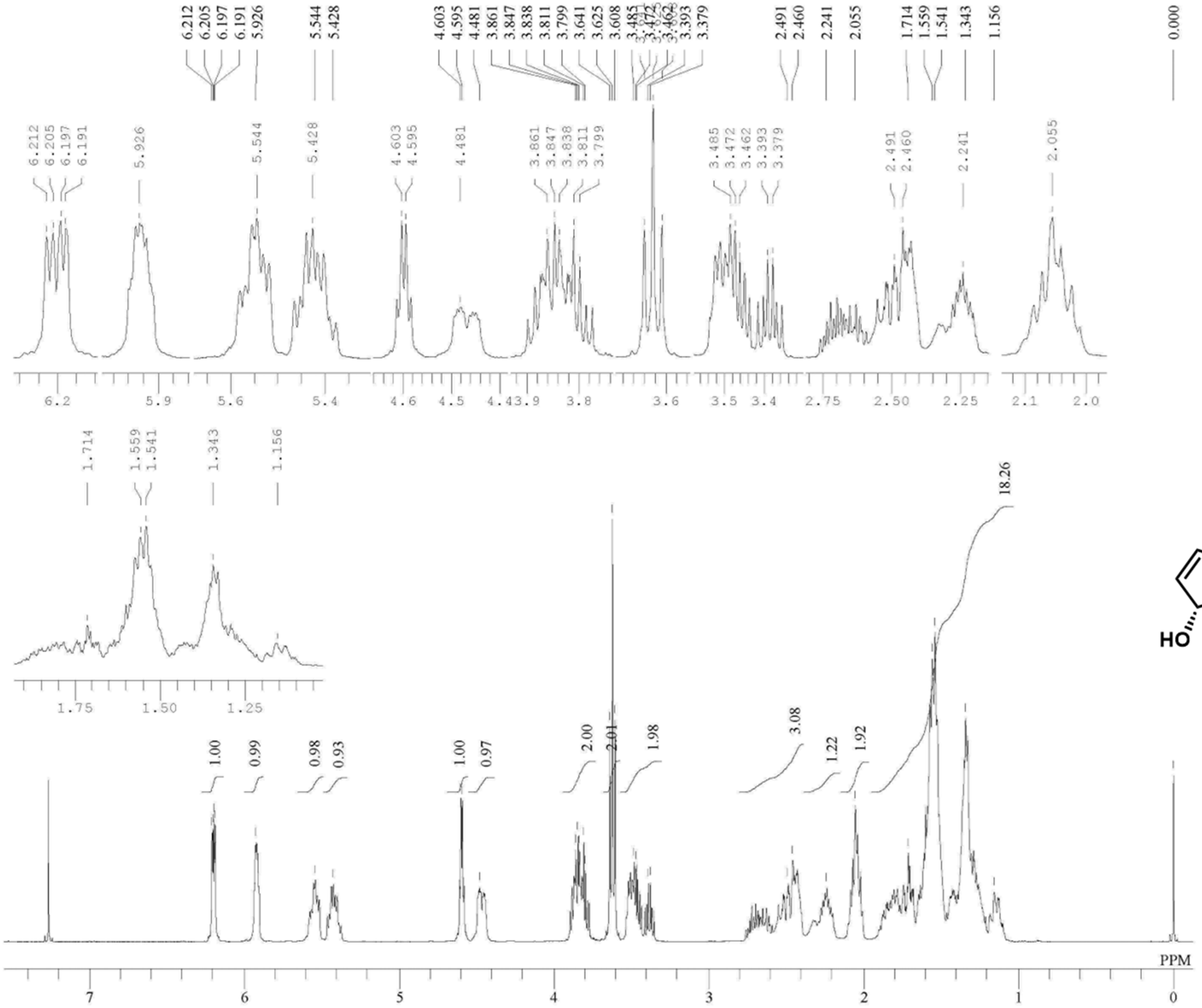

DFILE TBAF_1H-1.jdf
COMNT single_pulse
DATIM 01-09-2020 11:28:10
OBNUC 1H
EXMOD proton.jxp
OBFRQ 399.78 MHz
OBSET 4.19 KHz
OBFIN 7.29 Hz
POINT 16400
FREQU 7503.00 Hz
SCANS 8
ACQTM 2.1837 sec
PD 5.0000 sec
PW1 2.95 usec
IRNUC 1H
CTEMP 22.5 c
SLVNT CDCL3
EXREF 0.00 ppm
BF 0.25 Hz
RGAIN 40

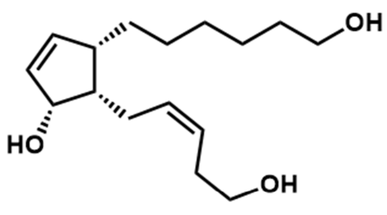

23

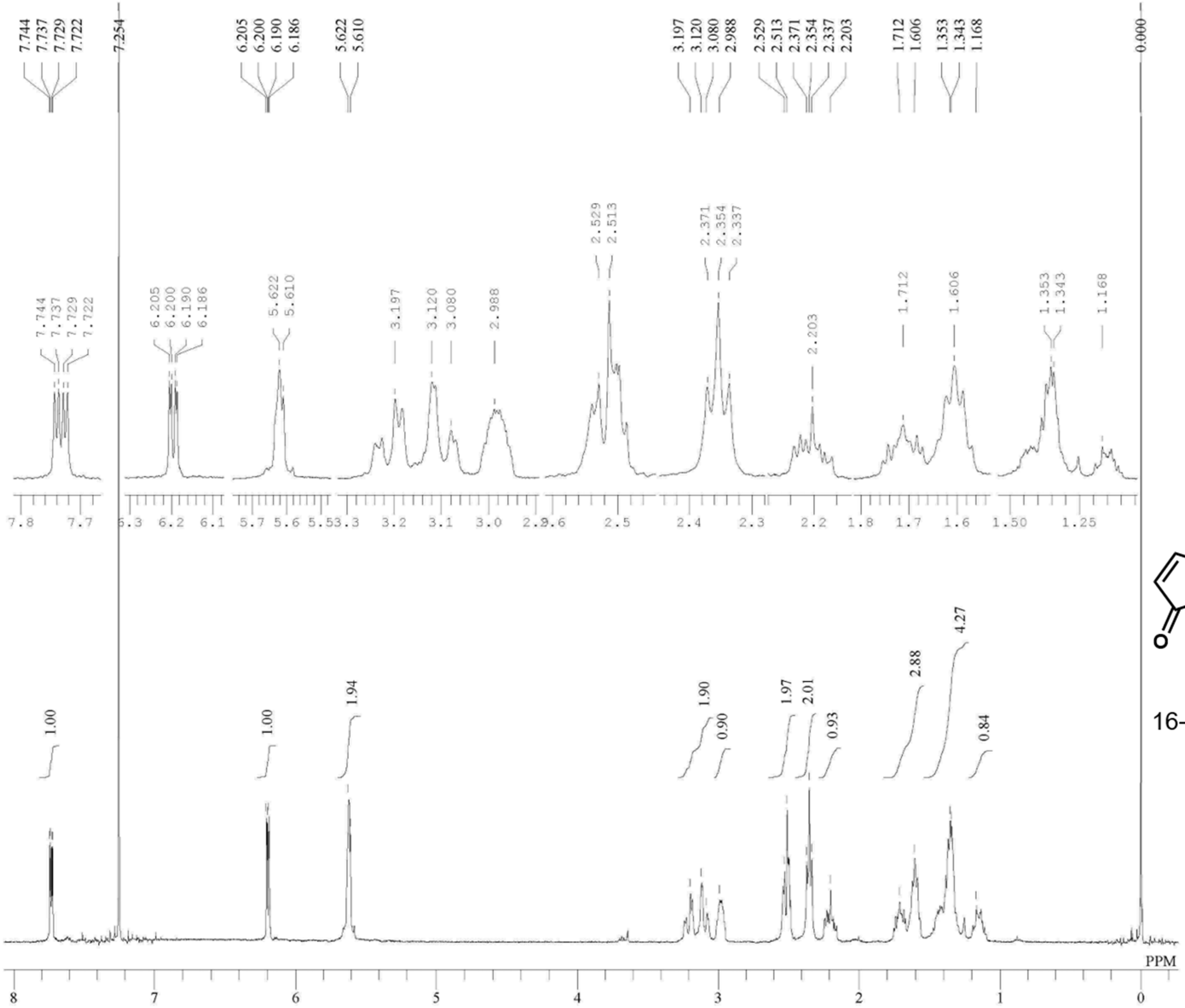

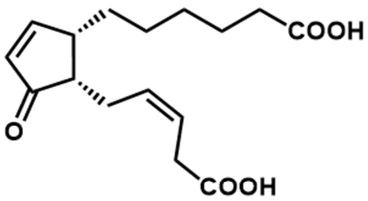

16-COOH-dn-*cis*-OPDA (**7**)

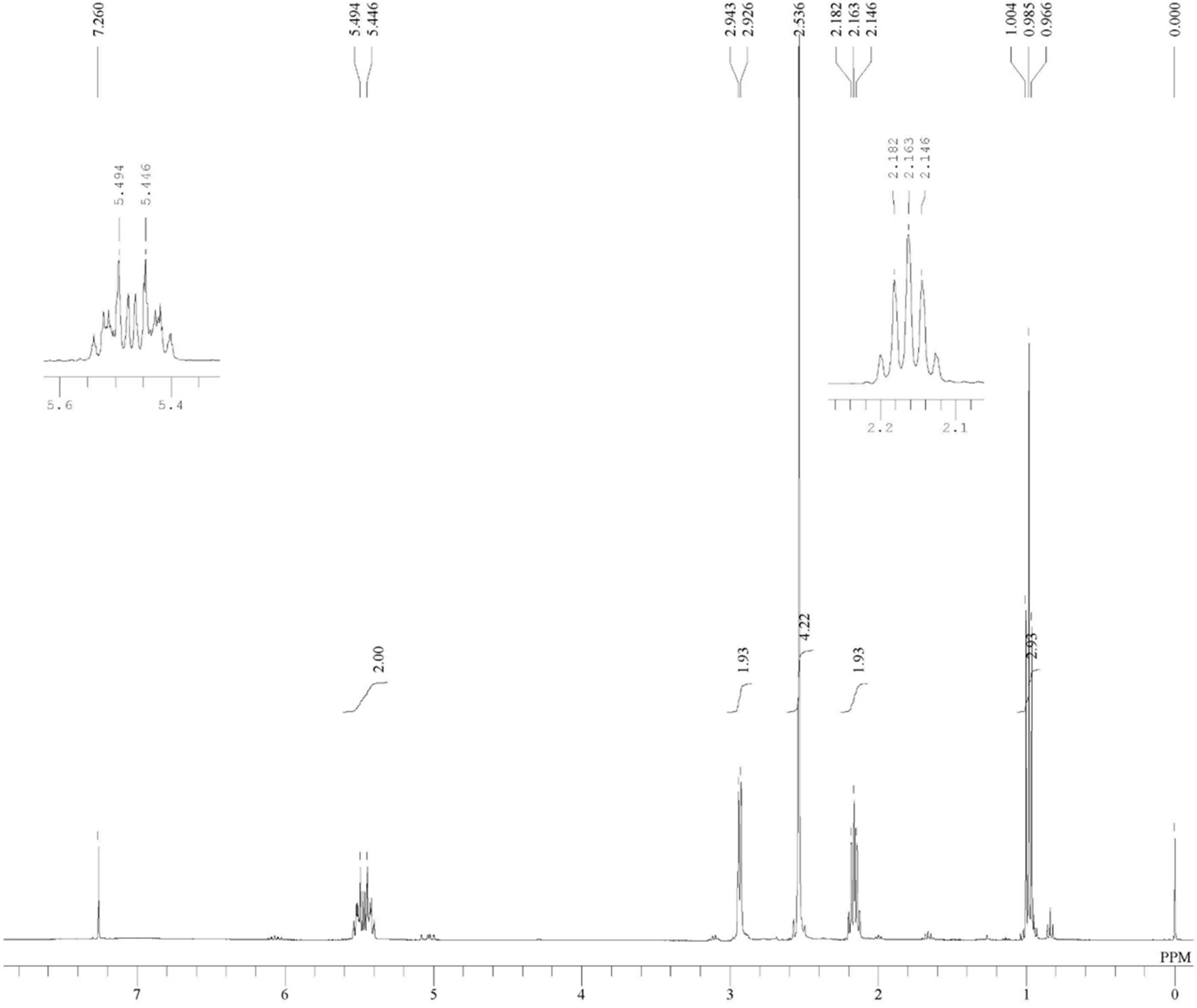
7.260
5.494
5.446
2.943
2.926
2.536
2.182
2.163
2.146
1.004
0.985
0.966
0.000
2.00
1.93
4.22
1.93
2.93
5.6
5.4
2.2
2.1
PPM
7
6
5
4
3
2
1
0
DFILE 27target_Proton-1-1.jdf
COMNT single_pulse
DATIM 19-08-2020 19:37:00
OBNUC 1H
EXMOD proton.jxp
OBFRQ 399.78 MHz
OBSET 4.19 KHz
OBFIN 7.29 Hz
POINT 16400
FREQU 7503.00 Hz
SCANS 8
ACQTM 2.1837 sec
PD 5.0000 sec
PW1 2.95 usec
IRNUC 1H
CTEMP 21.6 c
SLVNT CDCL3
EXREF 0.00 ppm
BF 0.25 Hz
RGAIN 46

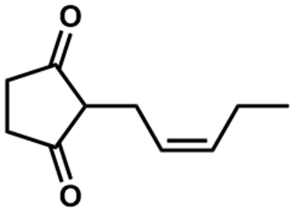
O
O

27

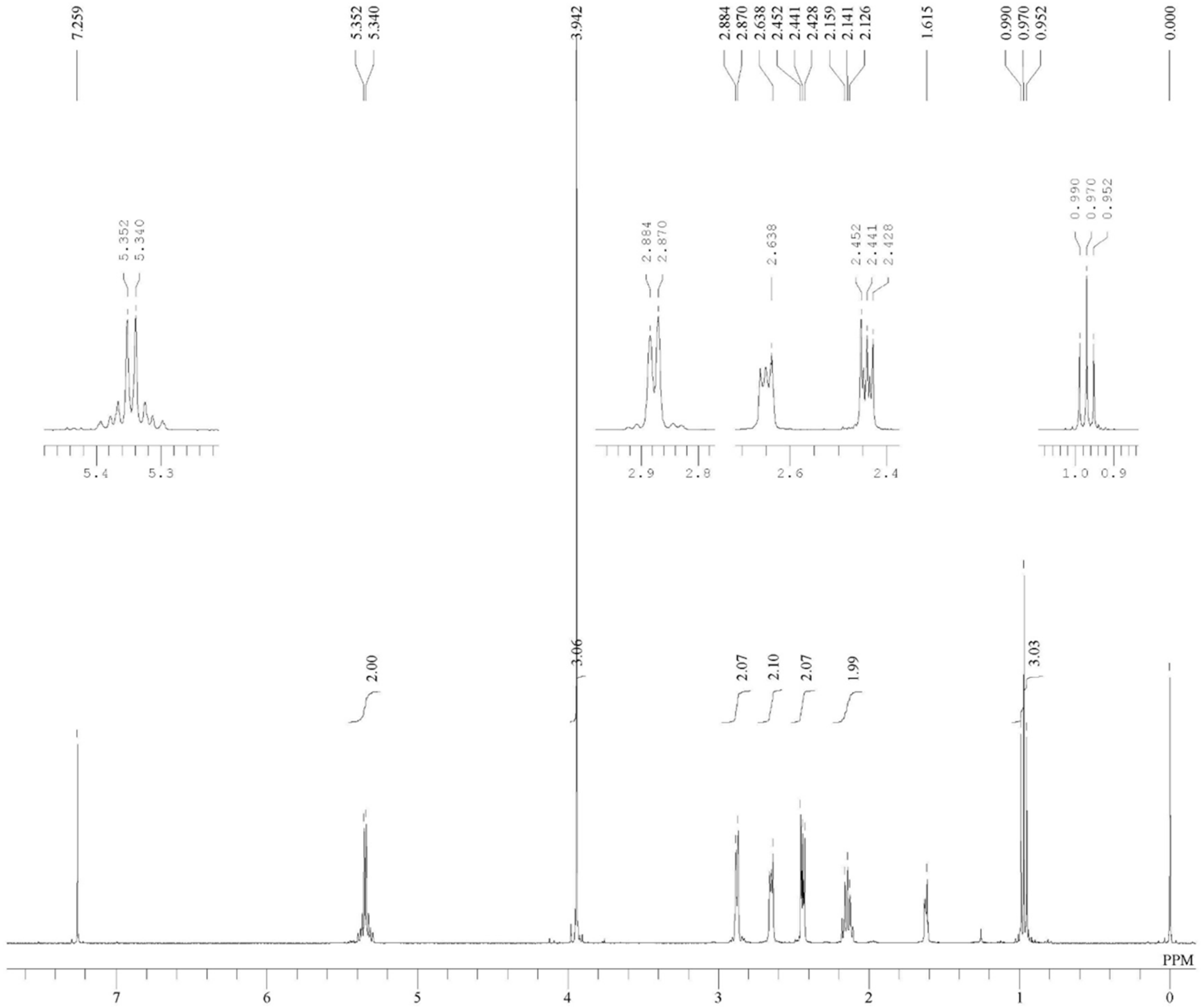

DFILE 28_Proton-1-1.jdf
COMNT single_pulse
DATIM 27-08-2020 12:00:01
OBNUC 1H
EXMOD proton.jxp
OBFRQ 399.78 MHz
OBSET 4.19 KHz
OBFIN 7.29 Hz
POINT 16400
FREQU 7503.00 Hz
SCANS 8
ACQTM 2.1837 sec
PD 5.0000 sec
PW1 2.95 usec
IRNUC 1H
CTEMP 22.3 c
SLVNT CDCL3
EXREF 0.00 ppm
BF 0.25 Hz
RGAIN 58

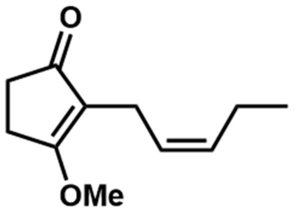

28

DFILE	30 target_Proton-1-1.jdf
COMNT	single_pulse
DATIM	30-07-2020 17:28:15
OBNUC	1H
EXMOD	proton.jxp
OBFRQ	399.78 MHz
OBSET	4.19 KHz
OBFIN	7.29 Hz
POINT	16400
FREQU	7503.00 Hz
SCANS	8
ACQTM	2.1837 sec
PD	5.0000 sec
PW1	2.95 usec
IRNUC	1H
CTEMP	24.3 c
SLVNT	CDCL3
EXREF	0.00 ppm
BF	0.25 Hz
RGAIN	50

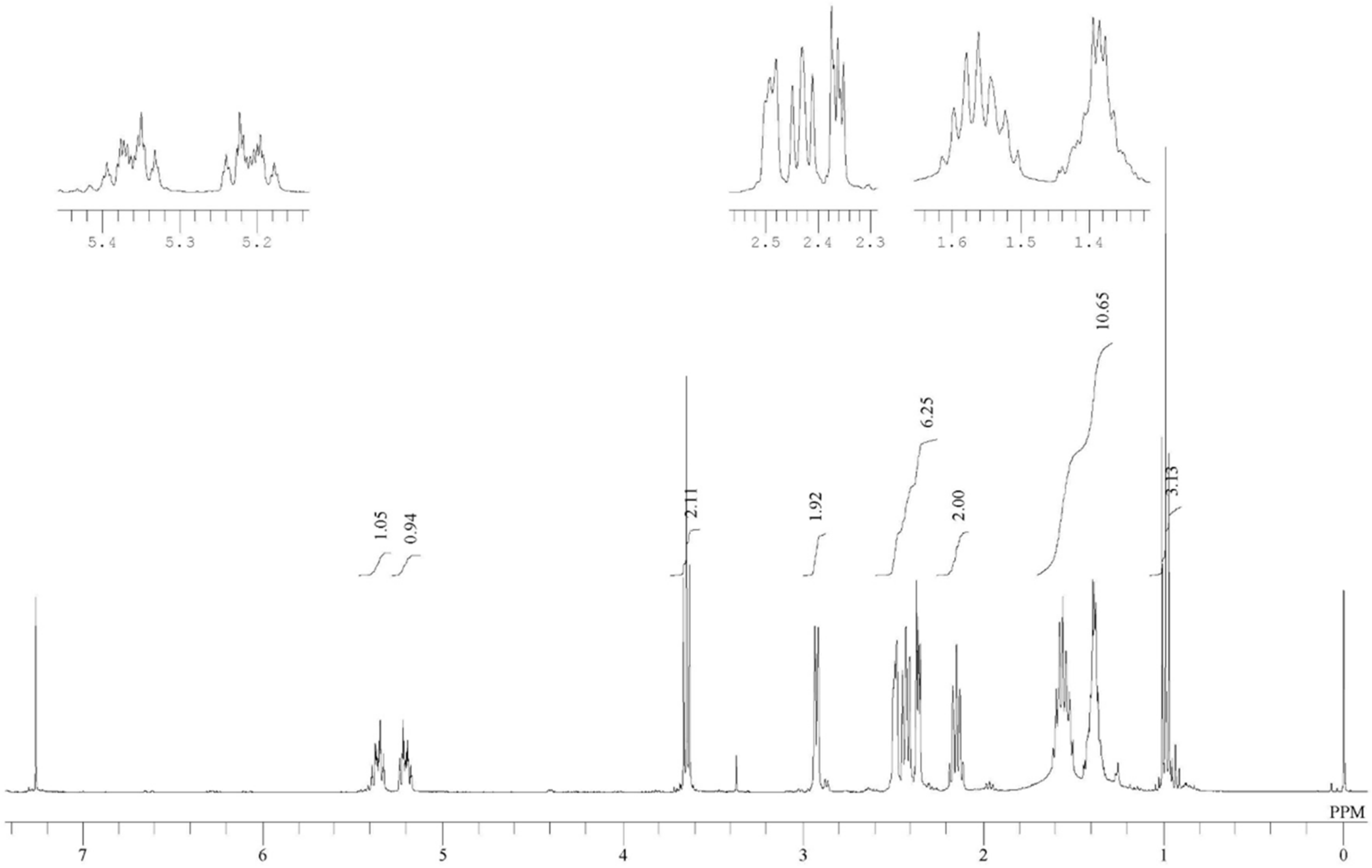

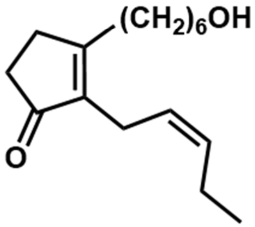

30

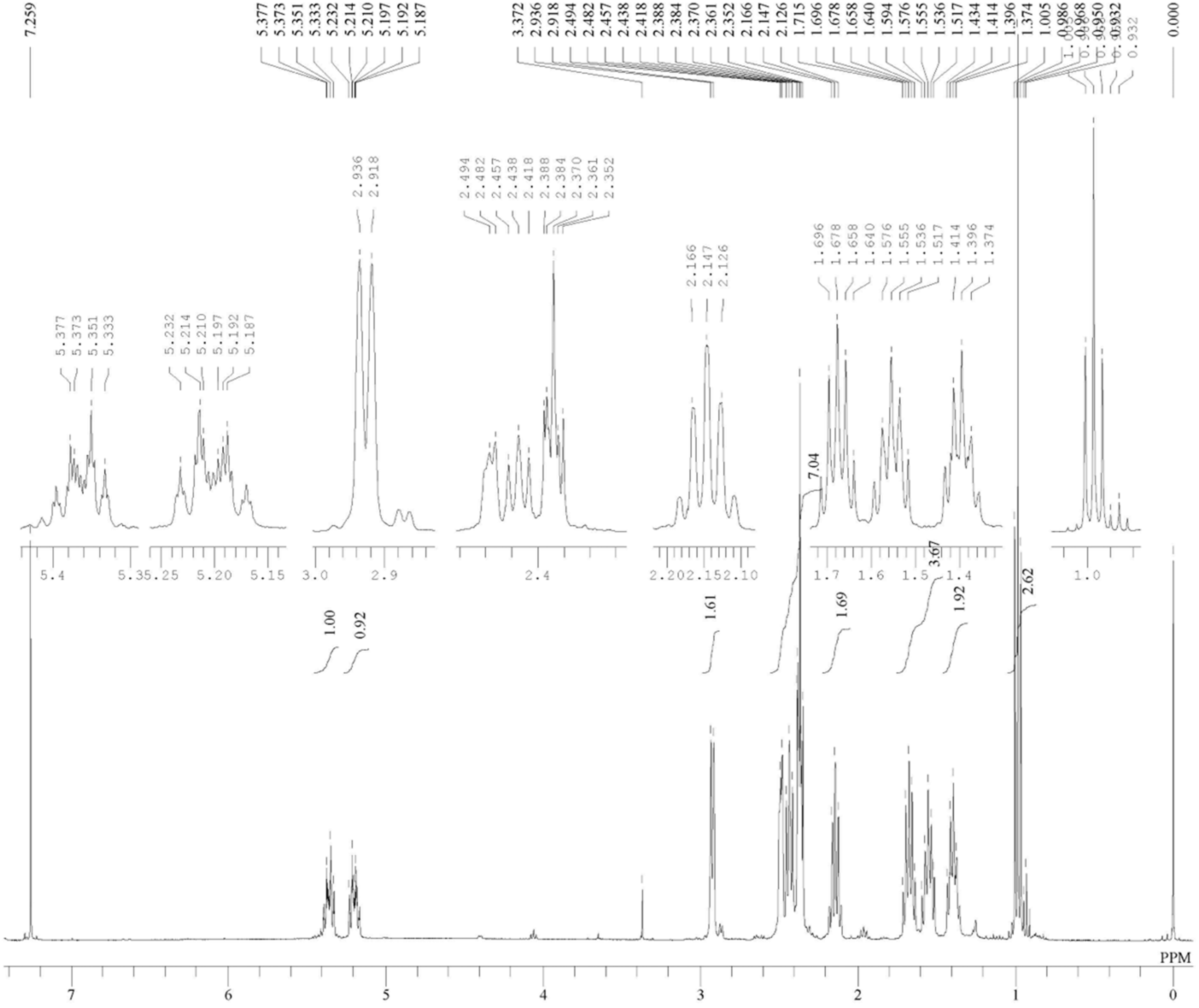
DFILE 5_Proton-1-1.jdf
COMNT single_pulse
DATIM 31-07-2020 18:49:15
OBNUC 1H
EXMOD proton.jxp
OBFRQ 399.78 MHz
OBSET 4.19 KHz
OBFIN 7.29 Hz
POINT 16400
FREQU 7503.00 Hz
SCANS 8
ACQTM 2.1837 sec
PD 5.0000 sec
PW1 2.95 usec
IRNUC 1H
CTEMP 24.3 c
SLVNT CDCL3
EXREF 0.00 ppm
BF 0.25 Hz
RGAIN 54
PPM

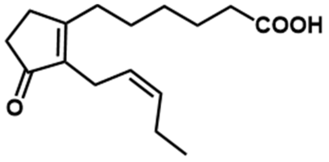
COOH
O

dn-*iso*-OPDA (**5**)

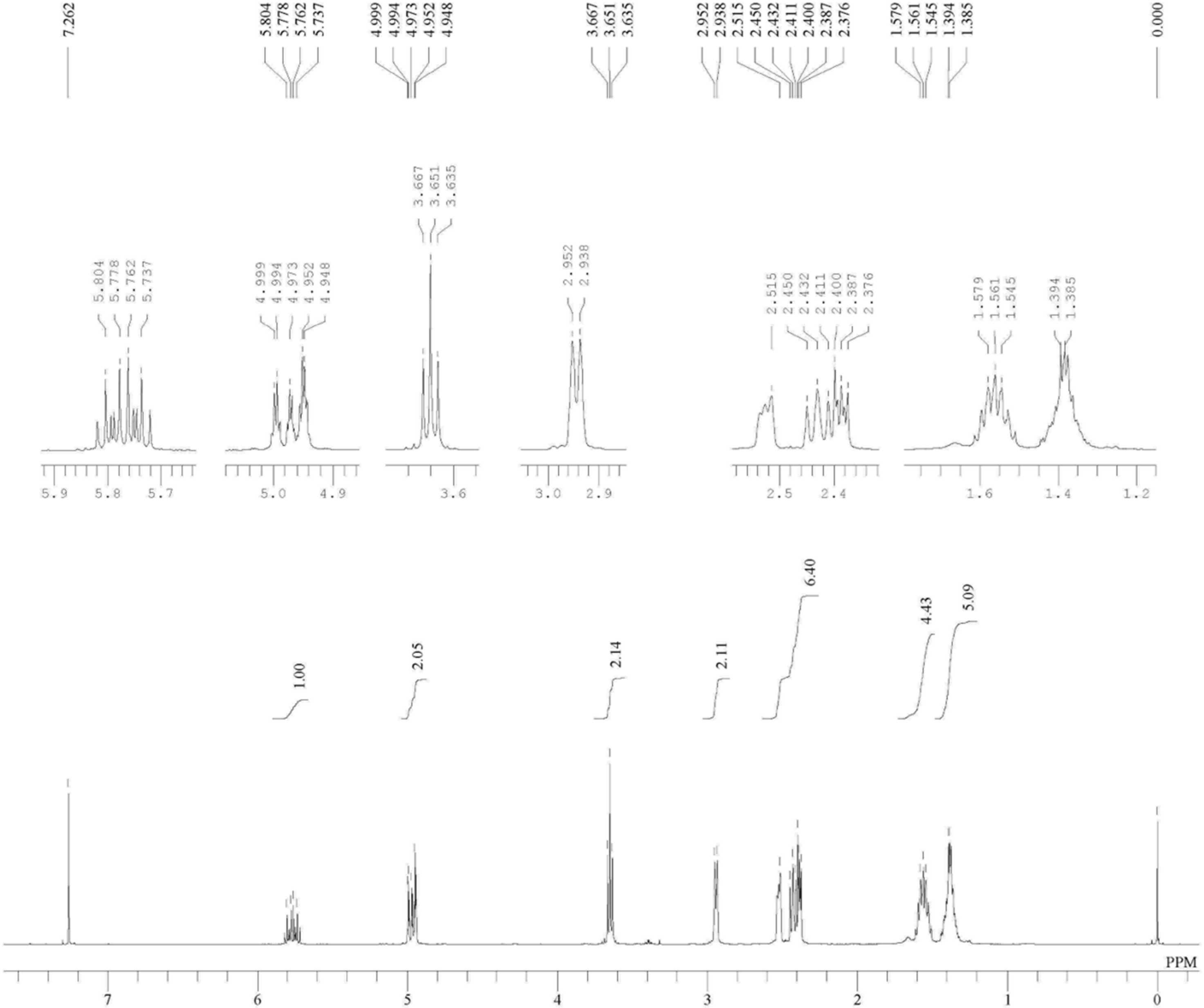
7.262
5.804 5.778 5.762 5.737
4.999 4.994 4.973 4.952 4.948
3.667 3.651 3.635
2.952 2.938 2.515 2.450 2.432 2.411 2.400 2.387 2.376
1.579 1.561 1.545 1.394 1.385
0.000
DFILE 33 fOfŠfjff [f‹_Proton-2-1.jdf
COMNT single_pulse
DATIM 17-11-2020 20:07:47
OBNUC 1H
EXMOD proton.jxp
OBFRQ 399.78 MHz
OBSET 4.19 KHz
OBFIN 7.29 Hz
POINT 16400
FREQU 7503.00 Hz
SCANS 8
ACQTM 2.1837 sec
PD 5.0000 sec
PW1 2.95 usec
IRNUC 1H
CTEMP 21.9 c
SLVNT CDCL3
EXREF 0.00 ppm
BF 0.25 Hz
RGAIN 50
1.00
2.05
2.14
2.11
6.40
4.43
5.09
PPM

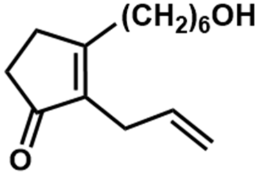
(CH2)6OH
O

33

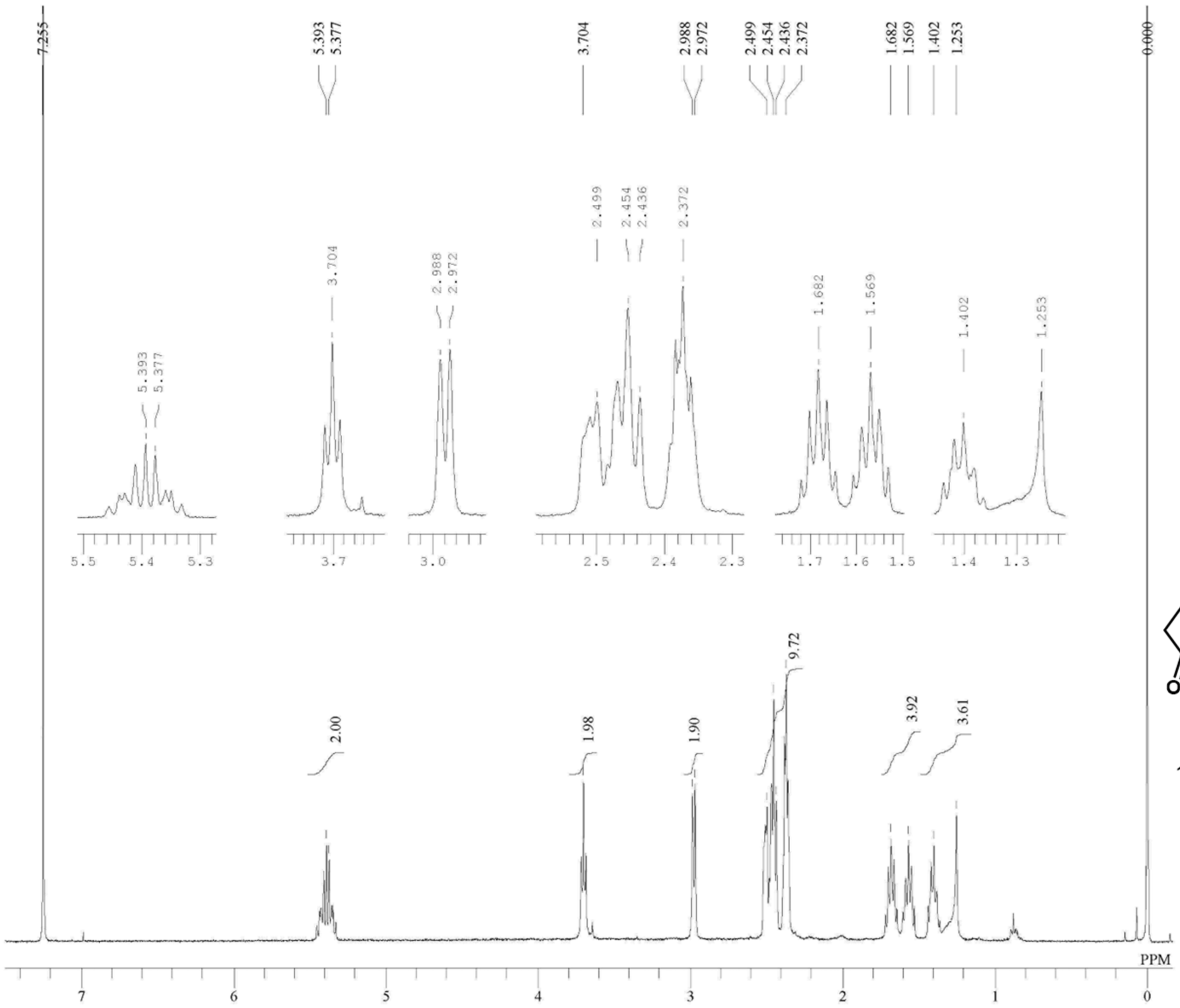

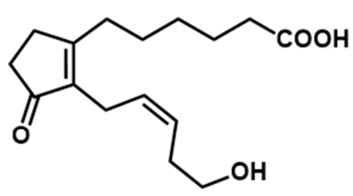

16-OH-dn-*iso*-OPDA (**8**)

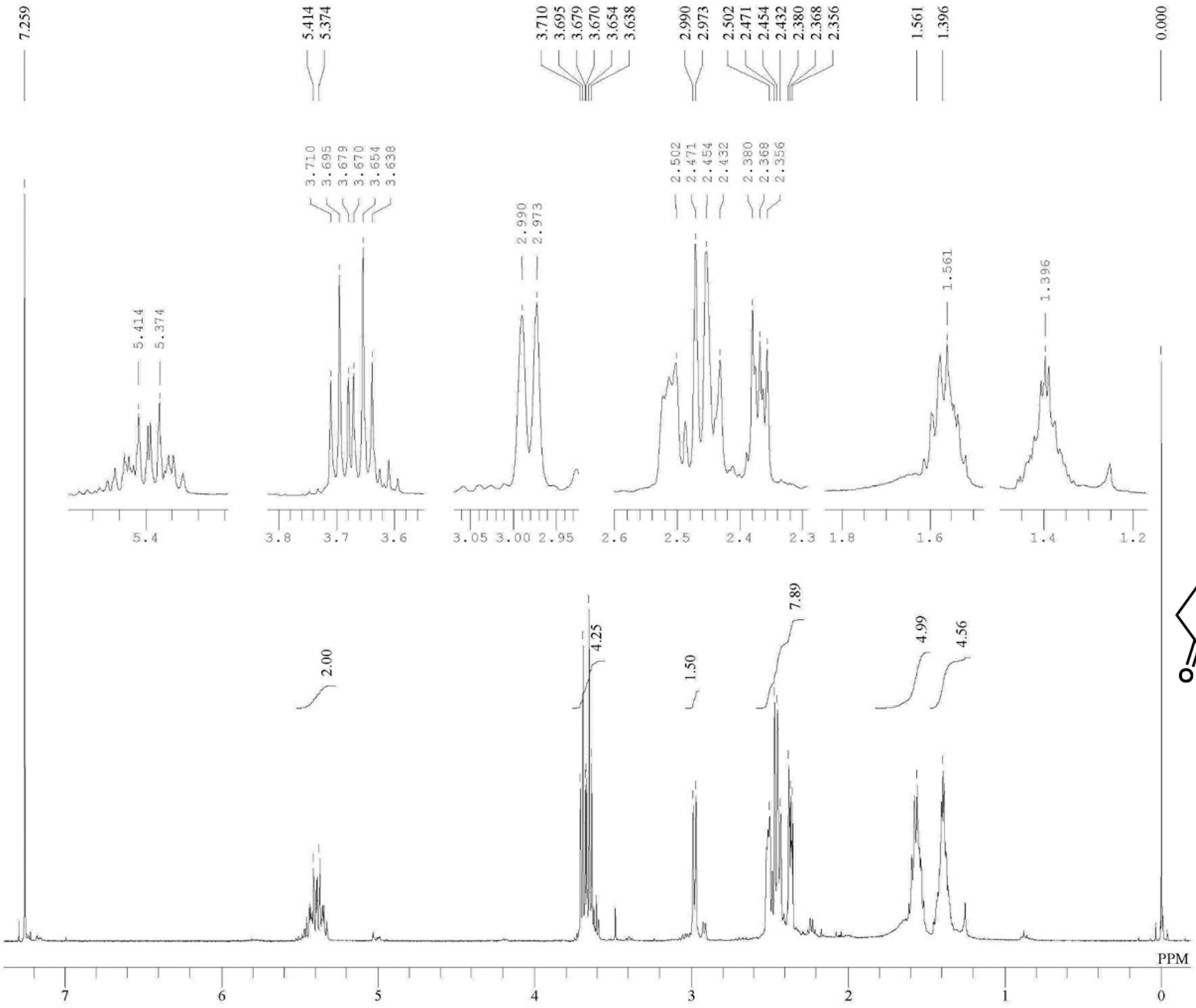

DFILE	35 fWfqfhf fLfV2OH_Proton
COMNT	single_pulse
DATIM	10-11-2020 17:15:42
OBNUC	1H
EXMOD	proton.jxp
OBFRQ	399.78 MHz
OBSET	4.19 KHz
OBFIN	7.29 Hz
POINT	16400
FREQU	7503.00 Hz
SCANS	8
ACQTM	2.1837 sec
PD	5.0000 sec
PW1	2.95 usec
IRNUC	1H
CTEMP	21.8 c
SLVNT	CDCL3
EXREF	0.00 ppm
BF	0.25 Hz
RGAIN	62

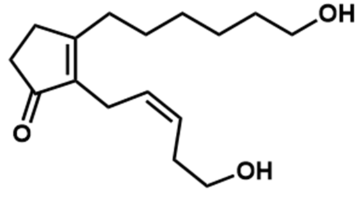

34

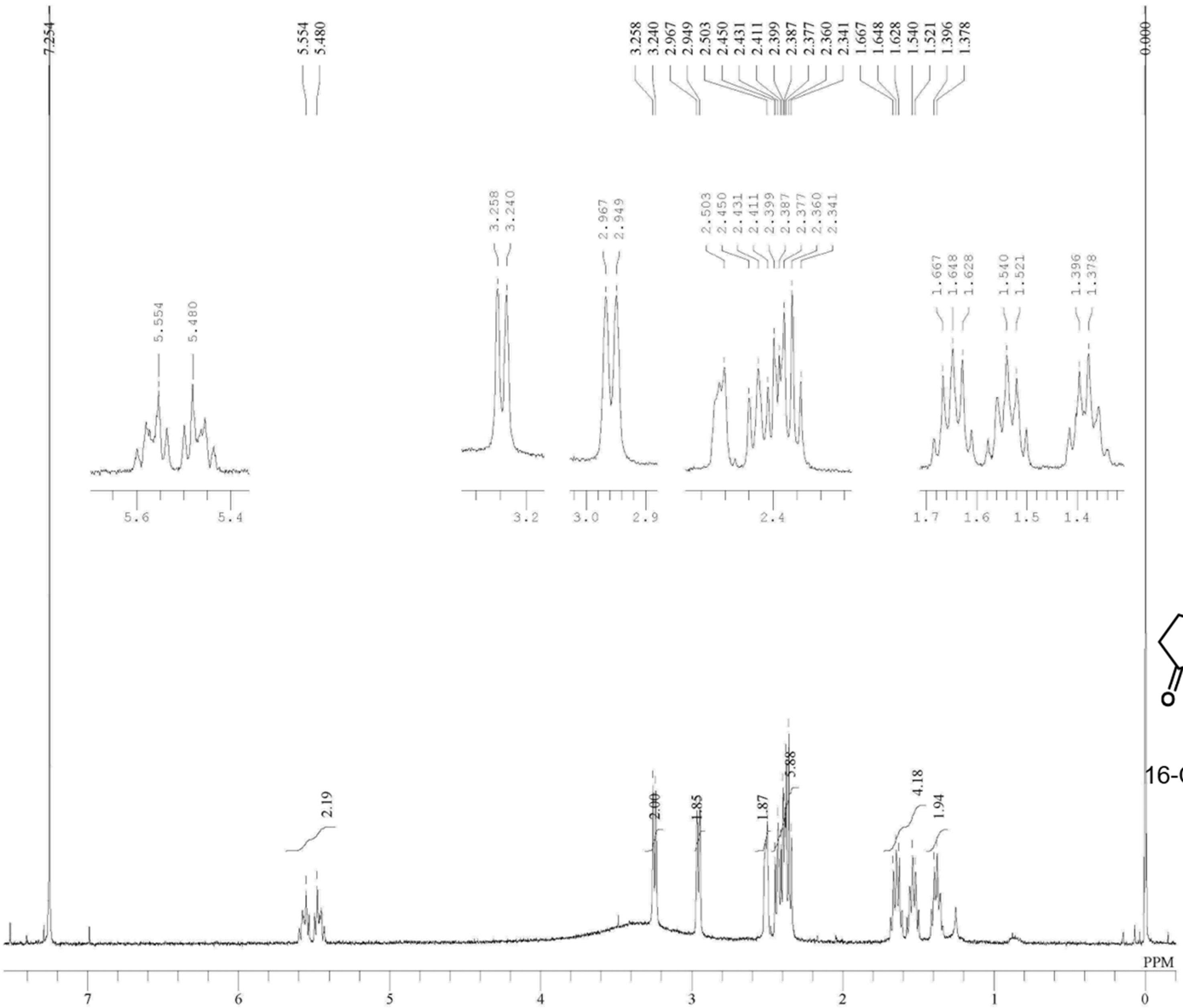

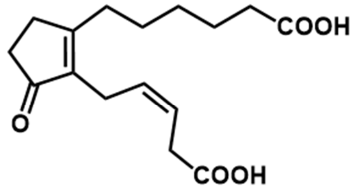

16-COOH-dn-*iso*-OPDA (**9**)

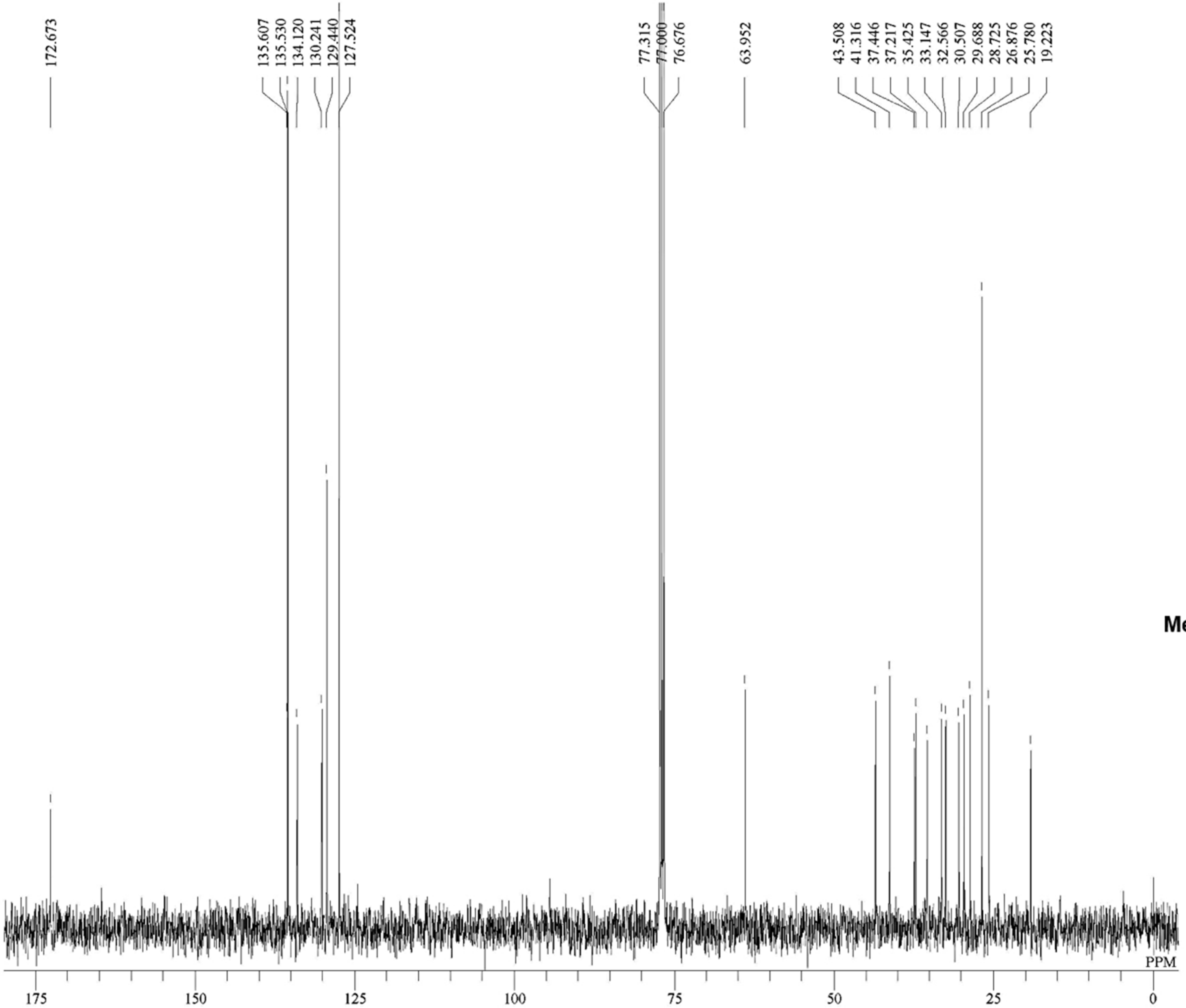

DFILE 13C_17.jdf
COMNT single pulse decoupled gated N
DATIM 02-04-2020 17:35:19
OBNUC 13C
EXMOD carbon.jxp
OBFRQ 100.53 MHz
OBSET 5.35 KHz
OBFIN 5.86 Hz
POINT 32780
FREQU 31407.04 Hz
SCANS 190
ACQTM 0.0000 sec
PD 2.0000 sec
PW1 3.37 usec
IRNUC 1H
CTEMP 22.9 c
SLVNT CDCL3
EXREF 77.00 ppm
BF 0.25 Hz
RGAIN 50

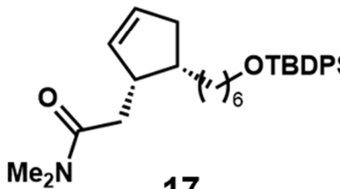

17

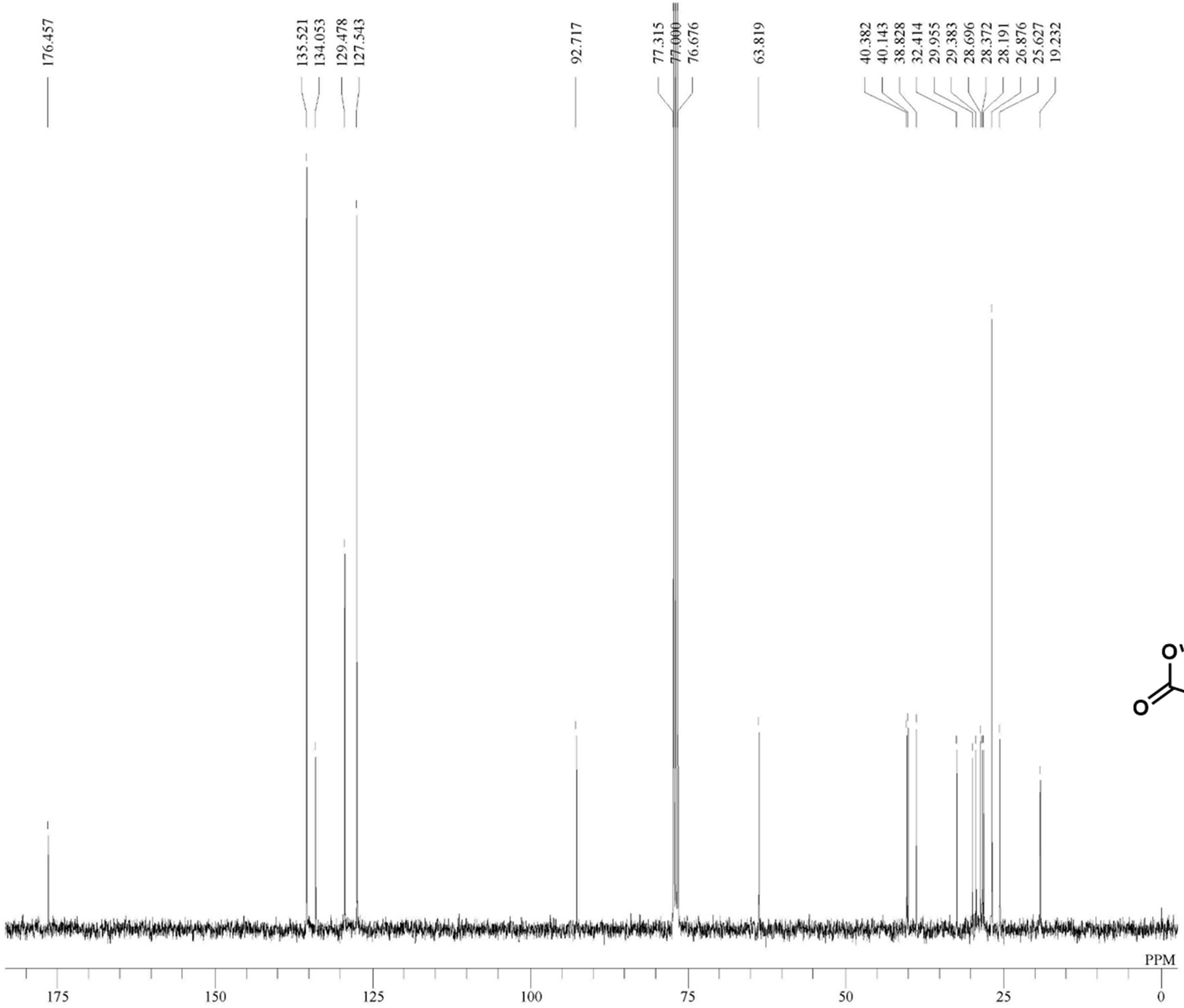
176.457
135.521
134.053
129.478
127.543
92.717
77.315
77.000
76.676
63.819
40.382
40.143
38.828
32.414
29.955
29.383
28.696
28.372
28.191
26.876
25.627
19.232
PPM
175
150
125
100
75
50
25
0
DFILE 13C-iodolactone.jdf
COMNT single pulse decoupled gated N
DATIM 03-04-2020 16:52:56
OBNUC 13C
EXMOD carbon.jxp
OBFRQ 100.53 MHz
OBSET 5.35 KHz
OBFIN 5.86 Hz
POINT 32780
FREQU 31407.04 Hz
SCANS 384
ACQTM 0.0000 sec
PD 2.0000 sec
PW1 3.37 usec
IRNUC 1H
CTEMP 23.5 c
SLVNT CDCL3
EXREF 77.00 ppm
BF 0.25 Hz
RGAIN 50

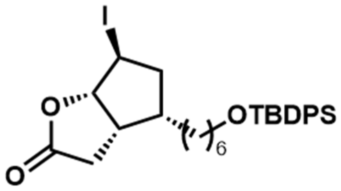
I
O
O
OTBDPS
6

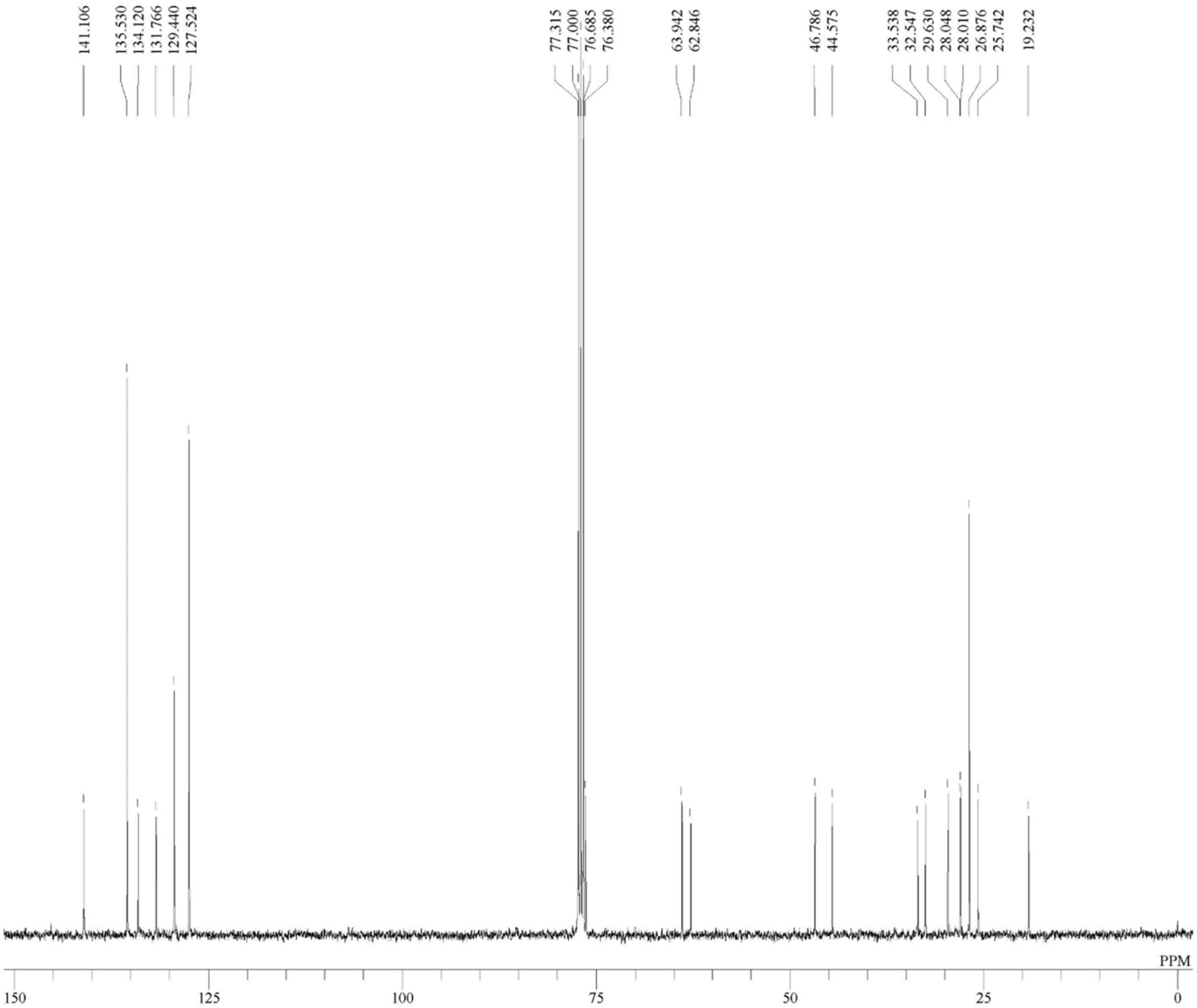

DFILE	13C_18.jdf
COMNT	single pulse decoupled gated N
DATIM	05-04-2020 18:10:58
OBNUC	13C
EXMOD	carbon.jxp
OBFRQ	100.53 MHz
OBSET	5.35 KHz
OBFIN	5.86 Hz
POINT	32780
FREQU	31407.04 Hz
SCANS	784
ACQTM	0.0000 sec
PD	2.0000 sec
PW1	3.37 usec
IRNUC	1H
CTEMP	23.2 c
SLVNT	CDCL3
EXREF	77.00 ppm
BF	0.25 Hz
RGAIN	50

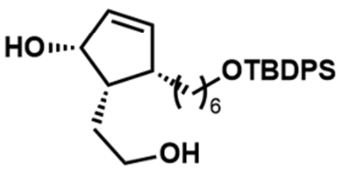

18

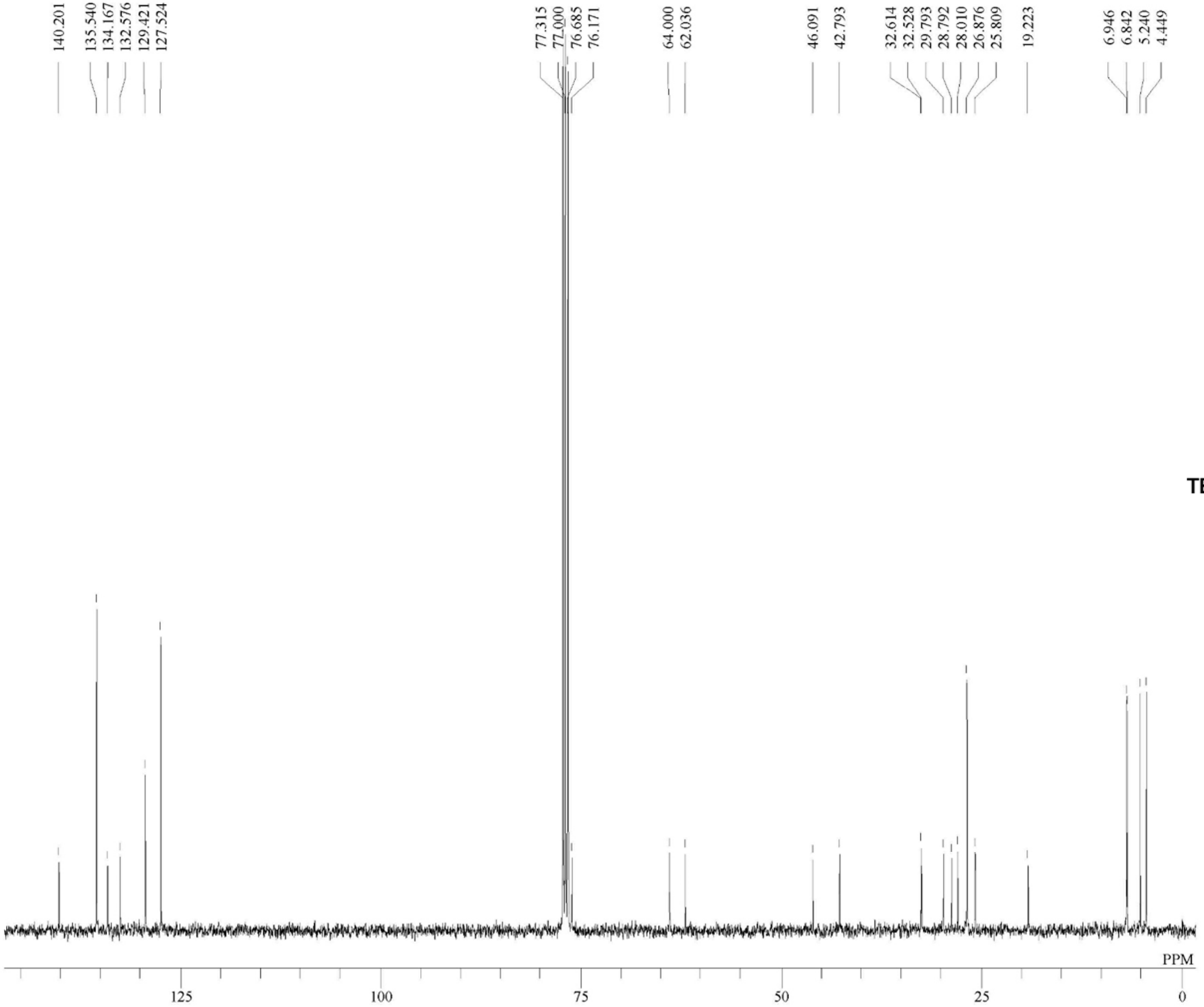

DFILE 13C_19.jdf
COMNT single pulse decoupled gated N
DATIM 07-04-2020 17:36:05
OBNUC 13C
EXMOD carbon.jxp
OBFRQ 100.53 MHz
OBSET 5.35 KHz
OBFIN 5.86 Hz
POINT 32780
FREQU 31407.04 Hz
SCANS 523
ACQTM 0.0000 sec
PD 2.0000 sec
PW1 3.37 usec
IRNUC 1H
CTEMP 23.1 c
SLVNT CDCL3
EXREF 77.00 ppm
BF 0.25 Hz
RGAIN 50

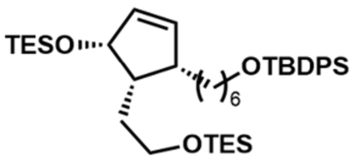

19

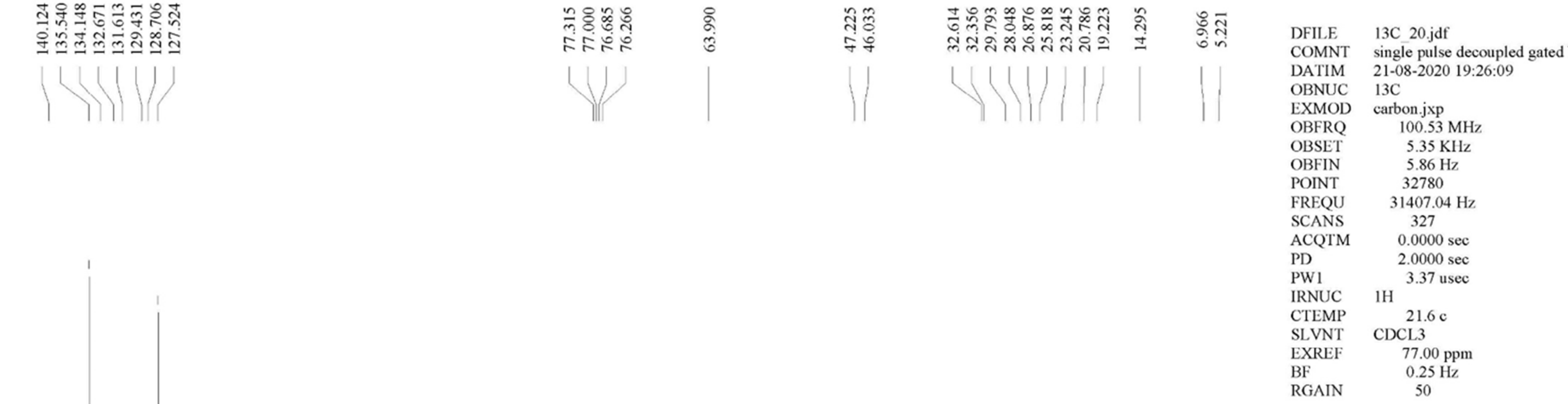
140.124
135.540
134.148
132.671
131.613
129.431
128.706
127.524
77.315
77.000
76.685
76.266
63.990
47.225
46.033
32.614
32.356
29.793
28.048
26.876
25.818
23.245
20.786
19.223
14.295
6.966
5.221
PPM
125
100
75
50
25
0
DFILE 13C_20.jdf
COMNT single pulse decoupled gated N
DATIM 21-08-2020 19:26:09
OBNUC 13C
EXMOD carbon.jxp
OBFRQ 100.53 MHz
OBSET 5.35 KHz
OBFIN 5.86 Hz
POINT 32780
FREQU 31407.04 Hz
SCANS 327
ACQTM 0.0000 sec
PD 2.0000 sec
PW1 3.37 usec
IRNUC 1H
CTEMP 21.6 c
SLVNT CDCL3
EXREF 77.00 ppm
BF 0.25 Hz
RGAIN 50

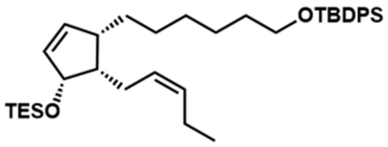
OTBDPS
TESO

20

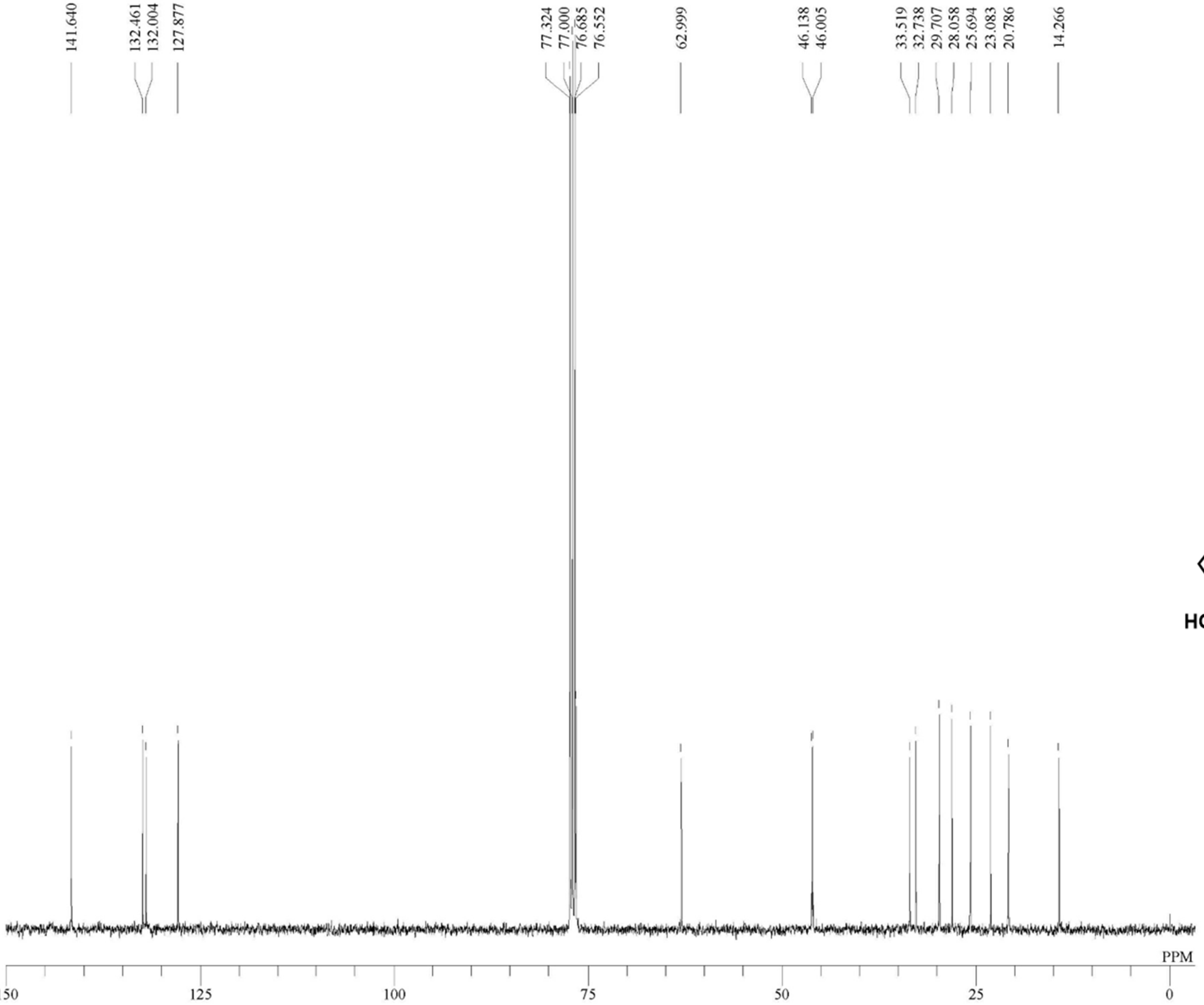

DFILE	13C_diol.jdf
COMNT	single pulse decoupled gated N
DATIM	23-08-2020 20:31:09
OBNUC	13C
EXMOD	carbon.jxp
OBFRQ	100.53 MHz
OBSET	5.35 KHz
OBFIN	5.86 Hz
POINT	32780
FREQU	31407.04 Hz
SCANS	741
ACQTM	0.0000 sec
PD	2.0000 sec
PW1	3.37 usec
IRNUC	1H
CTEMP	21.7 c
SLVNT	CDCL3
EXREF	77.00 ppm
BF	0.25 Hz
RGAIN	50

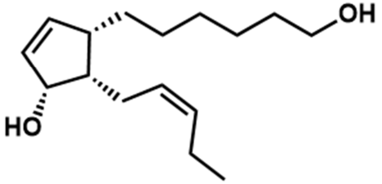

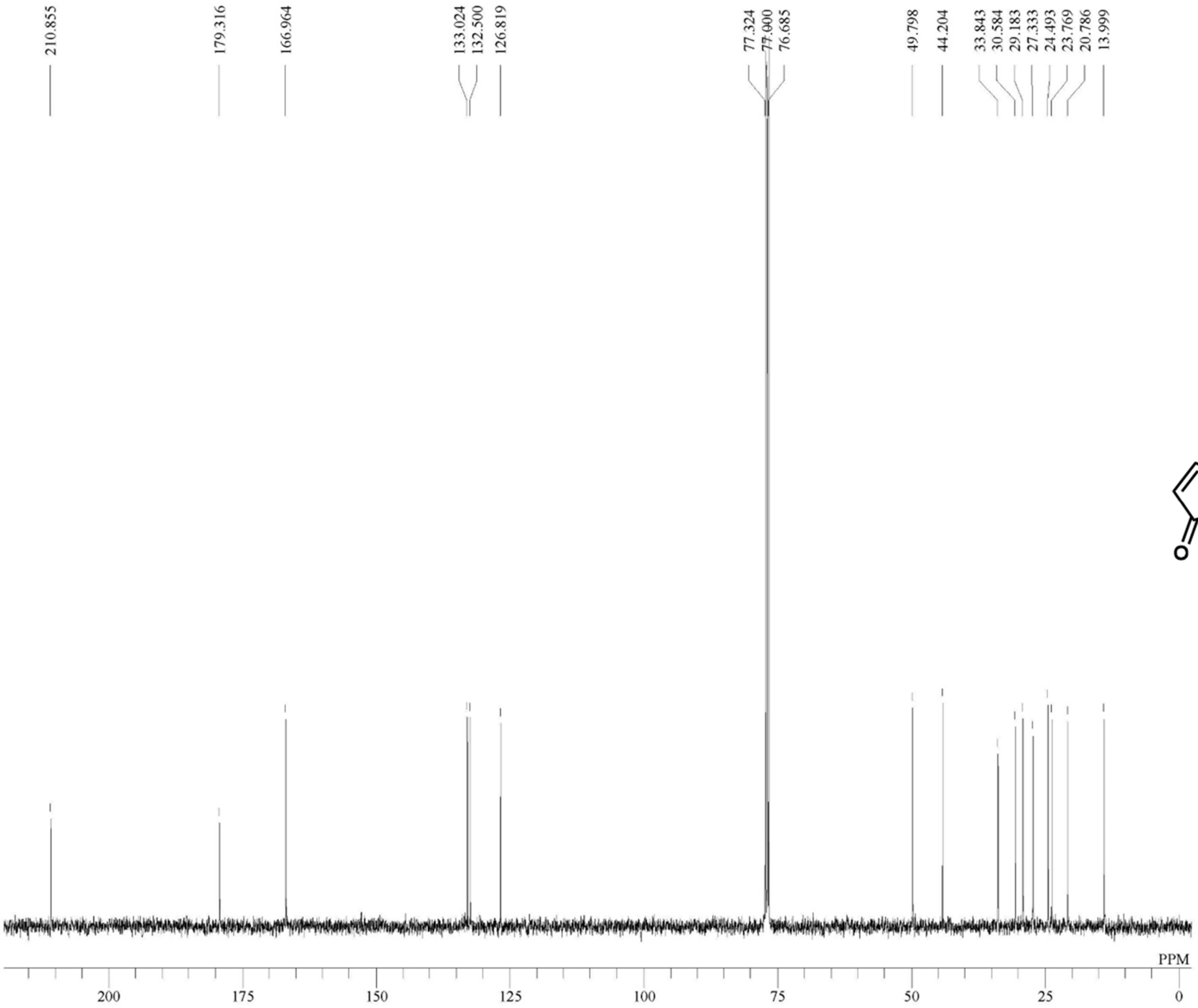

DFILE	13C_04.jdf
COMNT	single pulse decoupled gated N
DATIM	24-08-2020 18:58:19
OBNUC	13C
EXMOD	carbon.jxp
OBFRQ	100.53 MHz
OBSET	5.35 KHz
OBFIN	5.86 Hz
POINT	32780
FREQU	31407.04 Hz
SCANS	337
ACQTM	0.0000 sec
PD	2.0000 sec
PW1	3.37 usec
IRNUC	1H
CTEMP	21.9 c
SLVNT	CDCL3
EXREF	77.00 ppm
BF	0.25 Hz
RGAIN	50

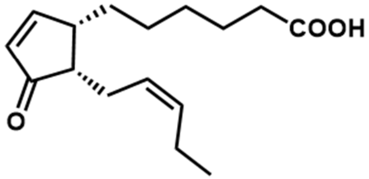

dn-*cis*-OPDA (**4**)

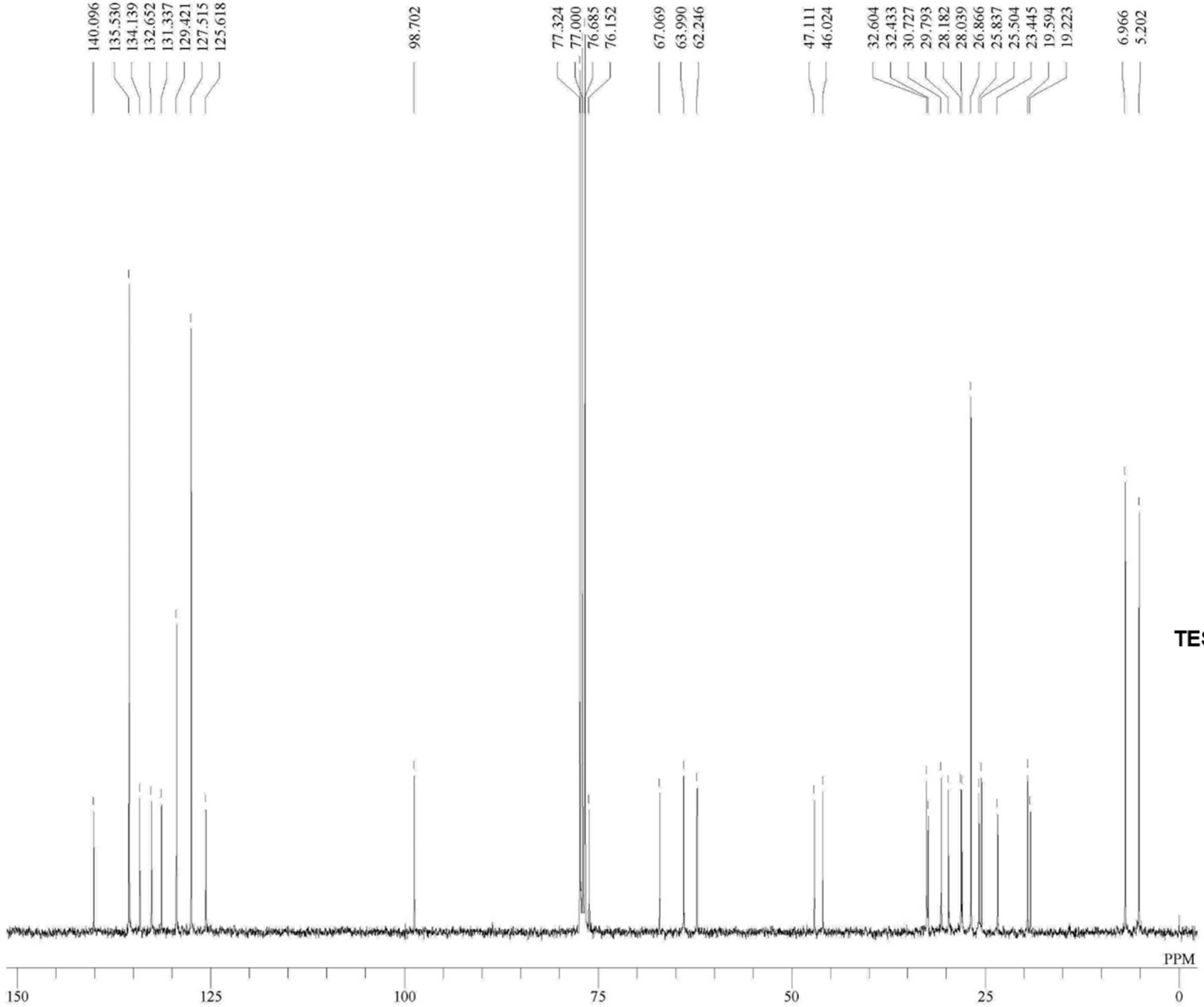

DFILE Wittig_Carbon-1-1.jdf
COMNT single pulse decoupled gated N[illegible]
DATIM 17-08-2020 11:11:59
OBNUC 13C
EXMOD carbon.jxp
OBFRQ 100.53 MHz
OBSET 5.35 KHz
OBFIN 5.86 Hz
POINT 32780
FREQU 31407.04 Hz
SCANS 1024
ACQTM 1.0433 sec
PD 2.0000 sec
PW1 3.37 usec
IRNUC 1H
CTEMP 21.9 c
SLVNT CDCL3
EXREF 77.00 ppm
BF 0.25 Hz
RGAIN 50

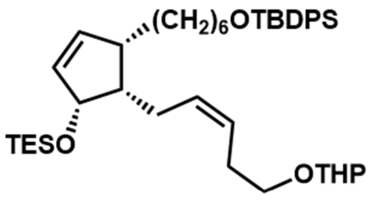

21

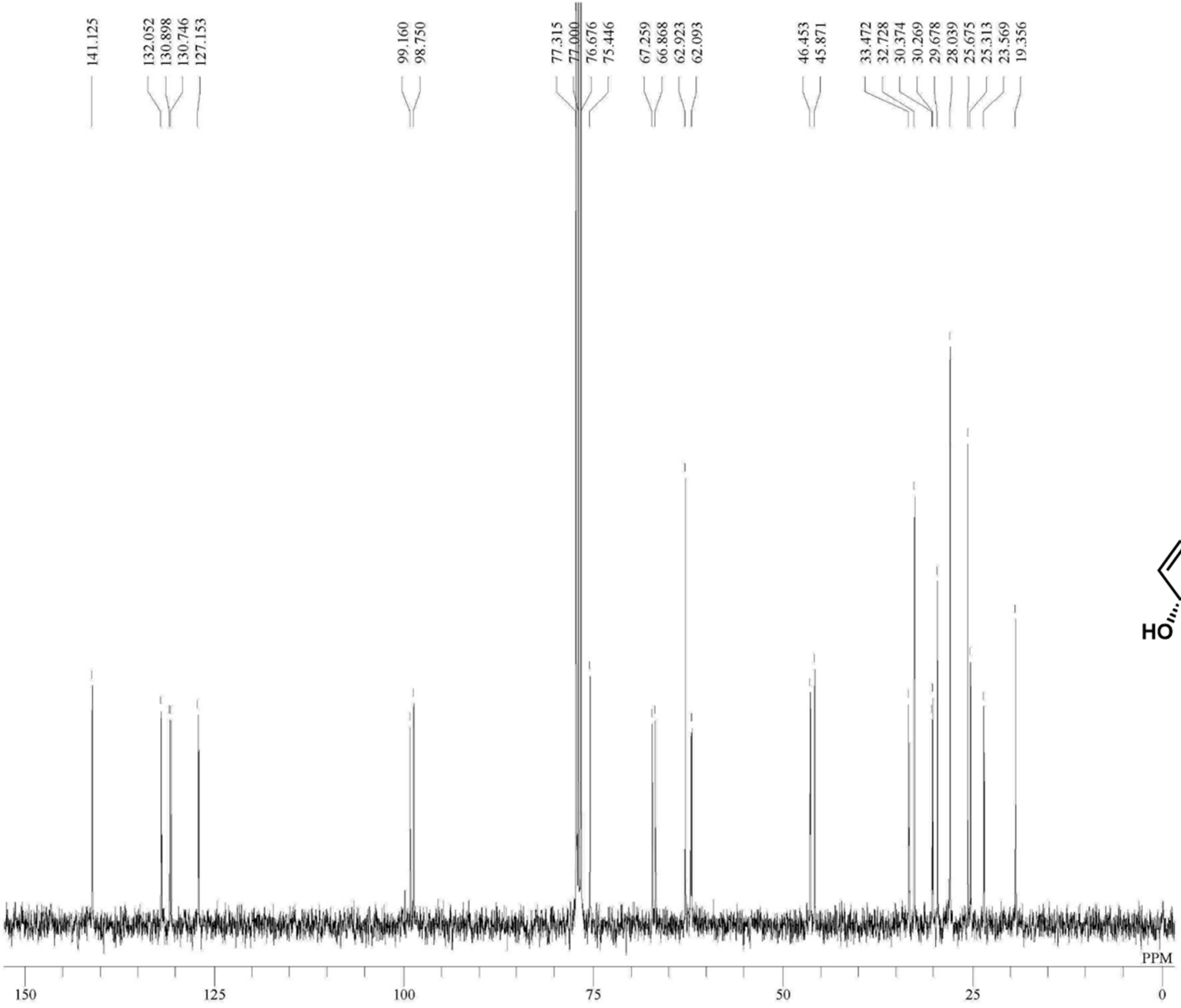
141.125
132.052
130.898
130.746
127.153
99.160
98.750
77.315
77.000
76.676
75.446
67.259
66.868
62.923
62.093
46.453
45.871
33.472
32.728
30.374
30.269
29.678
28.039
25.675
25.313
23.569
19.356
DFILE TBAF_13C-1.jdf
COMNT single pulse decoupled gated N
DATIM 01-09-2020 11:29:24
OBNUC 13C
EXMOD carbon.jxp
OBFRQ 100.53 MHz
OBSET 5.35 KHz
OBFIN 5.86 Hz
POINT 32780
FREQU 31407.04 Hz
SCANS 201
ACQTM 1.0433 sec
PD 2.0000 sec
PW1 3.37 usec
IRNUC 1H
CTEMP 22.6 c
SLVNT CDCL3
EXREF 77.00 ppm
BF 0.25 Hz
RGAIN 50
PPM
150
125
100
75
50
25
0

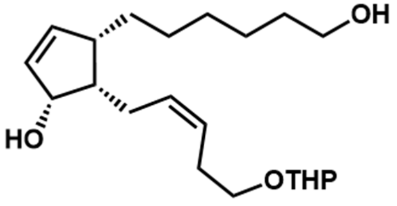
OH
HO
OTHP

22

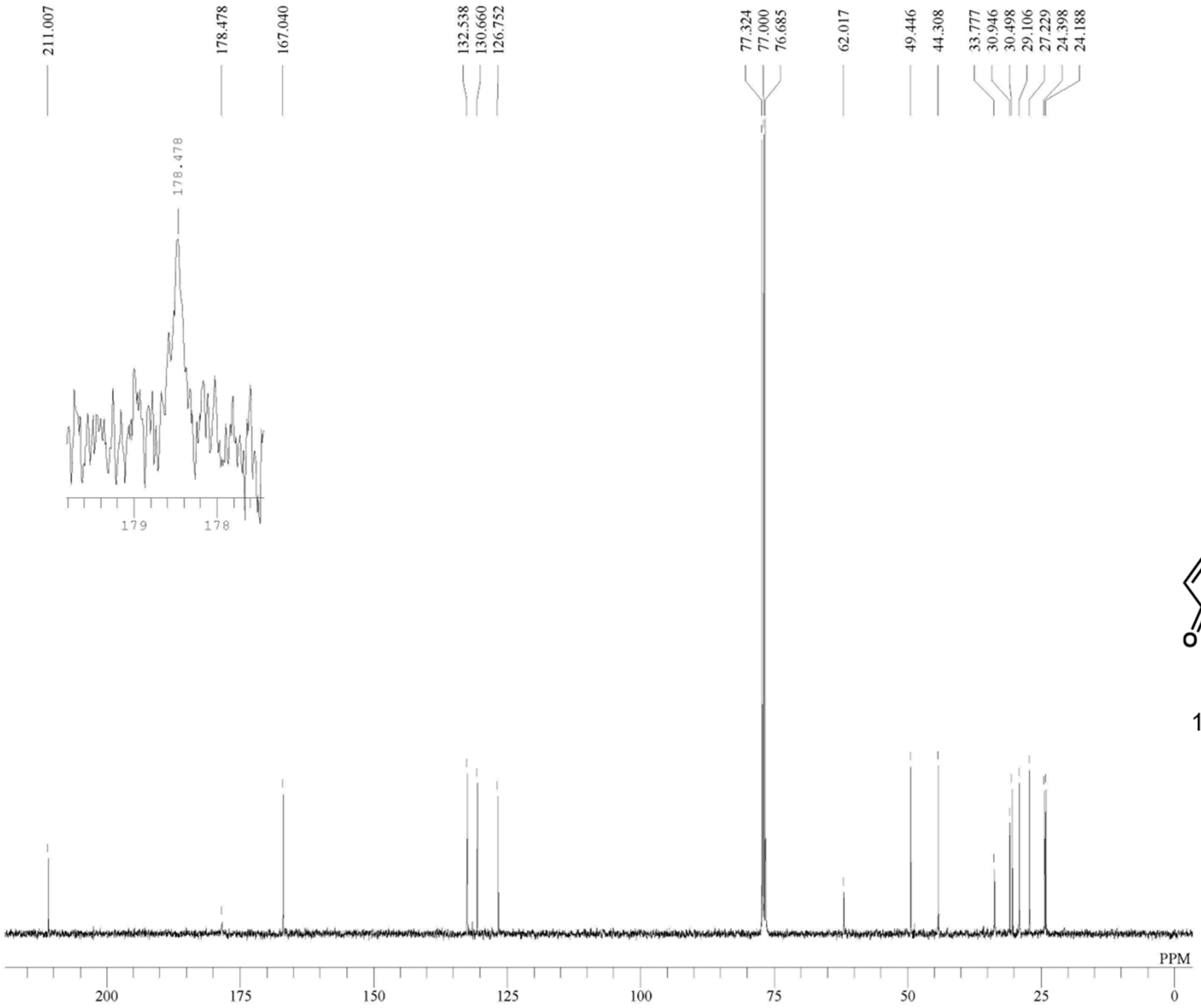

DFILE	16OHdncisOPDA_Carbon-1.jd
COMNT	single pulse decoupled gated N
DATIM	02-12-2020 17:35:13
OBNUC	13C
EXMOD	carbon.jxp
OBFRQ	100.53 MHz
OBSET	5.35 KHz
OBFIN	5.86 Hz
POINT	32780
FREQU	31407.04 Hz
SCANS	1024
ACQTM	1.0433 sec
PD	2.0000 sec
PW1	3.37 usec
IRNUC	1H
CTEMP	23.2 c
SLVNT	CDCL3
EXREF	77.00 ppm
BF	0.25 Hz
RGAIN	50

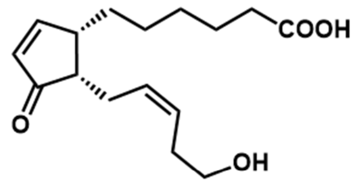

16OH-dn-*cis*-OPDA (**6**)

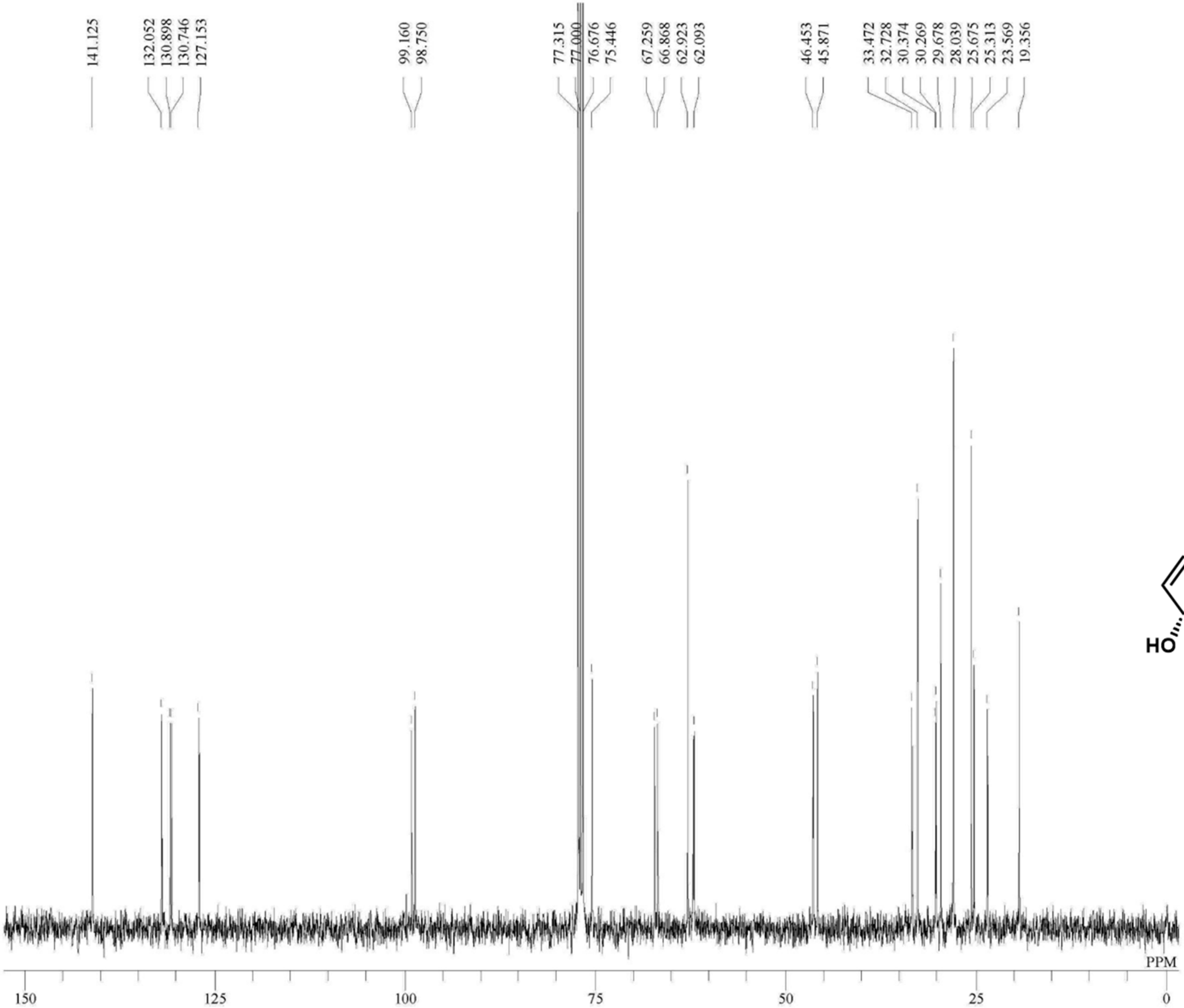

DFILE TBAF_13C-1.jdf
COMNT single pulse decoupled gated N
DATIM 01-09-2020 11:29:24
OBNUC 13C
EXMOD carbon.jxp
OBFRQ 100.53 MHz
OBSET 5.35 KHz
OBFIN 5.86 Hz
POINT 32780
FREQU 31407.04 Hz
SCANS 201
ACQTM 1.0433 sec
PD 2.0000 sec
PW1 3.37 usec
IRNUC 1H
CTEMP 22.6 c
SLVNT CDCL3
EXREF 77.00 ppm
BF 0.25 Hz
RGAIN 50

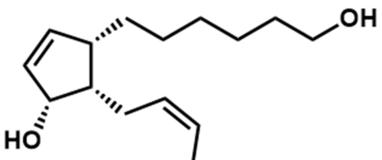

23

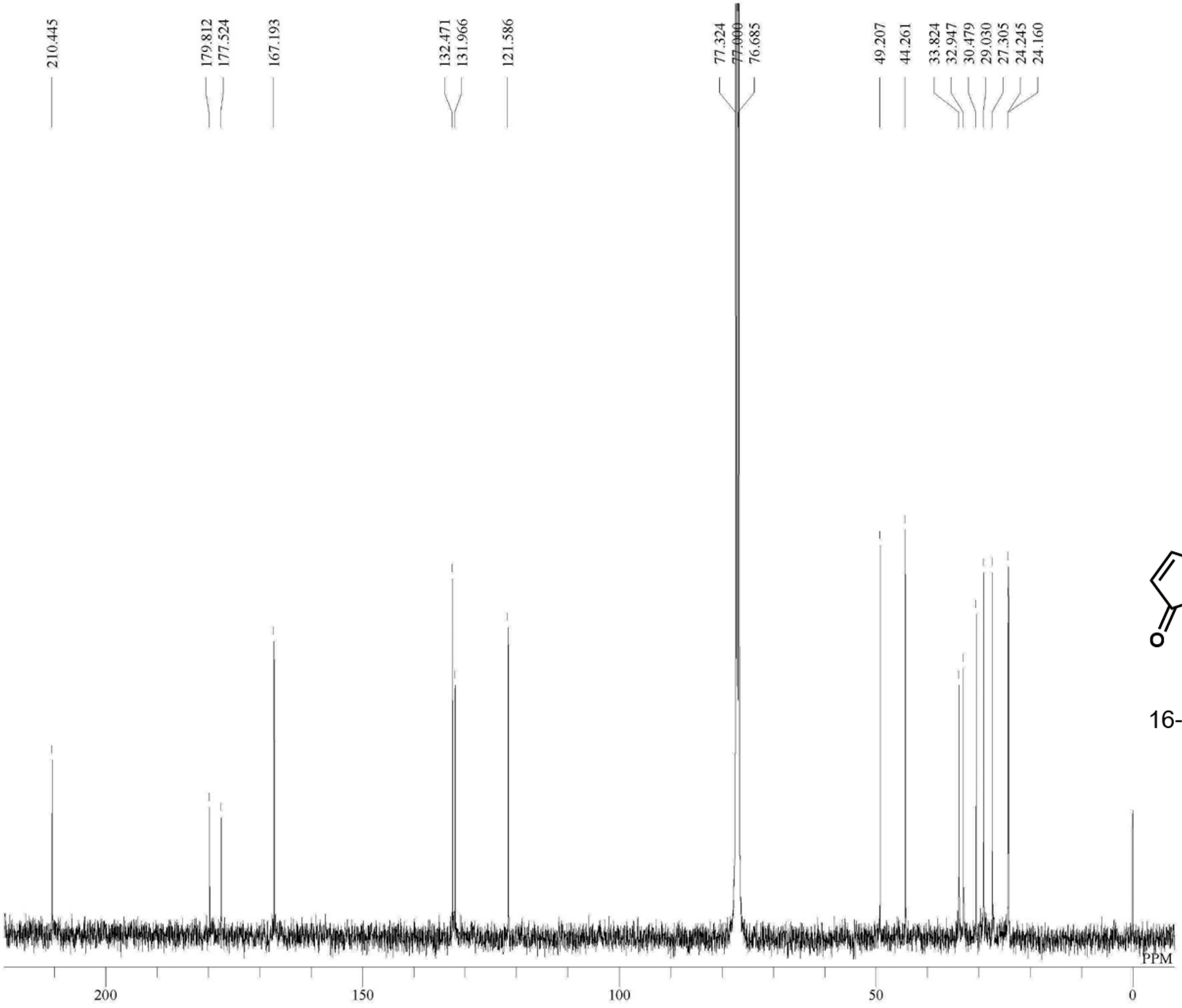

DFILE 07179_13C-1.jdf
COMNT single pulse decoupled gated N
DATIM 18-11-2020 22:59:16
OBNUC 13C
EXMOD carbon.jxp
OBFRQ 100.53 MHz
OBSET 5.35 KHz
OBFIN 5.86 Hz
POINT 32780
FREQU 31407.04 Hz
SCANS 10000
ACQTM 1.0433 sec
PD 2.0000 sec
PW1 3.37 usec
IRNUC 1H
CTEMP 21.9 c
SLVNT CDCL3
EXREF 77.00 ppm
BF 0.25 Hz
RGAIN 50

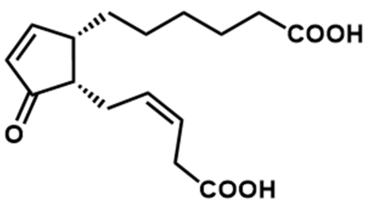

16-COOH-dn-*cis*-OPDA (**7**)

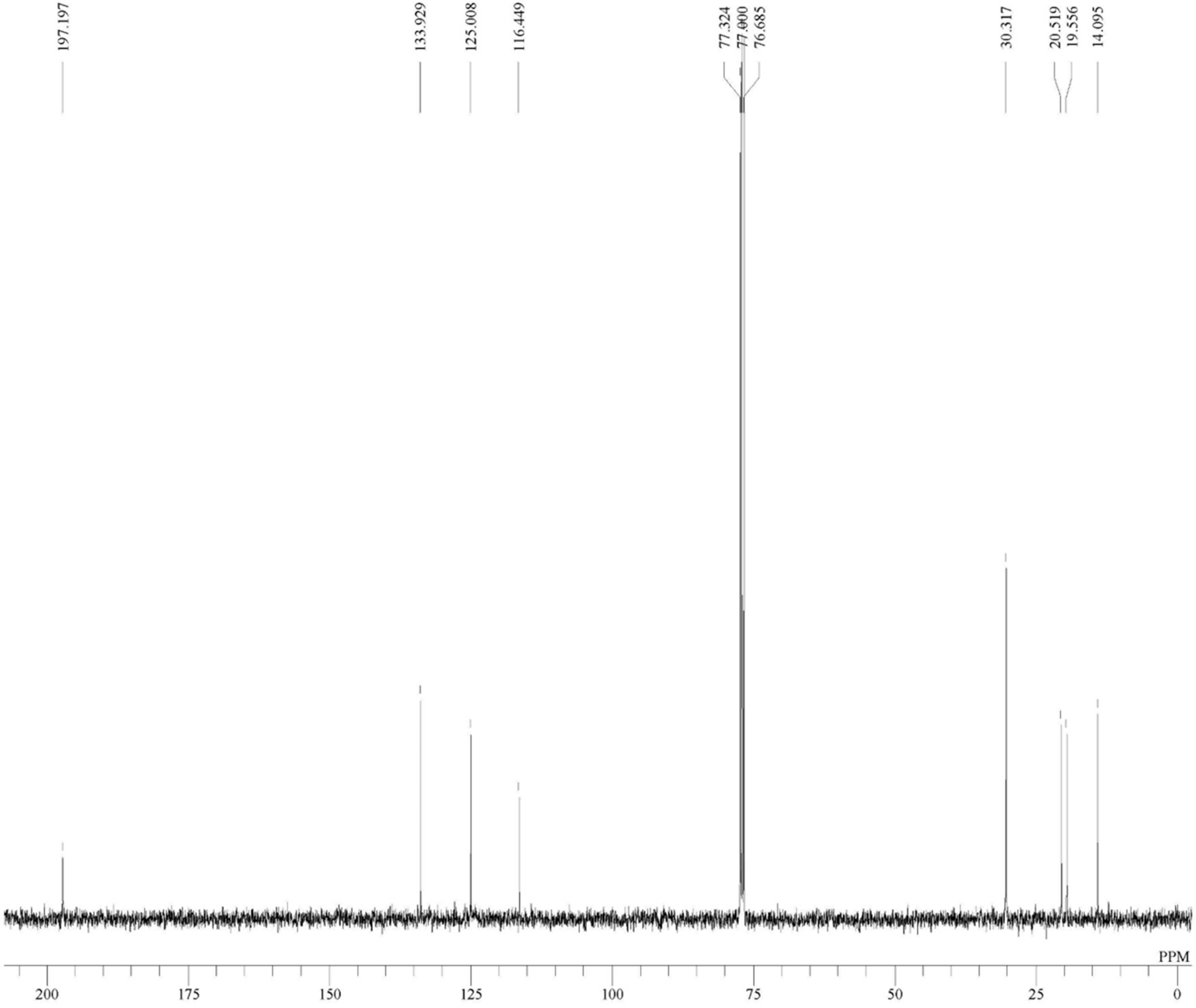

DFILE 27_Carbon-1-1.jdf
COMNT single pulse decoupled gated N
DATIM 19-08-2020 19:38:27
OBNUC 13C
EXMOD carbon.jxp
OBFRQ 100.53 MHz
OBSET 5.35 KHz
OBFIN 5.86 Hz
POINT 32780
FREQU 31407.04 Hz
SCANS 208
ACQTM 1.0433 sec
PD 2.0000 sec
PW1 3.37 usec
IRNUC 1H
CTEMP 21.6 c
SLVNT CDCL3
EXREF 77.00 ppm
BF 0.25 Hz
RGAIN 50

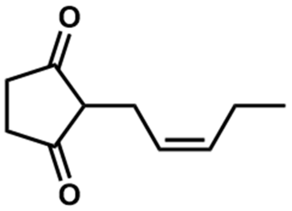

27

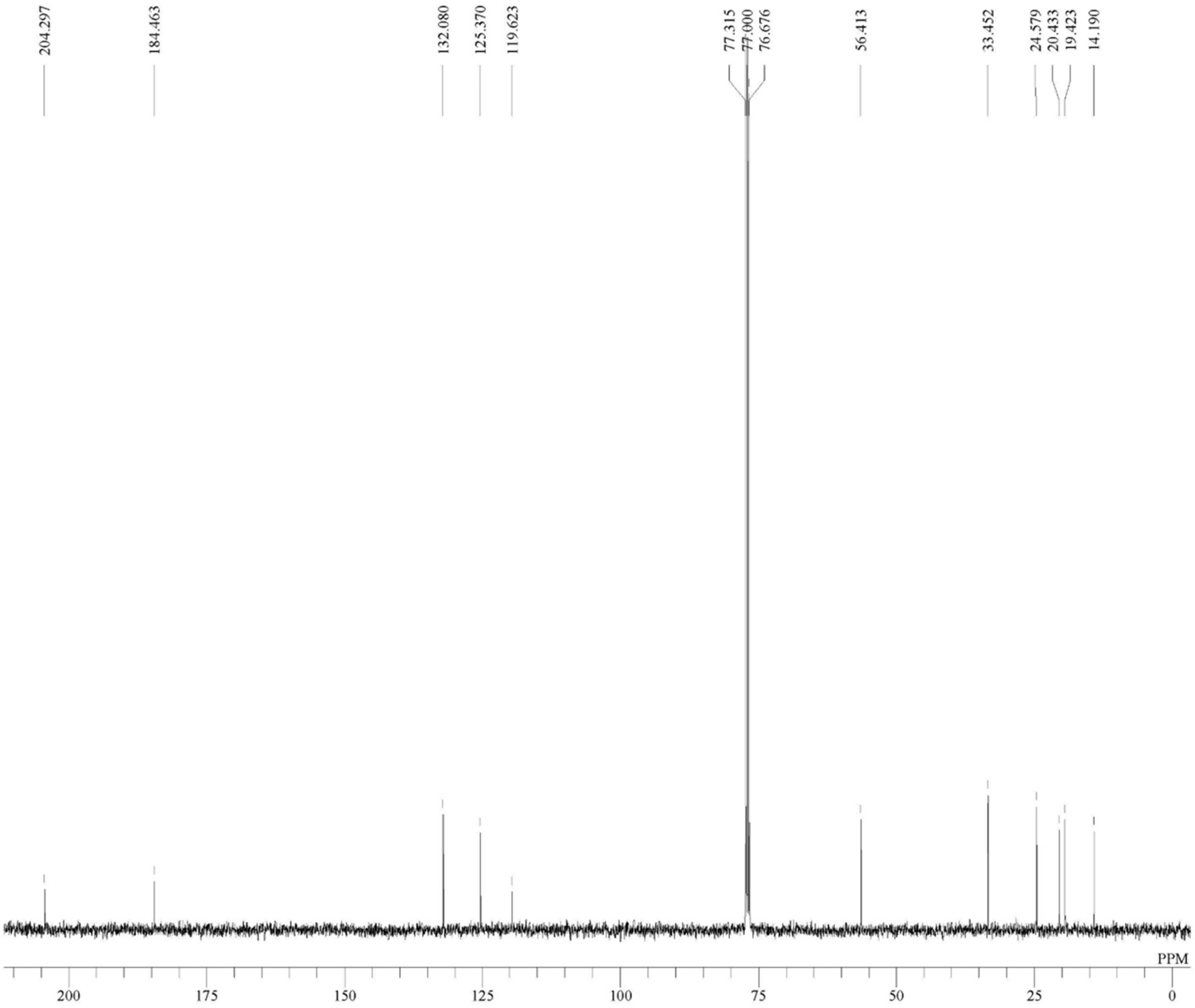

DFILE	28 _Carbon-2-1.jdf
COMNT	single pulse decoupled gated N
DATIM	25-08-2020 12:04:35
OBNUC	13C
EXMOD	carbon.jxp
OBFRQ	100.53 MHz
OBSET	5.35 KHz
OBFIN	5.86 Hz
POINT	32780
FREQU	31407.04 Hz
SCANS	520
ACQTM	1.0433 sec
PD	2.0000 sec
PW1	3.37 usec
IRNUC	1H
CTEMP	21.7 c
SLVNT	CDCL3
EXREF	77.00 ppm
BF	0.25 Hz
RGAIN	50

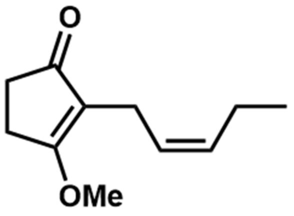

28

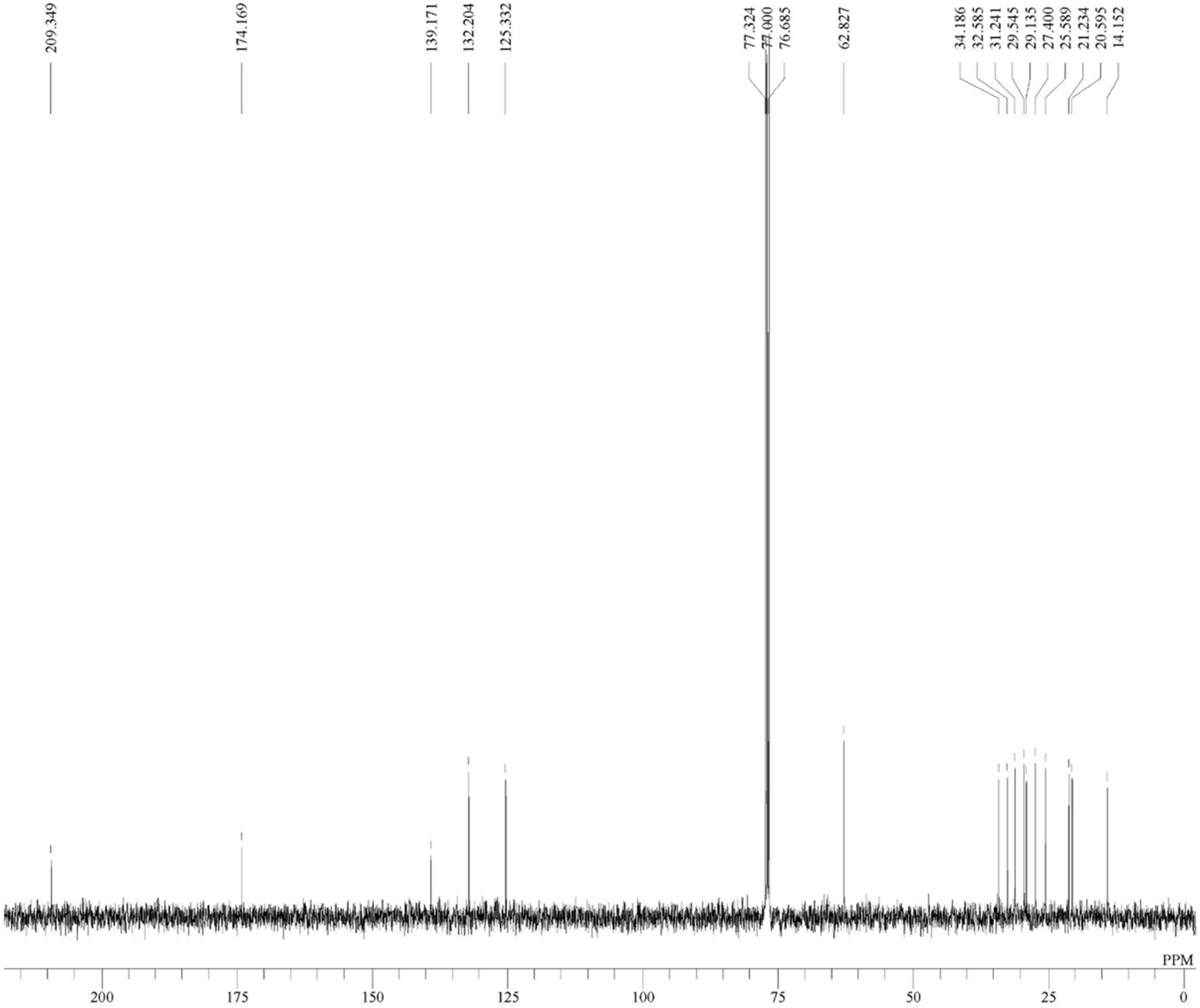

DFILE	30 target_Carbon-1-1.jdf
COMNT	single pulse decoupled gated N
DATIM	30-07-2020 17:29:56
OBNUC	13C
EXMOD	carbon.jxp
OBFRQ	100.53 MHz
OBSET	5.35 KHz
OBFIN	5.86 Hz
POINT	32780
FREQU	31407.04 Hz
SCANS	99
ACQTM	1.0433 sec
PD	2.0000 sec
PW1	3.37 usec
IRNUC	1H
CTEMP	24.3 c
SLVNT	CDCL3
EXREF	77.00 ppm
BF	0.25 Hz
RGAIN	50

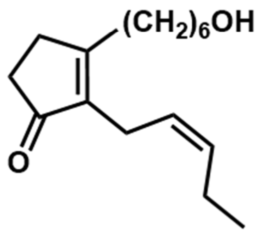

30

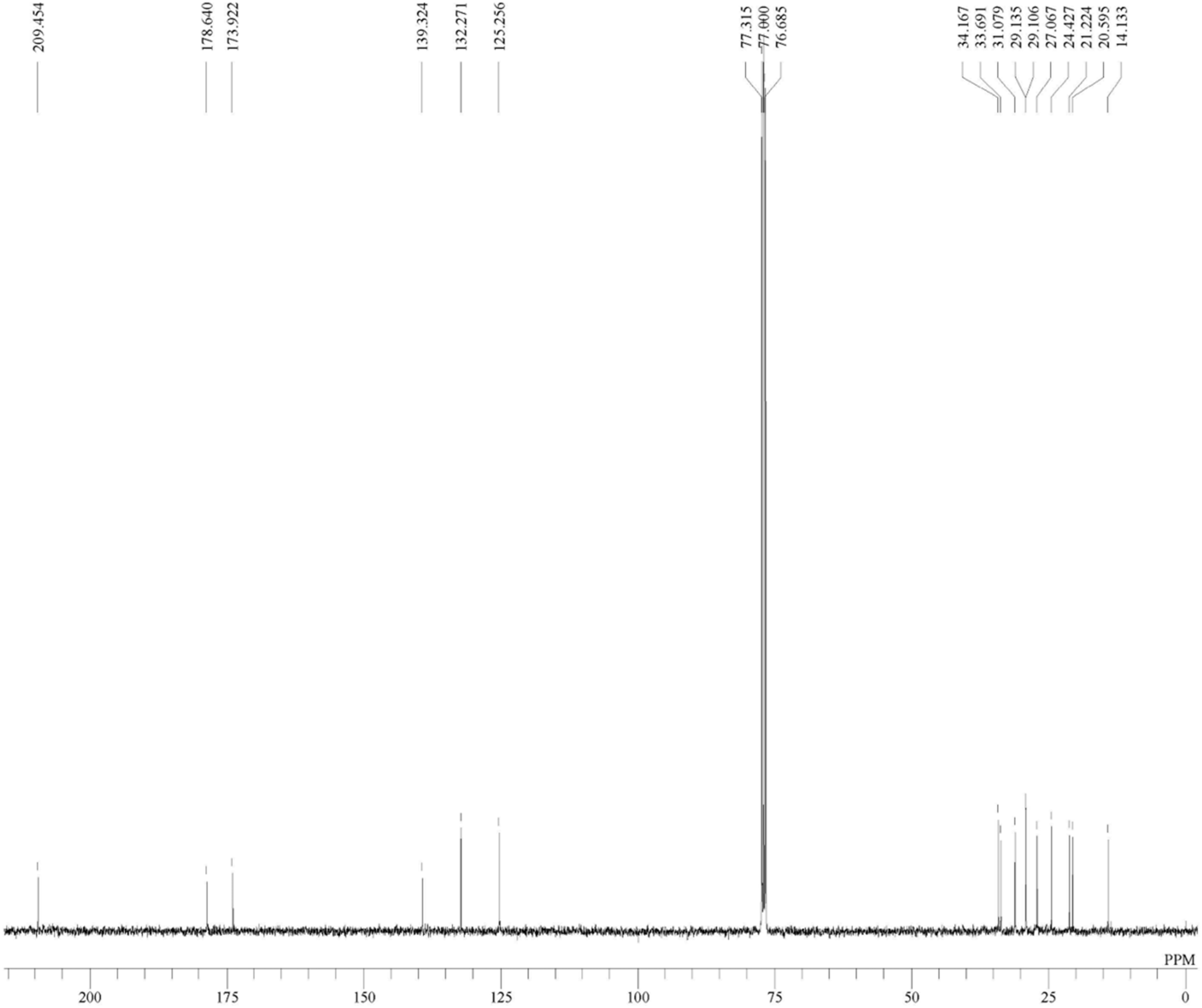

DFILE	5_Carbon-1-1.jdf
COMNT	single pulse decoupled gated N
DATIM	31-07-2020 18:50:42
OBNUC	13C
EXMOD	carbon.jxp
OBFRQ	100.53 MHz
OBSET	5.35 KHz
OBFIN	5.86 Hz
POINT	32780
FREQU	31407.04 Hz
SCANS	1032
ACQTM	1.0433 sec
PD	2.0000 sec
PW1	3.37 usec
IRNUC	1H
CTEMP	24.3 c
SLVNT	CDCL3
EXREF	77.00 ppm
BF	0.25 Hz
RGAIN	50

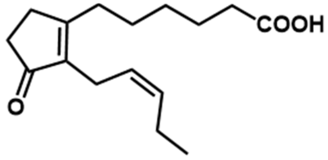

dn-*iso*-OPDA (**5**)

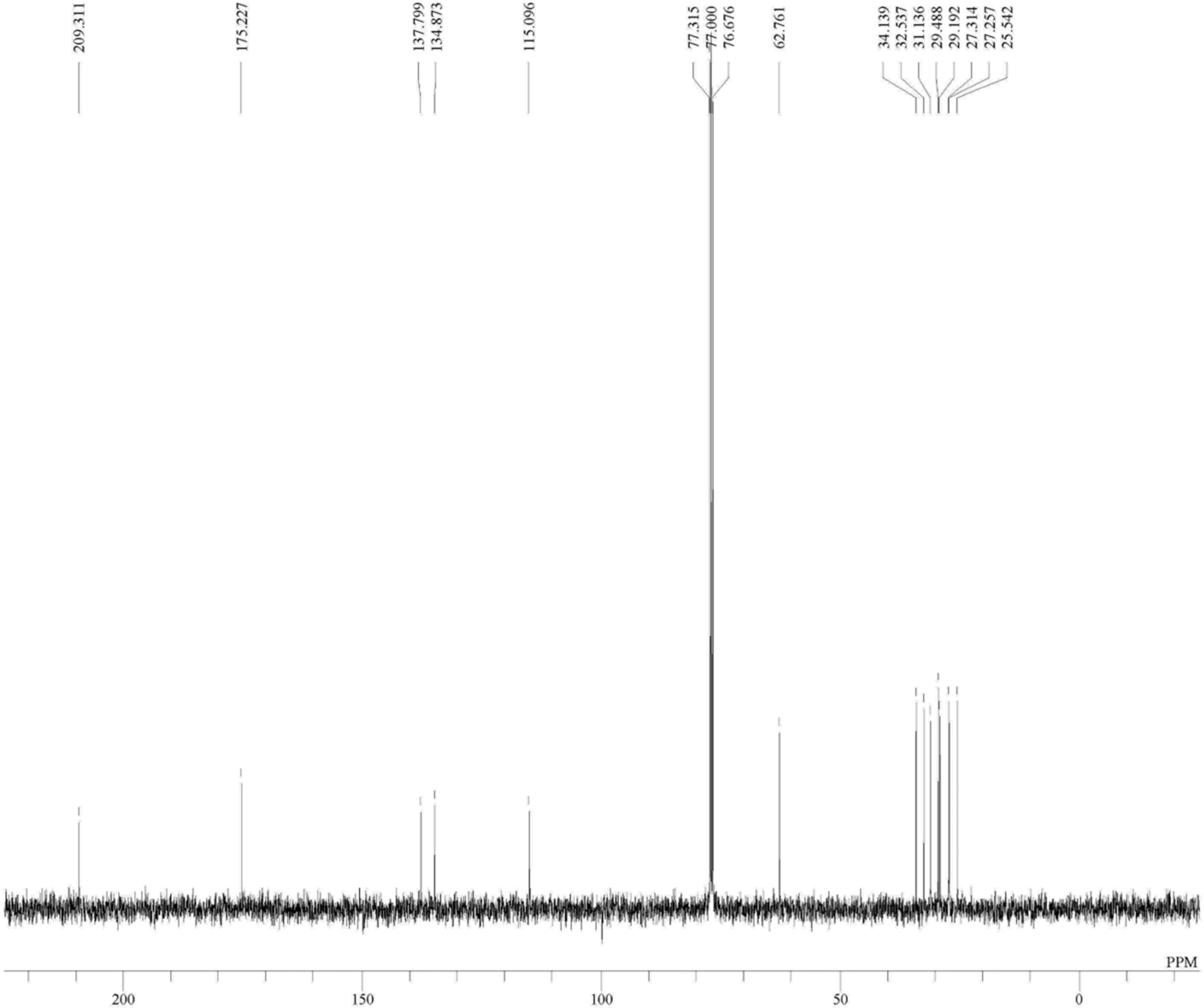

DFILE 33 ƒOƒŠƒjƒƒ [ƒ‹_Carbon-1-1.jd
COMNT single pulse decoupled gated N
DATIM 30-10-2020 20:59:35
OBNUC 13C
EXMOD carbon.jxp
OBFRQ 100.53 MHz
OBSET 5.35 KHz
OBFIN 5.86 Hz
POINT 32780
FREQU 31407.04 Hz
SCANS 74
ACQTM 1.0433 sec
PD 2.0000 sec
PW1 3.37 usec
IRNUC 1H
CTEMP 22.0 c
SLVNT CDCL3
EXREF 77.00 ppm
BF 0.25 Hz
RGAIN 50

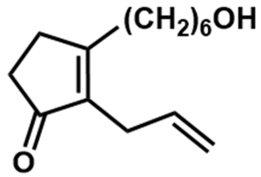

33

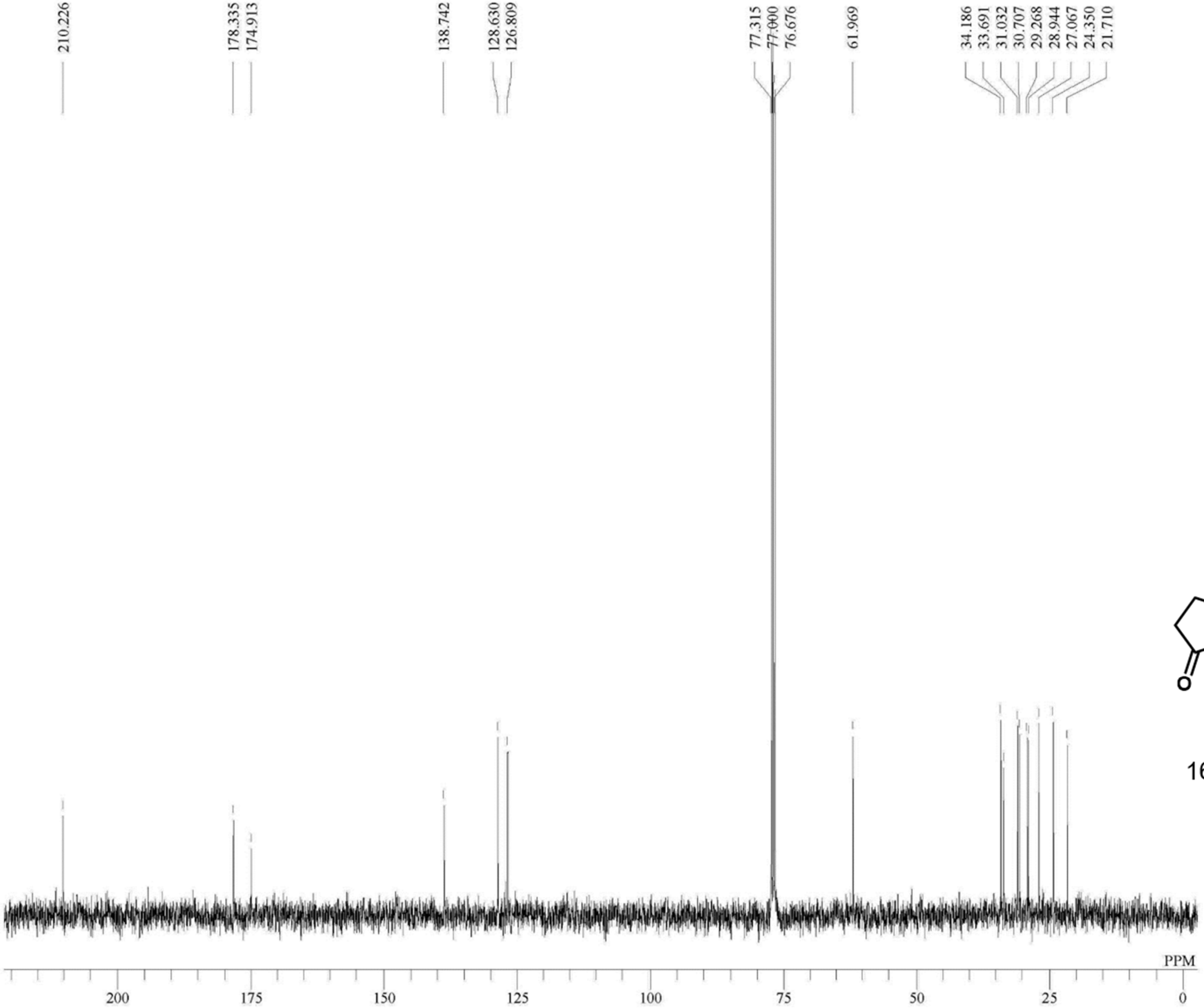

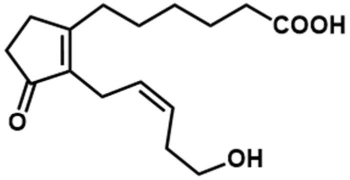

16-OH-dn-*iso*-OPDA (**8**)

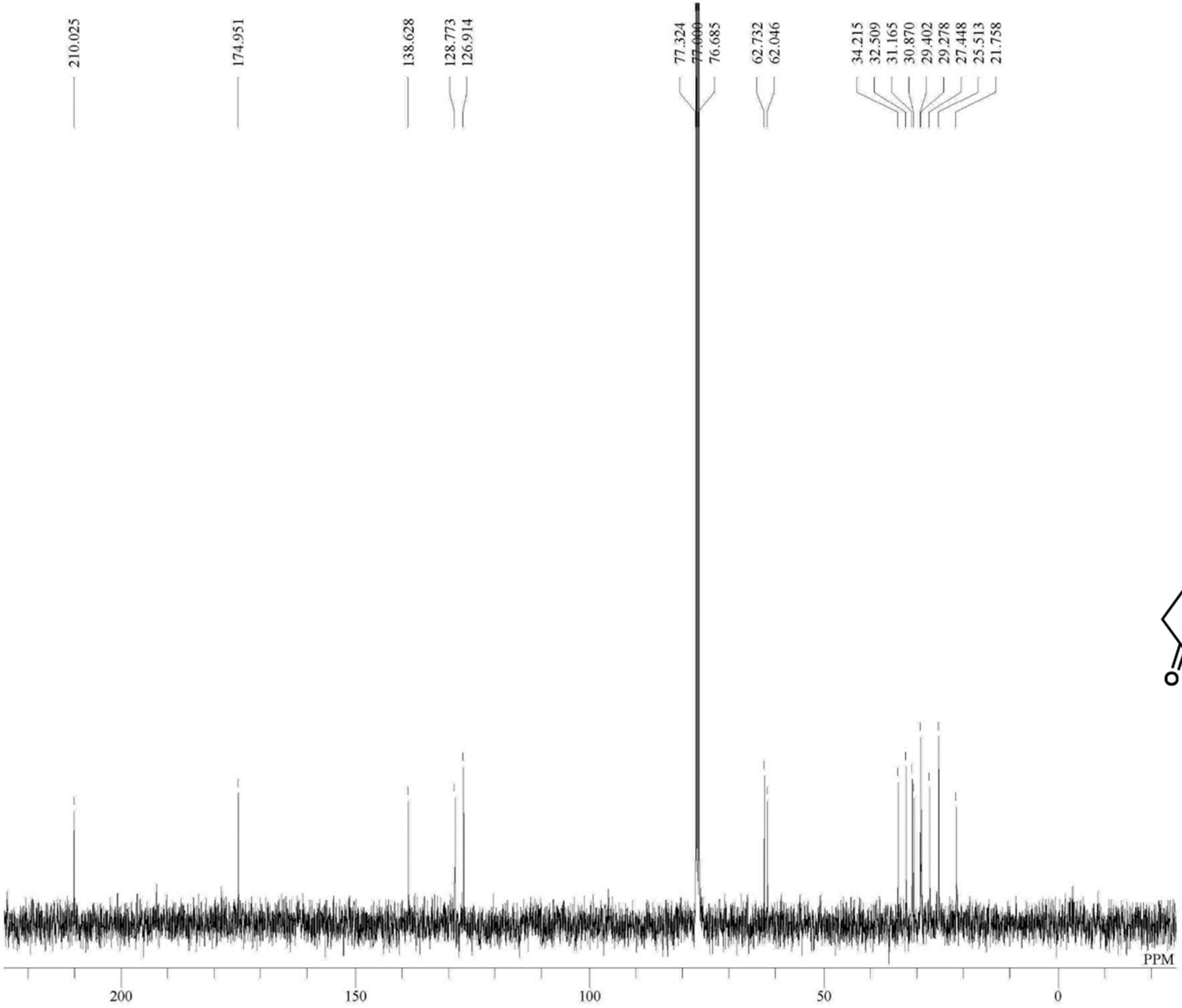

DFILE	35 fWfqfhf fLfV_Carbon-1-1
COMNT	single pulse decoupled gated N
DATIM	30-10-2020 20:43:38
OBNUC	13C
EXMOD	carbon.jxp
OBFRQ	100.53 MHz
OBSET	5.35 KHz
OBFIN	5.86 Hz
POINT	32780
FREQU	31407.04 Hz
SCANS	174
ACQTM	1.0433 sec
PD	2.0000 sec
PW1	3.37 usec
IRNUC	1H
CTEMP	22.0 c
SLVNT	CDCL3
EXREF	77.00 ppm
BF	0.25 Hz
RGAIN	50

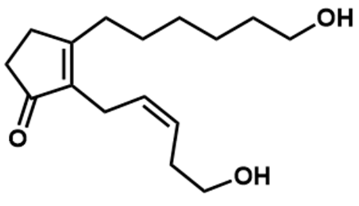

34

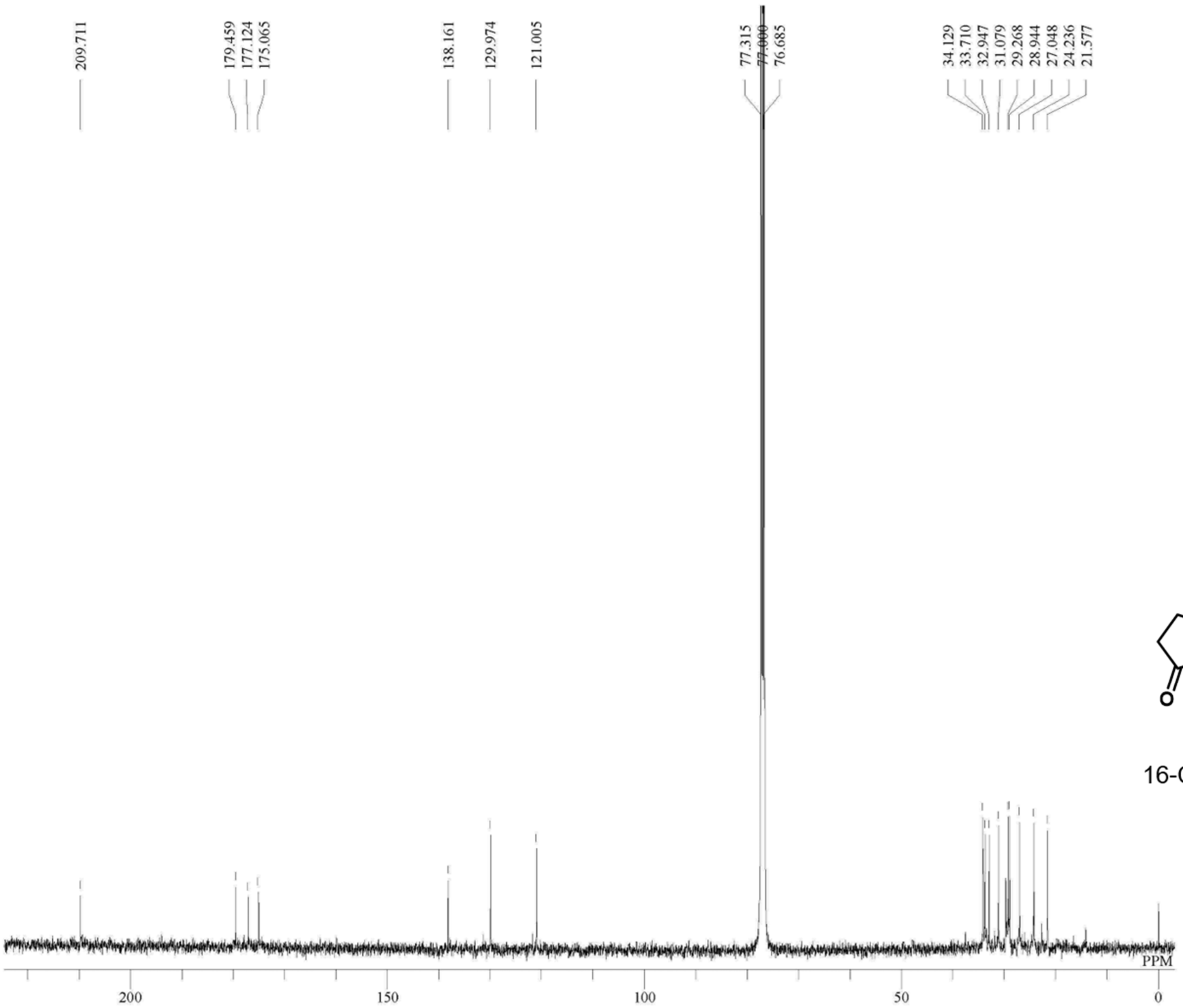

DFILE 07197_Carbon-1-1.jdf
COMNT single pulse decoupled gated N
DATIM 13-12-2020 18:01:10
OBNUC 13C
EXMOD carbon.jxp
OBFRQ 100.53 MHz
OBSET 5.35 KHz
OBFIN 5.86 Hz
POINT 32780
FREQU 31407.04 Hz
SCANS 15000
ACQTM 1.0433 sec
PD 2.0000 sec
PW1 3.37 usec
IRNUC 1H
CTEMP 19.2 c
SLVNT CDCL3
EXREF 77.00 ppm
BF 0.25 Hz
RGAIN 50

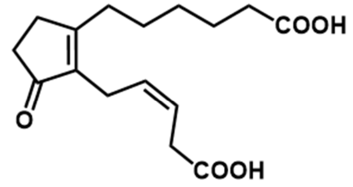

16-COOH-dn-*iso*-OPDA (**9**)
